# Microbial Community and Metabolic Activity in Thiocyanate Degrading Low Temperature Microbial Fuel Cells

**DOI:** 10.3389/fmicb.2018.02308

**Published:** 2018-09-28

**Authors:** Gaofeng Ni, Sebastian Canizales, Elias Broman, Domenico Simone, Viraja R. Palwai, Daniel Lundin, Margarita Lopez-Fernandez, Tom Sleutels, Mark Dopson

**Affiliations:** ^1^Centre for Ecology and Evolution in Microbial Model Systems (EEMiS), Linnaeus University, Kalmar, Sweden; ^2^Wetsus, European Centre of Excellence for Sustainable Water Technology, Leeuwarden, Netherlands

**Keywords:** MFC, thiocyanate degradation, extracellular electron transfer, low temperature, metatranscriptomics

## Abstract

Thiocyanate is a toxic compound produced by the mining and metallurgy industries that needs to be remediated prior to its release into the environment. If the industry is situated at high altitudes or near the poles, economic factors require a low temperature treatment process. Microbial fuel cells are a developing technology that have the benefits of both removing such toxic compounds while recovering electrical energy. In this study, simultaneous thiocyanate degradation and electrical current generation was demonstrated and it was suggested that extracellular electron transfer to the anode occurred. Investigation of the microbial community by 16S rRNA metatranscriptome reads supported that the anode attached and planktonic anolyte consortia were dominated by a *Thiobacillus*-like population. Metatranscriptomic sequencing also suggested thiocyanate degradation primarily occurred via the ‘cyanate’ degradation pathway. The generated sulfide was metabolized via sulfite and ultimately to sulfate mediated by reverse dissimilatory sulfite reductase, APS reductase, and sulfate adenylyltransferase and the released electrons were potentially transferred to the anode via soluble electron shuttles. Finally, the ammonium from thiocyanate degradation was assimilated to glutamate as nitrogen source and carbon dioxide was fixed as carbon source. This study is one of the first to demonstrate a low temperature inorganic sulfur utilizing microbial fuel cell and the first to provide evidence for pathways of thiocyanate degradation coupled to electron transfer.

## Introduction

The toxic compound thiocyanate (SCN^−^) is generated by the mining and metallurgy industries during gold recovery [up to 4000 mg/L (Kantor et al., 2017)] with cyanide and it should be removed from wastewaters before being released to recipient water bodies (Zagury et al., 2004; Van Zyl et al., 2011). Chemical removal of SCN^−^ is both inefficient and costly (Van Zyl et al., 2011). In contrast, biodegradation is relatively inexpensive and can completely remove SCN^−^ and other contaminants such as cyanide (Kantor et al., 2015). However, a complicating factor in treating mining wastewaters in cold climates, such as in northern Sweden, is that the water temperature rarely exceeds approximately 12°C and approaches 0°C in winters (Liljeqvist et al., 2011). As a result, economic constraints require the development of (bio)remediation processes for contaminant removal that operate at low temperatures.

A variety of chemolithotrophic and heterotrophic bacterial genera metabolize SCN^−^, allowing them to be candidate organisms for SCN^−^ removal, including *Thiobacillus* (Happold et al., 1958), *Paracoccus* (Ghosh and Roy, 2007), *Pseudomonas* (Chapatwala et al., 1998), and *Arthrobacter* (Betts et al., 1979). There are two major SCN^−^ degradation pathways and the overall reaction is shown in Eq. 1. In the ‘COS pathway’ (Eqs. 2, 3), SCN^−^ is initially hydrolyzed into ammonia and carbonyl sulfide (COS), and the carbonyl sulfide is subsequently oxidized into carbon dioxide and sulfate in an energy yielding reaction with a key enzyme in this pathway being the cobalt-coordinating thiocyanate hydrolase (Scn) (Katayama et al., 1992, 1993; Kim and Katayama, 2000).

(1) $${\mathrm{SCN}}^{-}+7H_{2}O↔{\mathrm{SO}}_{4}^{2-}+{\mathrm{HCO}}_{3}^{-}+{\mathrm{NH}}_{4}^{+}+9H^{+}+8e^{-}$$

(2) $${\mathrm{SCN}}^{-}+H^{+}+2H_{2}O\overset{\mathrm{Scn}}{\to }\mathrm{COS}+{\mathrm{NH}}_{4}^{+}+{\mathrm{OH}}^{-}$$

(3) $$\mathrm{COS}+H_{2}O\to {\mathrm{CO}}_{2}+H_{2}S$$

The second ‘cyanate pathway’ (Eqs. 4, 5) proposes that SCN^−^ is used as the sole nitrogen and/or sulfur source for growth (Stratford et al., 1994) by initially hydrolyzing it into sulfide and cyanate (OCN^−^). The intermediate cyanate is subsequently converted to ammonium by cyanase (Cyn) and the sulfide is oxidized into sulfate or tetrathionate (Youatt, 1954; Sung and Fuchs, 1988; Stratford et al., 1994).

(4) $${\mathrm{SCN}}^{-}+H_{2}O\to {\mathrm{OCN}}^{-}+H_{2}S$$

(5) $${\mathrm{OCN}}^{-}+{\mathrm{HCO}}_{3}^{-}+3H^{+}\overset{\mathrm{Cyn}}{\to }{\mathrm{NH}}_{4}^{+}+2{\mathrm{CO}}_{2}$$

In a recent metagenomic study, two laboratory-scale SCN^−^ degrading bioreactors showed predominance of *Thiobacillus* spp. with a chemolithotrophic lifestyle (Kantor et al., 2015). Genes encoding the major SCN^−^ degrading enzymes including Scn and Cyn were present (Kantor et al., 2015).

Bioelectrochemical Systems (BESs) are versatile technologies that utilize the interaction of microbial catalysts at an electrode interface. BESs can either recover electrical energy or produce a product, such as methane (Geppert et al., 2016), acetate (Jourdin et al., 2015), copper (Rodenas Motos et al., 2015), or ammonia (Kuntke et al., 2018). Typical electron donors tested in BESs are organic substrates such as acetate, glucose, and synthetic or real wastewaters [reviewed in Pant et al. (2010)]. With few exceptions, the use of inorganic sulfur compounds as substrates are rarely studied. Exceptions include sulfide and tetrathionate from synthetic or industrial wastewaters (Rabaey et al., 2006; Sulonen et al., 2015; Ni et al., 2016). In general, lowering the operational temperature of these systems will negatively impact reaction rates, alter enzyme-substrate interactions, and ultimately lead to protein cold-denaturation (Makhatadze and Privalov, 1994; Georlette et al., 2004). Lower temperatures also reduce the available energy from the desired processes and is why most of these studies are performed in mesophilic conditions (Dopson et al., 2015). Nonetheless, being able to function in cold temperature is a prerequisite should the biotechnology be implemented in areas where winter climates decrease the temperature of wastewaters (Liljeqvist et al., 2011), and low temperature BESs can be a potential remedy for this situation. Apart from saving heating costs, they can harvest a huge potential energy reserve when applied to marine sediments (Bond et al., 2002; Reimers et al., 2006), inactivate competing processes for electrons such as methanogenesis and acetogenesis when organic substrates are utilized (Lu et al., 2011), and improve Coulombic efficiencies (CE) as demonstrated in a study in which hydrogen production at 4°C was achieved with a CE above 92% (Lu et al., 2011). However, low temperature BES studies using inorganic sulfur compounds have not been published and rarely have studies utilized metatranscriptomics to investigate the active microbial species and their metabolic processes.

Metatranscriptomics is the direct extraction and characterization of the total RNA from a community of interest (Zhi et al., 2014), and provides information regarding the active metabolic processes occurring at the time of RNA extraction. It is powerful in profiling the microbial community composition and the metabolic processes of interest. In this study, we report the first endeavor of a such approach to investigate a novel low temperature SCN^−^ degrading microbial consortium able to facilitate electrical current generation in microbial fuel cells (MFCs, a type of BES). The metatranscriptomic data were used to characterize the active microbial community under the engineered conditions, with the focus on the mechanisms for SCN^−^ degradation coupled to extracellular electron transfer (EET) to the electrode.

## Materials and Methods

### MFC Construction, Operation, and Microbial Inoculum

Duplicate MFCs (designated as MFC A and B) were constructed from Plexiglas and the volumes of the anode and cathode chambers were 66 and 33 cm^3^, respectively. The anode had two flow chambers with the inner chamber open on both sides that was filled with a graphite felt material (FMI Composite Ltd., Galashiels, Scotland) while the outer compartment was open on the inner side. These two compartments provided space for the graphite felt and for the electrolyte to flow. The cathode compartment consisted of a single flow chamber sealed with a flat graphite plate. A cation exchange membrane (CMI-7000, Membranes International INC., United States) separated the electrolytes between the anode and cathode compartments. An anaerobic environment was maintained using silicon rubber layers placed on both sides of the flow chambers, being pressed tightly together. The graphite felt was connected to an external cable via a gold wire woven through the felt. Reference electrodes (Ag/AgCl, Sigma-Aldrich Co., LLC; 203 mV vs standard hydrogen electrode) were submerged in 3M KCl solution connected to the anode or cathode electrolyte via glass capillaries (ProSense, Netherlands). The external resistance was initially kept at 1000 Ω and switched to 560 Ω from day 150 to 180 on for MFC A and B, respectively. The cathode, anode, and membrane potentials plus the cell voltage were measured with a digital multimeter (BS3604W, Clas Ohlson, Sweden) on a daily basis. The electrical current production was calculated from the cell voltage and external resistance. The Coulombic efficiency was estimated based on the complete oxidation from thiocyanate to sulfate, according to Eq. 6, where Δ[SCN^−^] was the decrease of thiocyanate in mol; F is the faraday constant (96485 C/mol); and electrical current (I) was expressed in A. The experiment was carried out in a temperature-controlled room maintained at 8°C.

(6) $$\mathrm{CE}(\%)=\frac{\int_{0}^{\text{t}}I\cdot \mathrm{dt}}{\Delta [{\mathrm{SCN}}^{-}]\cdot 8\cdot F}\cdot 100$$

The microbial inoculum was a previously described low temperature, autotrophic denitrifying culture using SCN^−^ as the electron donor (Broman et al., 2017). The anolyte consisted of mineral salts medium (Broman et al., 2017) except that the electron acceptor (nitrate) was excluded, the catholyte consisted of 50 mM potassium ferricyanide. The initial SCN^−^ concentration was approximately 6 mM, a value chosen based on the SCN^−^ concentration in a Boliden AB (Sweden) process water from a sulfide mineral processing plant (Broman et al., 2017). SCN^−^ concentration was analyzed by cyanolysis (Kelly et al., 1969) as described in Dopson and Lindström (1999) without the incubation step with tetrathionate. Additional SCN^−^ was periodically added to the anolyte when the concentration decreased close to zero, carbonate was supplied as the carbon source.

### RNA Extraction and Sequencing

RNA extraction from planktonic cells from the two MFCs was performed at the end of the experiment (i.e., day 272) by sampling the anolyte, immediately mixing (1:1 ratio) with RNA fix solution (Feike et al., 2012), and cell capture on a 0.22 μm filter. RNA extraction from cells attached to the graphite felt was carried out at the same time by immediately soaking the felt in the RNA fixative followed by vigorous shaking of the felt-containing solution and then cell capture with a 0.22 μm filter. Community RNA was extracted from both planktonic and attached cells according to the manufacturer’s instruction using the RNeasy midi kit for isolation of total RNA from Bacteria (Qiagen, Germany). The extracted RNA was used to generate cDNA with the Ovation^®^ RNA-Seq System V2 (NuGEN, United States) according to manufacturer’s instructions. The generated cDNA was purified using MinElute Reaction Cleanup Kit (Qiagen, Germany). Metatranscriptome library preparation was carried out using the Illumina Hiseq Truseq Nano DNA Library Prep Kit for NeoPrep at SciLifeLab. Clustering was done by ‘cBot’ and samples were sequenced on HiSeq2500 (HiSeq Control Software 2.2.58/RTA 1.18.64) with a 2 × 126 bp setup using ‘HiSeq SBS Kit v4’ chemistry. The Bcl to FastQ conversion was performed using bcl2fastq-1.8.4 from the CASAVA software suite. The quality scale used was Sanger / phred33 / Illumina 1.8+.

### Bioinformatics

Details of metatranscriptomic analysis is reported in **Supplementary File 1**. Briefly, the active members of MFC communities were identified through the phylogenetic placement of 16S rRNA reads, which were a subset from the total datasets extracted with SortMeRNA (version 2.1b), using default parameters (Kopylova et al., 2012) and 16S rRNA reference databases provided by the authors on the tool repository^1^. The phylogenetic placement was performed as follows: 16S rRNA reads were aligned to a reference multiple alignment (RMA) with PaPaRa v2.5 using default parameters (Berger and Stamatakis, 2011), then inserted into a related reference phylogenetic tree (RPT) by re-optimization of RPT edge lengths through the Evolutionary Placement Algorithm (EPA) implemented in RAxML (version 8.2.10) (Stamatakis, 2014). Two separate RPTs/RMAs were used for Archaea and Bacteria, respectively, including SSU sequences from Anantharaman et al. (2016) and Hug et al. (2016). Only the most reliable phylogenetic placements (likelihood weight ratio of ≥ 0.90) were considered for downstream analyses. Abundances of reads at each tree node were determined with the guppy version 1.1 utility of the pplacer version 1.1.alpha17 package (Matsen et al., 2010), summarized at the genus level and reported in **Figure 2**.

The mRNA reads were aligned against the NCBI NR database with an *e*-value < 0.001 using Diamond 0.9.10 (Buchfink et al., 2015) in conjunction with BLASTX (Altschul et al., 1990). The read alignments were imported into MEGAN 6 with default LCA-settings (Huson and Mitra, 2012) and classified into taxonomy and proteins based on the MEGAN available protein accession to taxonomy (March, 2018) and InterPro2GO (November, 2016 version) databases. The name of each individual read was used to link taxonomic affiliation with InterPro proteins. Sample counts were normalized among samples as CPM values (counts per million sequences; i.e., (x/sum of sample) × 1 000 000). Statistical testing of the assigned protein classification between the planktonic and electrode-attached communities was conducted using the function exactTest from the edgeR package in R with false discovery threshold of 0.05 (Robinson et al., 2010).

The raw and metatranscriptomic sequences were submitted to the NCBI BioProject database with accession number PRJNA473625.

## Results and Discussion

### Thiocyanate Degradation and Electrical Current Generation

**FIGURE 1:**
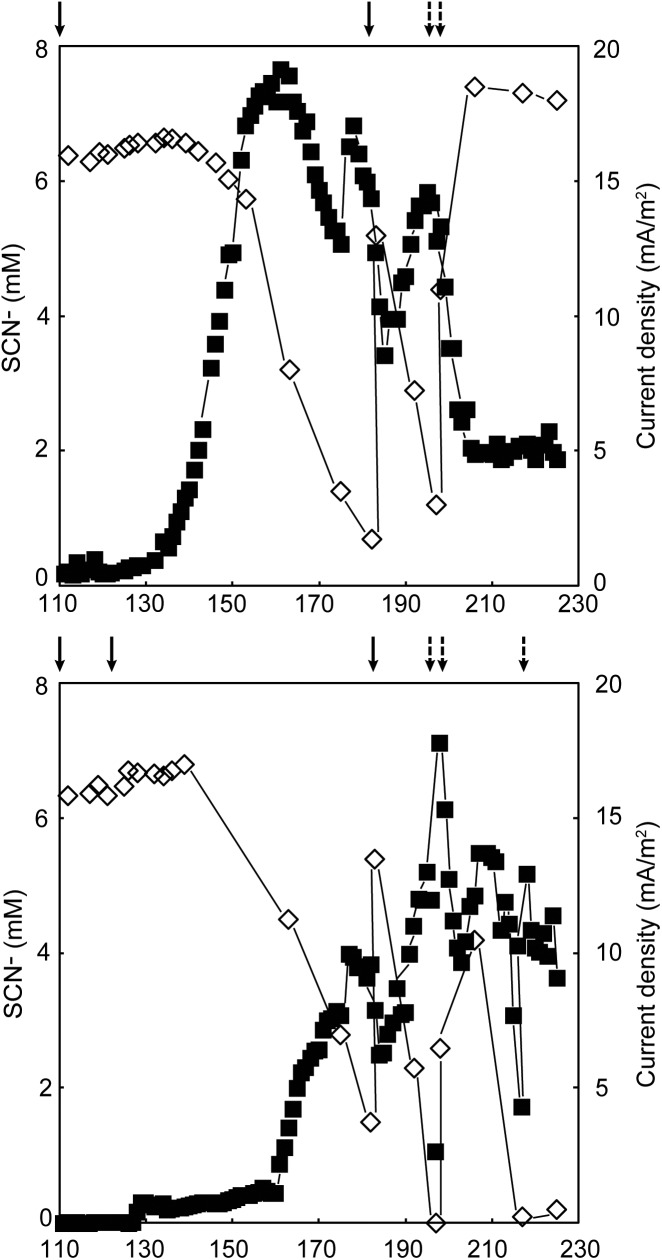
Electrical current production 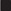 corresponding to the right axis and thiocyanate degradation 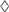 corresponding to the left axis in MFCs **(A)** and **(B)** (top and bottom, respectively). Dashed arrows indicate addition of thiocyanate and the solid arrows designate the MFCs were re-started with fresh anolyte and inoculum. No thiocyanate degradation or electrical current production occurred prior to day 100 and one outlier electrical current value was removed from both MFCs.

Duplicate MFCs were inoculated with a SCN^−^ degrading culture and operated at 8°C for 272 days. Since no electrical current was produced in the first 118 days, potentially due to the insufficient activity from the microbiome, re-inoculation took place on days 93 (MFC B), 94 (MFC A), 99 (both MFCs), and 111 (both MFCs; **Supplementary Files 2**, **3**). An increase in electrical current production occurred on day 119 and 129 for MFCs A and B, respectively (**Figure 1**). Before the increase of current was observed, the concentration of SCN^−^ remained stable in both MFCs (6.37 ± 0.06 mM, *n* = 3 for MFC A; 6.51 ± 0.14 mM, *n* = 8 for MFC B). On experimental day 176, the maximum current density normalized to anode volume was 675.0 and 592.6 mA/m^3^ for MFCs A and B, respectively, that gave 20.2 and 17.8 mA/m^2^ as normalized to the projected anode/membrane surface area. At that time, the SCN^−^ degradation rates for MFCs A and B were 0.123 mM/day and 0.068 mM/day, respectively. In conjunction with the electrical current production, the concentration of SCN^−^ in both systems decreased, indicating the produced electrical current was dependent on the oxidation of SCN^−^. In general, a low temperature negatively impacts on the microbial enzyme machinery and reduces electrochemical reaction rates (Dopson et al., 2015); furthermore, psychrophiles generally have longer doubling times compared to mesophiles (Ferroni et al., 1986). These factors may explain the long start-up time of the electrical current production, which has also been observed in other low temperature BES studies (Bond et al., 2002; Reimers et al., 2006).

Based on Eq. 1, the theoretical anode potential at pH 7 and 8°C was calculated as −476 mV (against Ag/AgCl). For both MFC A and B, the measured anode potential values were more positive than the theoretical (**Supplementary Files 2**, **3**). As electrochemically active microorganism(s) transfer electrons to the electrode, the anode potential would develop toward the more negative theoretical value when the electrical circuit was open (measured as anode ‘open cell potential’). However, since the graphite-felt material had a relatively high electrical capacity and a slow electron transfer to the electrode was caused by low temperature; plus, no biofilm formation was observed which would facilitate electron transfer; it would take time for the anode potential to reach the theoretical value. This was confirmed when MFC A was operated in open cell and the anode potential decreased from 296 to 2 mV after 25.5 h (experimental days 261 – 262) and was still decreasing.

The Coulombic efficiencies between days 111 – 272 were 2.5 and 1.0% for MFCs A and B, respectively, (**Supplementary File 4**). Compared to other psychrophilic BES studies (reviewed in Dopson et al., 2015), both the produced electrical current and Coulombic efficiency were relatively low and could be for several reasons. Firstly, carbon dioxide fixation for biomass growth by some members of the anodic microbial consortium (described below) restricted the available electrons that could be harvested as electrical current. Secondly, although N_2_ gas was continuously flushed in the anolyte circulation bottle, trace levels of oxygen (a competing electron acceptor) can diffuse into the MFC via rubber tubing and graphite plates. Thirdly, competing energy conservation reactions induced by cold stress may have occurred (discussed below).

### Metatranscriptomic Analysis of the Active Community

**FIGURE 2:**
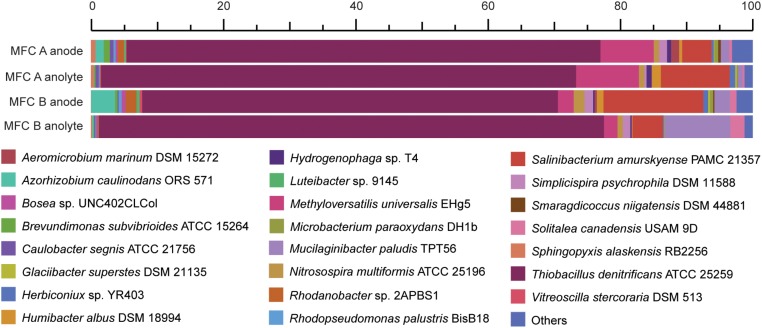
Relative abundance of the two MFCs anode attached and anolyte microbiomes based on high throughput 16S rRNA data. The duplicate MFCs (termed MFC A and MFC B) were producing an electrical current when the cells were harvested and fixed for metatranscriptomic sequencing.

The original inoculum was obtained from an anaerobic SCN^−^ degrading stirred tank bio-reactor (Broman et al., 2017). However, since the terminal electron acceptor (nitrate) was removed in this study, electrons were suggested to pass to the solid electrode surface. Community RNA was extracted from both the planktonic cells and those attached to the graphite felt (electrode) from the replicate MFCs. A total of 170 million read pairs were obtained across the four samples (MFC anode/anolyte plus MFC B anode/anolyte), of which 78% remained after quality trimming. 8% of the trimmed reads were identified as 16S rRNA; 4% of the trimmed reads were identified as mRNA, of which 88% was assigned to a known protein in the InterPro database (**Supplementary File 5**). Although using 16S rRNA data as a proxy for microbial activity has been questioned (Blazewicz et al., 2013), here we use the 16S rRNA read counts to infer a ‘protein synthesis potential’ (Thureborn et al., 2017). The dominant 16S rRNA reads (between 63 and 76%) were aligned to the *Thiobacillus* genus and primarily to *T. denitrificans* across the four samples (**Figure 2**). In addition, 16S rRNA reads also aligned with species including *Methyloversatilis universalis*, *Salinibacterium amurskyense*, *Azorhizobium caulinodans*, and *Mucilaginibacter paludis*. The type species of the genus *Thiobacillus*, *T. thioparus* can grow anaerobically with thiosulfate and nitrate (Katayama and Hiroshi, 1978). The presence of *Thiobacillus* spp. was in accordance with previous studies in which *Thiobacillus* populations were the dominating strains for SCN^−^ degradation (Kantor et al., 2015, 2017), and also suggested a novel electrochemically active trait for populations within this genus.

An abundance of mRNA reads were not taxonomically assigned with better precision than Bacteria (7%) or Proteobacteria (49%) (**Supplementary File 6**). Based on the high activity of *T. denitrificans* according to 16S rRNA analysis (**Figure 2**), these Bacteria and Proteobacteria populations suggested by mRNA analysis were potentially from the *Thiobacillus* genus. Finally, default edgeR analysis gave no statistically significant differences regarding the discussed genes coding for sulfur, carbon, nitrogen metabolism as well as electron transfer and adaptation to low temperature between the electrode-attached and planktonic communities based on mRNA analysis (**Supplementary File 6**).

### Thiocyanate Degradation and Oxidation of Inorganic Sulfur Compound Intermediates

**FIGURE 3:**
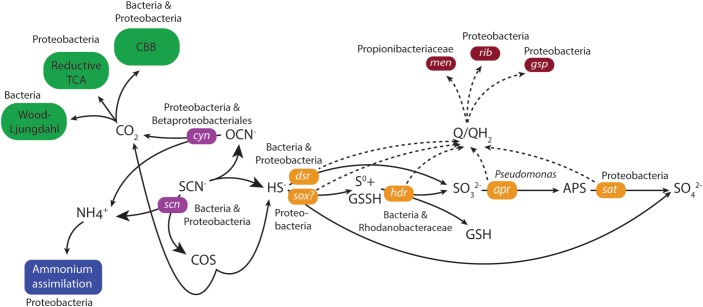
Model of thiocyanate degradation together with sulfur, carbon and nitrogen metabolism coupled to EET based upon mRNA data. Solid lines indicate the flow of metabolites and dashed lines the flow of electrons. The primarily assigned populations based upon mRNA data were indicated. Green indicates CO_2_ fixation, blue indicates ammonium assimilation, purple indicates thiocyanate degradation, orange indicates sulfur metabolism, and dark red indicates electron transfer.

Genes coding for both the ‘COS’ and ‘cyanate’ pathways for thiocyanate degradation to sulfide were identified in the duplicate MFCs (**Figure 3** and **Supplementary File 6**). The key gene in the ‘COS pathway’ is thiocyanate hydrolase (*scn*; Kantor et al., 2015) that was attributed to the unclassified Bacteria (0 – 67 CPM) and unclassified Proteobacteria (0 – 53 CPM) across the four samples. However, mRNA reads for the second gene in the ‘COS’ pathway (carbonyl sulfide hydrolase) were not identified in either of the MFCs. Although mRNA reads for thiocyanate dehydrogenase that catalyzes the first stage in the ‘cyanate’ pathway was not identified, the key cyanase gene (*cyn*) in this pathway (Kantor et al., 2015) was present. The majority of mRNA reads for *cyn* were attributed to unclassified Proteobacteria (9 – 284 CPM) and Betaproteobacteriales (3 – 151 CPM) and with lower numbers of mRNA reads assigned to e.g., a *Thiobacillus* population. Of the key genes for the two pathways, the greater number of mRNA reads for cyanase (Cyn) suggested a higher activity of the ‘cyanate’ pathway.

The generated sulfide from thiocyanate degradation was suggested to be oxidized by the highly expressed dissimilatory sulfite reductase (Dsr) that functions in reverse to produce sulfite (Schedel and Trüper, 1979; Kantor et al., 2015). mRNA reads for *dsr* were predominantly attributed to either the unclassified Bacteria (4387 – 7294 CPM) or unclassified Proteobacteria (2275 – 5914 CPM) in the four metatranscriptomes (**Figure 3** and **Supplementary File 6**). The resultant sulfite can then be metabolized to form adenosine 5′-phosphosulfate catalyzed by adenylylsulphate reductase (Apr) (Kappler and Dahl, 2001) and subsequently to sulfate via the membrane-bound dissimilatory adenylyl-sulfate reductase (Sat) (Peck, 1962). A higher number of mRNA reads were identified for *sat* (543 – 4255 CPM) than for *apr* (< 27 CPM in both MFCs) attributed to unclassified Proteobacteria. The gene for sulfur carrier accessory protein (*tus*) is commonly found together with *dsr* (Stockdreher et al., 2014) and its mRNA reads were primarily attributed to unclassified Proteobacteria (375 – 754 CPM). An alternative sulfide oxidation pathway was also present, involving a truncated *sox* complex (*soxABCXY*) primarily attributed to unclassified Proteobacteria and unclassified Bacteria that could oxidize sulfide generating either sulfur or sulfate (Kantor et al., 2017). Finally, a very low level of mRNA reads were identified for the anaerobic elemental sulfur oxidizing *hdr* in MFC A (Osorio et al., 2013).

### Energy Conservation and Electron Transfer

The generation of an electrical current alongside thiocyanate degradation after a lag phase of more than 100 days strongly suggested electrons from thiocyanate and intermediate inorganic sulfur compound oxidation were transported to the anode facilitated by the anodic microbiome. No genetic pathways for carrying out extracellular transfer (EET) in the *Thiobacillus* genus are reported and therefore, the potential presence in our MFCs of known genes and pathways for EET in the extensively studied genera *Geobacter* and *Shewanella* were investigated. Outer membrane multi-heme *c*-type cytochromes encoded by genes such as *mtrAC*, *cymA*, and *omcAESBZ* in *Shewanella* and *Geobacter* species are critical in carrying out electron transfer from the microorganisms to electrodes in MFCs (Gorby et al., 2006; Holmes et al., 2006; Bretschger et al., 2007; Shi et al., 2007; Richter et al., 2009). In addition, the capacity of *Shewanella* and *Geobacter* to produce an electrical current is severely impaired when the type II secretion and type IV pilin biosynthesis pathways that involves the genes *gspDG* and *pilAD* are deficient (Reguera et al., 2005; Gorby et al., 2006; Bretschger et al., 2007; Richter et al., 2009; Vargas et al., 2013). Finally, although they have other functions within the cell (Wissenbach et al., 1990; Vitreschak et al., 2002), redox active electron shuttles such as menaquinone and quinone intermediates encoded by the *menC* gene, as well as riboflavin have been shown to be crucial in the ability to perform EET in *Shewanella* species (Newman and Kolter, 2000; Myers and Myers, 2004; Marsili et al., 2008). The mRNA data did not support EET involving known *c*-type cytochromes or conductive pili. mRNA reads for the type II secretion pathway genes *gspDEGHM* were present, primarily attributed to unclassified Proteobacteria (180 – 900 CPM; **Figure 3** and **Supplementary File 6**) or *Thiobacillus* (3 – 359 CPM). mRNA reads for the type IV pilin genes *pilAD* were absent, but mRNA reads for a pilin fimbrial protein were mainly attributed to a *Rhodanobacter* sp. (15 – 154 CPM) and unclassified Proteobacteria (15 – 110 CPM). mRNA reads for menaquinone biosynthesis genes *menBC* were primarily assigned to the family Propionibacteriaceae (0 – 183 CPM) while mRNA reads for riboflavin biosynthesis proteins RibABD were mostly assigned to unclassified Proteobacteria (111 – 291 CPM). The presence of mRNA reads for type II secretion as well as menaquinone plus riboflavin biosynthesis suggested the anodic microbial community could have carried out EET using soluble electron shuttles. This claim was supported by the decrease and subsequent recovery in current density upon the removal and addition of new medium during operation of the duplicate MFCs (**Figure 1** and **Supplementary Files 2**, **3**). However, it cannot be ruled out that other, presently unknown mechanisms were responsible for EET in the selected community.

### Ammonium Assimilation and Inorganic Carbon Fixation

The proposed thiocyanate conversion to ammonium by both degradation pathways raises the possibility of energy conservation by ammonium oxidation coupled to sulfate reduction (Fdz-Polanco et al., 2001). However, the suggested predominance of the ‘cyanate pathway’ whereby SCN^−^ is used as the sole nitrogen source for growth (Stratford et al., 1994); the lack of mRNA reads for known ammonium oxidation genes; and no observed mRNA reads for the *dsrK* gene that codes for energy conservation during sulfate reduction all suggested that this did not occur. Instead, the ammonium was likely used as a nitrogen source and assimilated primarily by Type I glutamine synthetase for which mRNA reads were mostly assigned to unclassified Proteobacteria (539 – 985 CPM; **Figure 3** and **Supplementary File 6**) followed by Microbacteriaceae (Actinobacteria, 53 – 169 CPM).

All described species in the *Thiobacillus* genus fix carbon dioxide for cellular growth (Boden et al., 2017) and reconstruction of the thiocyanate degrading *Thiobacillus*-like species genome contained genes coding for the Calvin-Benson-Bassham (CCB) cycle (Kantor et al., 2015). However, mRNA reads encoding the key CBB cycle enzyme ribulose biphosphate carboxylase (RuBisCO) were missing although seven other genes in the cycle (that also have other functions) encoding phosphoglycerate kinase, erythrose phosphate dehydrogenase, and triosephosphate isomerase had CPMs mostly attributed to unclassified Proteobacteria (**Figure 3** and **Supplementary File 6**). In addition, the key enzyme ATP-citrate lyase in the reductive TCA cycle was present; carbon monoxide dehydrogenase from the Wood-Ljungdahl pathway (0 – 248 CPM) were identified in unclassified Bacteria. This suggested that several carbon fixation pathways were present and despite all described *Thiobacillus* species using the CBB pathway, it was not clearly demonstrated for the MFC communities.

### Adaptation to Low Temperature

Along with reduced abiotic reaction and diffusion rates, low temperatures affect microbes by hindering folding of proteins into their three-dimensional structure, coiling and uncoiling DNA, and over-stabilization of mRNA (D’Amico et al., 2006; Casanueva et al., 2010; De Maayer et al., 2014). In response, psychrophiles use a number of strategies to combat low temperature although in many cases, these systems also have other functions within the cell. Cold shock proteins (CSPs) and cold-inducible proteins (CIPs) overlap with each other and are expressed during both short and long-term exposure to cold (Phadtare and Inouye, 2004; Horn et al., 2007; Barria et al., 2013). One example of CSPs and CIPs are chaperones that remove low temperature related mRNA and DNA secondary structures and thus, aid ribosomes and RNA polymerases to function. In addition to CSPs and CIPs, psychrophiles respond to low temperature by producing compatible solutes that protect against freezing (Casanueva et al., 2010) and by altering their membrane structure to increase flexibility (Chintalapati et al., 2004).

The identified cold adaptation systems included mRNA reads for the CspA (Phadtare and Inouye, 2008) and Clp protease (Skinner and Trempy, 2001) that were mostly attributed to unclassified Proteobacteria (479 – 1113 and 1410 – 3435 CPM, respectively; **Figure 3** and **Supplementary File 6**). In addition, mRNA reads were identified for several molecular chaperones including Trigger factor (Tig; 797 – 5124 CPM) that functions together with GroEL (Kandror and Goldberg, 1997) and the SecB protein export chaperone (47 – 158 CPM), both were mostly identified from unclassified Proteobacteria. mRNA reads for the compatible solute synthesis enzyme betaine aldehyde dehydrogenase (BetB; 142 – 998 CPM) were identified from unclassified Proteobacteria and a glycine betaine ABC transporter were primarily identified from Actinobacteria (ProVWX, 0 – 179 CPM). mRNA reads coding for proteins involved in transcription and translation include DeaD RNA helicases that were identified from several populations with the Actinobacteria. NusA (293 – 1365 CPM) that involves in termination and anti-termination of transcription (Jones and Inouye, 1994), and DNA gyrase (516 – 2006 CPM) that relieves the strain of double stranded DNA being unwound (Morais Cabral et al., 1997) were primarily attributed to unclassified Proteobacteria. Finally, mRNA reads from several membrane alteration genes that were mainly attributed to unclassified Proteobacteria and in the case of N-acetylglucosaminyl transferase, also to the *Thiobacillus* genus, suggested that cold induced alteration to the cell membrane occurred. The identification of mRNA reads for cold adaptation suggested the cells were under stress in the low temperature MFCs and this may have contributed to the low Coulombic efficiency.

## Conclusion

In this study, the extraction and analysis of 16S rRNA reads from the metatranscriptomes suggested that *Thiobacillus* was the most abundant and active genus. Metatranscriptomic analysis suggested that the anodic microbial consortium could degrade thiocyanate while the resultant sulfide was oxidized for energy conservation; ammonium was assimilated; and carbon dioxide was fixed via various pathways. It was also revealed that the consortium potentially utilized multiple mechanisms to acclimate to the low temperature including CSPs, cold inducible proteins, and molecular chaperones. Based on mRNA analysis from the metatranscriptomes, these processes were primarily associated with Bacterial and Proteobacteria populations, potentially attributed to *Thiobacillus*. Furthermore, mRNA analysis revealed no significant difference between the planktonic and electrode-attaching communities. Finally, our findings demonstrated for the first time, that an autotrophic psychrophilic microbial consortium facilitated electrical current generation from thiocyanate degradation in a microbial fuel cell. The *Thiobacillus* population potentially capable of carrying out EET enriched the inventory of electrochemically active microorganisms, these insights could benefit future industrial-scale low temperature remediation of thiocyanate, as well as a more diverse application of BESs.

## Author Contributions

GN and MD designed the study. SC and GN defined materials and configuration of the MFCs. SC and GN maintained and analyzed the long-term operation of the MFCs. GN and ML-F extracted nucleic acids. GN, DS, EB, VP, and DL designed and carried out bioinformatic analyses. GN, MD, and TS interpreted the data. GN, MD, DS, and EB drafted the manuscript. All authors read and approved the manuscript for submission.

## Conflict of Interest Statement

The authors declare that the research was conducted in the absence of any commercial or financial relationships that could be construed as a potential conflict of interest.

## Footnotes

1 https://github.com/biocore/sortmerna
