# Supplementary material for: Microbial Community and Metabolic Activity in Thiocyanate Degrading Low Temperature Microbial Fuel Cells
Source: Front Microbiol. 2018 Sep 28;9:2308. doi: 10.3389/fmicb.2018.02308 (PMC6172326; doi:10.3389/fmicb.2018.02308)
Supplement: Supplementary file 1 [file Data_Sheet_1.docx]

Supplementary Material

Microbial Community and Metabolic Activity in Thiocyanate Degrading Low Temperature Microbial Fuel Cells

Gaofeng Ni^1,*^, Sebastian Canizales^1,†^, Elias Broman^1^, Domenico Simone^1^, Viraja R. Palwai^1^, Daniel Lundin^1^, Margarita Lopez-Fernandez^1,#^, Tom Sleutels^2^, Mark Dopson^1^

^1^Centre for Ecology and Evolution in Microbial Model Systems (EEMiS), Linnaeus University, Kalmar, Sweden

^2^Wetsus, European Centre of Excellence for Sustainable Water Technology, Oostergoweg 9, P.O. Box 1113, Leeuwarden 8911 MA, The Netherlands

*** Correspondence:**Gaofeng Ni
[gaofeng.ni@lnu.se](mailto:gaofeng.ni@lnu.se)

**^†^ Present address:** Wetsus, European Centre of Excellence for Sustainable Water Technology, Oostergoweg 9, P.O. Box 1113, Leeuwarden 8911 MA, The Netherlands

**^#^ Present address:** Helmholtz-Zentrum Dresden-Rossendorf, Bautzner Landstraße 400, 01328 Dresden, Germany

**Supplemental File 1.** Details of the metatranscriptomic analysis and scripts used (in italic).

1. **Quality control of the Raw metatranscriptomic reads**

FastQC run with a single command line on all sequence datasets.

*fastqc -o ${outdir} ${infile_regular_expression}*

1. **Quality filtering of reads with Trimmomatic**

*java -jar $TRIMMOMATIC_HOME/trimmomatic.jar PE \*

*-phred33 -threads $(nproc) -trimlog ${infile}.trimlog \*

*-basein ${infile} -baseout ${infile_base} \*

*ILLUMINACLIP:$TRIMMOMATIC_HOME/adapters/TruSeq3-PE.fa:2:30:10 \*

*LEADING:20 TRAILING:20 SLIDINGWINDOW:4:25 MINLEN:100*

1. **Phylogenetic placement of the 16S rRNA reads from metatranscriptome datasets**

3.1 Extract 16S reads (Bacteria) with SortMeRNA

*sortmerna --ref /silva-bac-16s-id90.fasta,/silva-bac-16s-id90 \*

*--reads ${sample}.interleaved.fastq \*

*--aligned ${sample}_sortmerna_aligned_bacSSU.allreads \*

*--paired_in --fastx --log \*

*--num_alignments 1 \*

*--sam \*

*-a 16 -e 1e-20*

3.2 Extract 16S reads (Archaea) with SortMeRNA

*sortmerna --ref /silva-arc-16s-id95.fasta,/silva-arc-16s-id95 \*

*--reads ${sample}.interleaved.fastq \*

*--aligned ${sample}_sortmerna_aligned_arcSSU.allreads \*

*--paired_in --fastx --log \*

*--num_alignments 1 \*

*--sam \*

*-a 16 -e 1e-20*

3.3 Align 16S bacterial reads against reference alignment (*bacRT*) with PaPaRa, using the bacterial reference tree (*bacRT*) as guide.

*papara \*

*-j 16 \*

*-t $(basename ${bacRT}) \*

*-s $(basename ${bacRA}) \*

*-q ${sample}_sortmerna_aligned_bacSSU.allreads \*

*-n ${sample}_sortmerna_aligned_bacSSU.allreads*

3.4 Align 16S archaeal reads against reference alignment (*arcRT*) with PaPaRa, using the archaeal reference tree (*arcRT*) as guide

*papara \*

*-j 16 \*

*-t $(basename ${arcRT}) \*

*-s $(basename ${arcRA}) \*

*-q ${sample}_sortmerna_aligned_arcSSU.allreads \*

*-n ${sample}_sortmerna_aligned_arcSSU.allreads*

3.5 Perform phylogenetic placement of bacterial 16S reads on the bacterial reference tree (*bacRT*) with RAxML-EPA, using as input the PaPaRa output (reads aligned against the reference alignment). PaPaRa output name is *papara_alignment.${sample}_sortmerna_aligned_bacSSU.allreads*

*raxmlHPC-PTHREADS-AVX -f v \*

*-s ${papara_alignment.${sample}_sortmerna_aligned_bacSSU.allreads \*

*-G 0.1 \*

*-t ${bacRT} \*

*-m GTRCAT \*

*-n ${papara_alignment.${sample}_sortmerna_aligned_bacSSU.allreads \*

*-T 8*

3.6 Perform phylogenetic placement of archaeal 16S reads on the archaeal reference tree (*arcRT*) with RAxML-EPA, using as input the PaPaRa output (reads aligned against the reference alignment). PaPaRa output name is papara_alignment.${sample}_sortmerna_aligned_arcSSU.allreads

*raxmlHPC-PTHREADS-AVX -f v \*

*-s papara_alignment.${sample}_sortmerna_aligned_arcSSU.allreads \*

*-G 0.1 \*

*-t ${arcRT} \*

*-m GTRCAT \*

*-n papara_alignment.${bacsample}_sortmerna_aligned_arcSSU.allreads \*

*-T 8*

1. **Functional annotation and relative abundance analysis of the mRNA reads**

4.1 Extract mRNA reads from metatranscriptome datasets using SortMeRNA

*sortmerna --ref $SORTMERNA_DBS/rRNA_databases/rfam-5.8s-database-id98.fasta,$SORTMERNA_DBS/index/rfam-5.8s-database-id98:$SORTMERNA_DBS/rRNA_databases/rfam-5s-database-id98.fasta,$SORTMERNA_DBS/index/rfam-5s-database-id98:$SORTMERNA_DBS/rRNA_databases/silva-arc-16s-id95.fasta,$SORTMERNA_DBS/index/silva-arc-16s-id95:$SORTMERNA_DBS/rRNA_databases/silva-arc-23s-id98.fasta,$SORTMERNA_DBS/index/silva-arc-23s-id98:$SORTMERNA_DBS/rRNA_databases/silva-bac-16s-id90.fasta,$SORTMERNA_DBS/index/silva-bac-16s-id90:$SORTMERNA_DBS/rRNA_databases/silva-bac-23s-id98.fasta,$SORTMERNA_DBS/index/silva-bac-23s-id98:$SORTMERNA_DBS/rRNA_databases/silva-euk-18s-id95.fasta,$SORTMERNA_DBS/index/silva-euk-18s-id95:$SORTMERNA_DBS/rRNA_databases/silva-euk-28s-id98.fasta,$SORTMERNA_DBS/index/silva-euk-28s-id98 --num_alignments 1 --fastx --reads sample_R1.PwU.qtrim.fastq --aligned rRNA/rRNA_sample_R1 --other other/other_sample_R1*

4.2 For the other_RNA sequences (e.g. mRNA), run the diamond aligner with blastx against the NCBI NR (protein) database

*diamond blastx -d $DIAMOND_NR -p 12 -e 0.001 -q sample_R1.fastq -a read1_output*

4.3 Importing to MEGAN, and extracting data

Downloaded all the reference databases on the MEGAN website: <https://ab.inf.uni-tuebingen.de/software/megan6> (at the time of writing).

- Choose Meganze .daa file to import.
- Compare the absolute counts of the sample (include both R1 and R2) and include all unassigned reads.
- Export all the read names and their affiliated taxonomical classification.
- Export the files to a new directory named after the sample (each sample should have its own directory)
- Taxa viewer: Tree -> uncollapse all
- Taxa viewer: Select -> all nodes (nodes because some taxa cannot be classified to species)
- Export to text file, with the settings: readname_to_taxonpath, assigned, tab. Filename should be: reads_to_taxa.txt
- Export all the read names and their affiliated functional classification using InterPro to GO database
- In2go viewer) Tree -> uncollapse all
- In2go viewer) Select -> all leaves
- File -> export to text file, with the settings: readname_to_interpro2gopath, assigned, tab.
  1. Combine the output from MEGAN in R

Import the tables and give the column names

*reads_to_taxa <- read.table("reads_to_taxa.txt", sep="\t")*

*reads_to_ip2go <- read.table("reads_to_ip2go.txt", sep="\t")*

*names(reads_to_taxa)=c("read","taxa")*

*names(reads_to_ip2go)=c("read","ip2go")*

Merge the two tables based on the first column (i.e. name of the read), and name the columns in the new merged table:

*taxa_to_ip2go <- merge(reads_to_taxa,reads_to_ip2go,by.x=1,by.y=1,, all=T)*

*names(taxa_to_ip2go)=c("read","taxa","ip2go")*

Make counts of rows with similar names

*library("plyr")*

*summed_tax_ip2go <- count(taxa_to_ip2go, c("taxa","ip2go"))*

Write the table as .txt files

*write.table(summed_tax_ip2go, file="taxa_to_ip2go_counts.txt", sep="\t")*

For each sample combine the tables in Excel (using the pivot function). And e.g. calculate CPM (counts per million) or relative proportion based on all bacteria and eukaryotes. Discard the data might still contain the PhiX control sequence (seen as ip2go classifications for viruses).

1. **Differential expression analysis of the mRNA reads**

5.1 Prepare R by installing the following packages, follow this step by step:

*> source("*[*http://bioconductor.org/biocLite.R*](http://bioconductor.org/biocLite.R)*")
> biocLite("edgeR")
> biocLite("DESeq")
> biocLite("ctc")
> biocLite("qvalue")
> biocLite("Biobase")*

5.2 Create the file 'samples_described.txt' and add the following information in it:

Anode MFC A
Anode MFC B
Anolyte MFC A
Anolyte MFC B

5.3 Run differential expression analysis using edgeR (with the help of a Trinity script)

*TRINITY_HOME/Analysis/DifferentialExpression/run_DE_analysis.pl --matrix counts_data.txt --method edgeR --samples_file samples_described.txt*

5.4 Find statistically significant proteins

Download the DE_results from the edgeR analysis and look for False Discovery Rate (FDR) values below 0.05. These proteins are statistically significant (p-values needs to be adjusted due to the huge amount of statistical tests conducted and FDR is this adjusted p-value).

**Supplemental File 2.** Electrochemical parameters of MFC A as well as actions taken over the entire experimental period.

| Days | Anode Potential (mV) | Cathode Potential (mV) | Cell Voltage (mV) | External resistance (Ω) | Current density (mA/m^2^) | SCN- (mM) | Comment |
| --- | --- | --- | --- | --- | --- | --- | --- |
| 1 | --- | --- | --- | --- | --- | 2.39 | Start of experiment with new medium. set-up and inoculation |
| 2 | --- | --- | --- | --- | --- | 2.68 |  |
| 8 | --- | --- | --- | --- | --- | 2.62 |  |
| 9 | --- | --- | --- | --- | --- | 2.62 |  |
| 16 | --- | --- | --- | --- | --- | 2.69 |  |
| 17 | --- | --- | --- | --- | --- | 2.69 |  |
| 20 | --- | --- | --- | --- | --- | 2.59 |  |
| 21 | --- | --- | --- | --- | --- | 2.59 |  |
| 22 | --- | --- | --- | --- | --- | 2.55 |  |
| 23 | --- | --- | --- | --- | --- | 2.49 |  |
| 24 | --- | --- | --- | --- | --- | 2.46 |  |
| 27 | --- | --- | --- | --- | --- | 2.38 |  |
| 27 | --- | --- | --- | --- | --- | --- |  |
| 28 | --- | --- | --- | --- | --- | 2.34 |  |
| 29 | --- | --- | --- | --- | --- | 2.27 |  |
| 30 | --- | --- | --- | --- | --- | 2.29 |  |
| 31 | --- | --- | --- | --- | --- | --- |  |
| 34 | --- | --- | --- | --- | --- | 2.18 |  |
| 35 | --- | --- | --- | --- | --- | --- |  |
| 36 | --- | --- | --- | --- | --- | --- |  |
| 38 | --- | --- | --- | --- | --- | 1.93 |  |
| 44 | --- | --- | --- | --- | --- | 1.56 |  |
| 47 | --- | --- | --- | --- | --- | 1.12 | Medium replenishment. replacement of graphite felt. re-inoculation from back-up culture. and flow rate adjustment to 10 mL/min |
| 48 | 167 | 184 | --- | 1000 | 0.00 | --- |  |
| 49 | 149 | 160 | --- | 1000 | 0.00 | 0.90 | Addition of 5 mL SCN^-^ stock solution |
| 50 | --- | --- | --- | 1000 | 0.00 | 2.69 |  |
| 51 | 168 | 224 | 0 | 1000 | 0.00 | 2.55 |  |
| 52 | 169 | 224 | 0 | 1000 | 0.00 | --- |  |
| 54 | 133 | 186 | 0 | 1000 | 0.00 | --- |  |
| 55 | --- | --- | --- | --- | --- | --- |  |
| 55 | --- | --- | --- | --- | --- | --- |  |
| 56 | 75 | 123 | 0 | 1000 | 0.00 | 0.86 |  |
| 57 | 71 | 119 | 0 | 1000 | 0.00 | --- |  |
| 58 | 288 | 342 | 0 | 1000 | 0.00 | --- |  |
| 58 | 289 | 343 | 0 | 1000 | 0.00 | --- |  |
| 62 | 337 | 399 | 0 | 1000 | 0.00 | 0.06 | Nitrogen replacement delay |
| 63 | 332 | 395 | 0 | 1000 | 0.00 | --- | Nitrogen supply restarted |
| 64 | 333 | 396 | 0 | 1000 | 0.00 | --- |  |
| 93 | --- | --- | --- | --- | --- | --- |  |
| 94 | --- | --- | --- | --- | --- | --- | Medium replenishment. replacement of graphite felt. and re-inoculation from back-up culture |
| 95 | --- | --- | --- | --- | --- | --- |  |
| 96 | 311 | 407 | 2 | 1000 | 0.00 | --- |  |
| 97 | 308 | 396 | 2 | 1000 | 0.00 | --- |  |
| 98 | 320 | 411 | 1 | 1000 | 0.00 | --- |  |
| 99 | 306 | 390 | 1 | 1000 | 0.00 | 6.39 | Medium replenishment and re-inoculation from back-up culture |
| 101 | 309 | 392 | 1 | 1000 | 0.00 | 6.37 |  |
| 102 | --- | --- | --- | --- | --- | --- |  |
| 103 | 311 | 393 | 1 | 1000 | 0.00 | 6.42 |  |
| 104 | 306 | 384 | 1 | 1000 | 0.00 | --- |  |
| 105 | 306 | 384 | 1 | 1000 | 0.00 | 6.42 |  |
| 106 | 310 | 387 | 1 | 1000 | 0.00 | --- | Constant nitrogen supply restarted |
| 107 | 305 | 384 | 1 | 1000 | 0.00 |  |  |
| 108 | 320 | 400 | 1 | 1000 | 0.00 | 6.44 |  |
| 109 | 316 | 396 | 1 | 1000 | 0.00 | --- |  |
| 110 | 321 | 397 | 1 | 1000 | 0.00 | --- |  |
| 111 | 326 | 401 | 1 | 1000 | 0.00 | --- | Re-inoculation from back-up culture |
| 112 | 332 | 410 | 1.2 | 1000 | 0.00 | 6.38 |  |
| 113 | 320 | 394 | 0.9 | 1000 | 0.00 | --- |  |
| 114 | 329 | 405 | 2 | 1000 | 0.00 | --- |  |
| 115 | 328 | 404 | 1 | 1000 | 0.00 | --- |  |
| 117 | 362 | 444 | 1.4 | 1000 | 0.00 | 6.29 |  |
| 118 | 356 | 440 | 2.2 | 1000 | 0.00 | --- |  |
| 119 | 359 | 441 | 1.2 | 1000 | 0.00 | 6.43 |  |
| 120 | 344 | 420 | 1 | 1000 | 0.00 | --- |  |
| 121 | 339 | 413 | 1 | 1000 | 0.00 | 6.39 |  |
| 122 | 380 | 465 | 1.1 | 1000 | 0.00 | --- |  |
| 125 | 363 | 441 | 1.3 | 1000 | 0.00 | 6.49 |  |
| 126 | 399 | 489 | 1.5 | 1000 | 0.00 | 6.54 |  |
| 127 | 393 | 486 | 1.5 | 1000 | 0.00 | --- |  |
| 128 | 407 | 503 | 1.7 | 1000 | 0.00 | 6.57 |  |
| 129 | 394 | 488 | 1.7 | 1000 | 0.00 | --- |  |
| 132 | 404 | 497 | 2.1 | 1000 | 0.00 | 6.56 |  |
| 134 | --- | --- | --- | --- | --- | 6.64 |  |
| 135 | 316 | 393 | 3.2 | 1000 | 0.00 | --- |  |
| 136 | 315 | 392 | 4.1 | 1000 | 0.00 | 6.62 |  |
| 137 | 312 | 388 | 5.3 | 1000 | 4.46 | --- |  |
| 138 | 309 | 386 | 6.2 | 1000 | 4.46 | --- |  |
| 139 | 305 | 384 | 7.3 | 1000 | 4.46 | 6.56 |  |
| 140 | 301 | 382 | 8 | 1000 | 4.46 | --- |  |
| 141 | 298 | 379 | 9.6 | 1000 | 4.46 | --- |  |
| 142 | 292 | 376 | 11.3 | 1000 | 4.46 | 6.44 |  |
| 143 | 288 | 374 | 13 | 1000 | 4.46 | --- |  |
| 145 | 278 | 368 | 18.1 | 1000 | 8.93 | --- |  |
| 146 | 271 | 364 | 20.1 | 1000 | 8.93 | 6.27 |  |
| 147 | 267 | 363 | 22 | 1000 | 8.93 | --- |  |
| 148 | 264 | 362 | 24.6 | 1000 | 8.93 | --- |  |
| 149 | 257 | 359 | 27.5 | 1000 | 13.39 | 6.02 |  |
| 150 | 267 | 357 | 15.5 | 560 | 13.39 | --- |  |
| 152 | 257 | 353 | 19.8 | 560 | 17.86 | --- |  |
| 153 | 254 | 350 | 21.4 | 560 | 17.86 | 5.73 |  |
| 154 | --- | --- | --- | --- | --- | --- |  |
| 155 | 250 | 340 | 22.3 | 560 | 17.86 | --- |  |
| 156 | 251 | 346 | 22.8 | 560 | 17.86 | --- |  |
| 157 | 250 | 346 | 23.0 | 560 | 17.86 | --- |  |
| 158 | --- | --- | --- | --- | --- | --- |  |
| 159 | --- | --- | --- | --- | --- | --- |  |
| 160 | 249 | 340 | 22.5 | 560 | 17.86 | --- |  |
| 161 | 245 | 340 | 24.0 | 560 | 17.86 | --- |  |
| 162 | 240 | 332 | 25.4 | 560 | 22.32 | --- |  |
| 163 | 235 | 338 | 23.7 | 560 | 17.86 | 3.20 | Addition of 5 mL SCN^-^ stock solution |
| 164 | 245 | 335 | 22.5 | 560 | 17.86 | --- |  |
| 165 | --- | --- | --- | --- | --- | --- |  |
| 166 | --- | --- | --- | --- | --- | --- |  |
| 167 | 245 | 330 | 21.6 | 560 | 17.86 | --- |  |
| 168 | 267 | 264 | 20.2 | 560 | 17.86 | --- |  |
| 169 | 252 | 326 | 19.1 | 560 | 13.39 | --- |  |
| 170 | 251 | --- | 18.4 | 560 | 13.39 | --- |  |
| 171 | 253 | 326 | 17.8 | 560 | 13.39 | --- |  |
| 172 | --- | --- | --- | 560 | --- | --- |  |
| 173 | --- | --- | --- | 560 | --- | --- |  |
| 174 | 251 | 327 | 16.5 | 560 | 13.39 | --- |  |
| 175 | 251 | 325 | 15.9 | 560 | 13.39 | 1.40 |  |
| 176 | 273 | 419 | 35.3 | 560 | 26.79 | --- |  |
| 177 | 290 | 420 | 20.4 | 560 | 17.86 | --- |  |
| 178 | 284 | 414 | 21.4 | 560 | 17.86 | --- |  |
| 179 | --- | --- | --- | --- | --- | --- |  |
| 180 | --- | --- | --- | --- | --- | --- |  |
| 181 | 282 | 405 | 18.8 | 560 | 13.39 | --- |  |
| 182 | 283 | 403 | 18.0 | 560 | 13.39 | 0.70 |  |
| 183 | --- | --- | --- | --- | --- | 5.20 | Medium replenishment and re-inoculation from previous culture |
| 184 | 264 | 403 | 13.0 | 560 | 8.93 | --- |  |
| 185 | 269 | 402 | 10.7 | 560 | 8.93 | --- |  |
| 186 | --- | --- | --- | --- | --- | --- |  |
| 187 | --- | --- | --- | --- | --- | --- |  |
| 188 | --- | --- | --- | --- | --- | --- |  |
| 189 | 262 | 393 | 14.1 | 560 | 13.39 | --- |  |
| 190 | 262 | 392 | 14.4 | 560 | 13.39 | --- |  |
| 191 | 259 | 389 | 15.9 | 560 | 13.39 | --- |  |
| 192 | 257 | 388 | 17.0 | 560 | 13.39 | 2.90 |  |
| 193 | --- | --- | --- | --- | --- | --- |  |
| 194 | --- | --- | --- | --- | --- | --- |  |
| 195 | 255 | 383 | 18.3 | 560 | 13.39 | --- |  |
| 196 | 255 | 381 | 17.8 | 560 | 13.39 | --- |  |
| 197 | 260 | 381 | 16.0 | 560 | 13.39 | 1.20 | Addition of 5 mL SCN^-^ stock solution |
| 198 | 262 | 377 | 16.7 | 560 | 13.39 | 4.40 |  |
| 199 | 268 | 377 | 13.9 | 560 | 8.93 | --- |  |
| 200 | --- | --- | --- | --- | --- | --- |  |
| 201 | --- | --- | --- | --- | --- | --- |  |
| 202 | 278 | 377 | 8.2 | 560 | 4.46 | --- | Addition of 5 mL SCN^-^ stock solution |
| 203 | 282 | 369 | 7.6 | 560 | 4.46 | --- |  |
| 204 | 284 | 373 | 8.2 | 560 | 4.46 | --- |  |
| 205 | 285 | 361 | 6.4 | 560 | 4.46 | --- |  |
| 206 | 286 | 375 | 6.1 | 560 | 4.46 | 7.40 |  |
| 207 | --- | --- | --- | --- | --- | --- |  |
| 208 | --- | --- | --- | --- | --- | --- |  |
| 209 | 284 | 373 | 6.2 | 560 | 4.46 | --- |  |
| 210 | 284 | 373 | 6.1 | 560 | 4.46 | --- |  |
| 211 | 285 | 372 | 6.6 | 560 | 4.46 | --- |  |
| 212 | 286 | 375 | 5.9 | 560 | 4.46 | --- |  |
| 213 | 285 | 372 | 6.0 | 560 | 4.46 | --- |  |
| 214 | --- | --- | --- | --- | --- | --- |  |
| 215 | --- | --- | --- | --- | --- | --- |  |
| 216 | 284 | 371 | 6.5 | 560 | 4.46 | --- |  |
| 217 | 283 | 370 | 6.5 | 560 | 4.46 | 7.30 |  |
| 218 | 283 | 369 | 6.6 | 560 | 4.46 | --- |  |
| 219 | 284 | 370 | 6.3 | 560 | 4.46 | --- |  |
| 220 | 284 | 370 | 5.9 | 560 | 4.46 | --- |  |
| 221 | --- | --- | --- | --- | --- | --- |  |
| 222 | --- | --- | --- | --- | --- | --- |  |
| 223 | 283 | 369 | 7.2 | 560 | 4.46 | --- |  |
| 224 | 285 | 369 | 6.2 | 560 | 4.46 | --- |  |
| 225 | 285 | 369 | 5.9 | 560 | 4.46 | 7.20 |  |
| 226 | --- | --- | --- | --- | --- | --- |  |
| 227 | --- | --- | --- | --- | --- | --- |  |
| 228 | --- | --- | --- | --- | --- | --- |  |
| 229 | --- | --- | --- | --- | --- | --- |  |
| 230 | 291 | 380 | 19.1 | 560 | 13.39 | --- | Medium replenishment. re-inoculation from previous culture. and DNA extraction |
| 231 | 313 | 401 | 3.3 | 560 | 4.46 | --- |  |
| 232 | --- | --- | --- | --- | --- | --- |  |
| 233 | --- | --- | --- | --- | --- | --- |  |
| 234 | 313 | 356 | 1.6 | 560 | 0.00 | --- |  |
| 235 | --- | --- | --- | --- | --- | --- |  |
| 236 | --- | --- | --- | --- | --- | --- |  |
| 237 | 308 | 407 | 2.1 | 560 | 0.00 | --- |  |
| 238 | 304 | 397 | 2.3 | 560 | 0.00 | --- |  |
| 239 | 307 | 403 | 2.6 | 560 | 0.00 | 2.40 |  |
| 240 | 305 | 396 | 2.8 | 560 | 4.46 | --- |  |
| 241 | --- | --- | --- | --- | --- | --- |  |
| 242 | --- | --- | --- | --- | --- | --- |  |
| 243 | --- | --- | --- | --- | --- | --- |  |
| 244 | 282 | 392 | 3.5 | 560 | 4.46 | --- |  |
| 245 | 300 | 389 | 4.0 | 560 | 4.46 | 1.30 |  |
| 246 | 298 | 388 | 4.6 | 560 | 4.46 | --- |  |
| 247 | 295 | 385 | 5.3 | 560 | 4.46 | --- |  |
| 248 | 292 | 382 | 6.2 | 560 | 4.46 | --- |  |
| 249 | --- | --- | --- | --- | --- | --- |  |
| 250 | --- | --- | --- | --- | --- | --- |  |
| 251 | 291 | 376 | 5.1 | 560 | 4.46 | 0.60 |  |
| 252 | 294 | 374 | 5.0 | 560 | 4.46 | 3.00 | Addition of 5 mL SCN^-^ stock solution |
| 253 | 295 | 369 | 5.8 | 560 | 4.46 | --- |  |
| 254 | 314 | 383 | 3.1 | 560 | 4.46 | 2.70 |  |
| 255 | 294 | 365 | 5.4 | 560 | 4.46 | 2.50 |  |
| 256 | --- | --- | --- | --- | --- | --- |  |
| 257 | --- | --- | --- | --- | --- | --- |  |
| 258 | 292 | 356 | 3.5 | 560 | 4.46 | --- |  |
| 259 | 293 | 356 | 4.4 | 560 | 4.46 | 2.00 | Addition of 3 mL SCN^-^ stock solution |
| 260 | 292 | 360 | 4.4 | 560 | 4.46 | 3.40 |  |
| 261 | 301 | 363 | 4.3 | 560 | 4.46 | --- |  |
| 262 | 296 | 362 | 4.1 | 560 | 4.46 | --- |  |
| 263 | --- | --- | --- | --- | --- | --- |  |
| 264 | --- | --- | --- | --- | --- | --- |  |
| 265 | 295 | 360 | 3.9 | 560 | 4.46 | 2.60 | Medium replenishment. re-inoculation from previous culture. and RNA extraction |
| 266 | 248 | 354 | 8.7 | 560 | 8.93 | --- | Addition of 5 mL SCN^-^ stock solution |
| 267 | 257 | 356 | 5.3 | 560 | 4.46 |  |  |
| 268 | 258 | 355 | 4.1 | 560 | 4.46 | 2.60 |  |
| 269 | 258 | 356 | 5.2 | 560 | 4.46 | --- |  |
| 270 | --- | --- | --- | --- | --- | --- |  |
| 271 | --- | --- | --- | --- | --- | --- |  |
| 272 | 253 | 351 | 8.8 | 560 | 8.93 | 1.70 |  |

**Supplemental File 3.** Electrochemical parameters of MFC B as well as actions taken over the entire experimental period.

| Days | Anode Potential (mV) | Cathode Potential (mV) | Cell Voltage (mV) | External resistance (Ω) | Current density (mA/m^2^) | SCN- (mM) | Comments |
| --- | --- | --- | --- | --- | --- | --- | --- |
| 1 |  |  |  |  | --- | 2.01 | Start of experiment with new medium, set-up and inoculation |
| 2 |  |  |  |  | --- | 2.40 |  |
| 8 |  |  |  |  | --- | 2.32 |  |
| 9 |  |  |  |  | --- | 2.37 |  |
| 16 |  |  |  |  | --- | 2.38 |  |
| 17 |  |  |  |  | --- | 2.37 |  |
| 20 |  |  |  |  | --- | 2.32 |  |
| 21 |  |  |  |  | --- | 2.32 |  |
| 22 |  |  |  |  | --- | 2.25 |  |
| 23 |  |  |  |  | --- | 2.23 |  |
| 24 |  |  |  |  | --- | 2.22 |  |
| 27 |  |  |  |  | --- | 2.23 |  |
| 28 |  |  |  |  | --- | 2.18 |  |
| 29 |  |  |  |  | --- | 2.15 |  |
| 30 |  |  |  |  | --- | 2.18 |  |
| 31 |  |  |  |  | --- |  |  |
| 34 |  |  |  |  | --- | 2.13 |  |
| 35 |  |  |  |  | --- |  |  |
| 36 |  |  |  |  | --- |  |  |
| 38 |  |  |  |  | --- | 2.23 |  |
| 44 |  |  |  |  | --- | 2.41 |  |
| 47 |  |  |  |  | --- |  |  |
| 48 |  |  |  |  | --- |  |  |
| 49 |  |  |  |  | --- | 2.39 |  |
| 50 |  |  |  |  | --- | 2.42 |  |
| 51 |  |  |  |  | --- | 2.42 |  |
| 52 |  |  |  |  | --- |  |  |
| 54 |  |  |  |  | --- |  |  |
| 55 |  |  |  |  | --- |  |  |
| 56 |  |  |  |  | --- | 2.43 |  |
| 57 |  |  |  |  | --- |  |  |
| 58 |  |  |  |  | --- |  |  |
| 58 |  |  |  |  | --- |  |  |
| 62 |  |  |  |  | --- | 2.49 | Nitrogen replacement delay |
| 63 |  |  |  |  | --- |  | Nitrogen supply restarted |
| 64 |  |  |  |  | --- |  |  |
| 93 |  |  |  |  | --- | 2.70 | Medium replenishment and re-inoculation from back-up culture |
| 94 |  |  |  |  | --- |  |  |
| 95 | 310 | 401 | - | 1000 | --- |  |  |
| 96 | 317 | 403 | - | 1000 | --- |  |  |
| 97 | 311 | 393 | - | 1000 | --- |  | Started nitrogen supply |
| 98 | 324 | 410 | - | 1000 | --- |  |  |
| 99 | 308 | 389 | - | 1000 | --- | 6.28 | Medium replenishment and re-inoculation from back-up culture |
| 100 |  |  |  |  | --- |  |  |
| 101 | 327 | 414 | - | 1000 | --- | 6.32 |  |
| 102 |  |  |  |  | --- |  |  |
| 103 |  |  |  |  | --- | 6.49 |  |
| 104 | 332 | 419 | - | 1000 | --- |  |  |
| 105 | 334 | 421 | - | 1000 | --- | 6.30 |  |
| 106 | 334 | 421 | - | 1000 | --- |  | Constant nitrogen supply restarted |
| 107 | 309 | 389 | - | 1000 | --- |  |  |
| 108 | 319 | 400 | - | 1000 | --- | 6.39 |  |
| 109 | 326 | 421 | - | 1000 | --- |  |  |
| 110 | 334 | 420 | - | 1000 | --- |  |  |
| 111 | 318 | 398 | - | 1000 | 0.00 |  | Re-inoculation from back-up culture |
| 112 | 322 | 403 | 0.10 | 1000 | 0.00 | 6.34 |  |
| 113 | 340 | 424 | - | 1000 | 0.00 |  |  |
| 114 | 362 | 451 | 0.10 | 1000 | 0.00 |  |  |
| 115 | 320 | 396 | - | 1000 | 0.00 |  |  |
| 116 |  |  |  |  | 0.00 |  |  |
| 117 | 332 | 412 | - | 1000 | 0.00 | 6.37 |  |
| 118 | 328 | 406 | 0.10 | 1000 | 0.00 |  |  |
| 119 |  |  |  |  | 0.00 | 6.49 |  |
| 120 | 372 | 460 | 0.10 | 1000 | 0.00 |  |  |
| 121 | 337 | 416 | 0.10 | 1000 | 0.00 | 6.34 |  |
| 122 | 344 | 424 | 0.10 | 1000 | 0.00 |  |  |
| 123 | 348 | 428 | 0.10 | 1000 | 0.00 |  | Medium replenishment, replacement of graphite felt and re-inoculation from back-up culture |
| 124 |  |  |  |  | --- |  |  |
| 125 |  |  |  |  | --- | 6.47 |  |
| 126 | 364 | 445 | 0 | 1000 | 0.00 | 6.70 |  |
| 127 | 475 | 586 | 0.1 | 1000 | 0.00 |  |  |
| 128 |  |  |  |  | --- | 6.68 |  |
| 129 | 401 | 493 | 1.7 | 1000 | 0.00 |  |  |
| 130 | 389 | 480 | 1.70 | 1000 | 0.00 |  |  |
| 131 |  |  |  |  | --- |  |  |
| 132 | 398 | 491 | 1.40 | 1000 | 0.00 | 6.66 |  |
| 133 |  |  |  |  | --- |  |  |
| 134 |  |  |  |  | --- | 6.62 |  |
| 135 | 306 | 376 | 1.10 | 1000 | 0.00 |  |  |
| 136 | 306 | 376 | 1.20 | 1000 | 0.00 | 6.71 |  |
| 137 | 305 | 375 | 1.30 | 1000 | 0.00 |  |  |
| 138 | 305 | 372 | 1.30 | 1000 | 0.00 |  |  |
| 139 | 304 | 372 | 1.40 | 1000 | 0.00 | 6.79 |  |
| 140 | 303 | 371 | 1 | 1000 | 0.00 |  |  |
| 141 | 304 | 371 | 1.5 | 1000 | 0.00 |  |  |
| 142 | 303 | 370 | 1.6 | 1000 | 0.00 |  |  |
| 143 | 303 | 369 | 1.7 | 1000 | 0.00 |  |  |
| 144 |  |  |  |  | --- |  |  |
| 145 | 303 | 368 | 1.7 | 1000 | 0.00 |  |  |
| 146 | 302 | 368 | 1.6 | 1000 | 0.00 |  |  |
| 147 | 302 | 367 | 1.6 | 1000 | 0.00 |  |  |
| 148 | 302 | 367 | 1.7 | 1000 | 0.00 |  |  |
| 149 |  |  |  |  | --- |  |  |
| 150 | 302 | 366 | 2 | 1000 | 0.00 |  |  |
| 151 |  |  |  |  | --- |  |  |
| 152 | 301 | 365 | 2.3 | 1000 | 0.00 |  |  |
| 153 |  |  |  |  | 0.00 |  |  |
| 154 | 302 | 365 | 2.3 | 1000 | 0.00 |  |  |
| 155 | 303 | 362 | 2.5 | 1000 | 0.00 |  |  |
| 156 | 305 | 365 | 2.6 | 1000 | 0.00 |  |  |
| 157 | 303 | 364 | 2.9 | 1000 | 0.00 |  |  |
| 158 |  |  |  |  |  |  |  |
| 159 |  |  |  |  |  |  |  |
| 160 | 305 | 363 | 2.5 | 1000 | 0.00 |  |  |
| 161 | 301 | 328 | 4.9 | 1000 | 0.00 |  |  |
| 162 | 299 | 361 | 6.3 | 1000 | 4.46 |  |  |
| 163 | 298 | 361 | 7.9 | 1000 | 4.46 | 4.50 |  |
| 164 | 292 | 359 | 9.5 | 1000 | 4.46 |  |  |
| 165 |  |  |  |  | --- |  |  |
| 166 |  |  |  |  | --- |  |  |
| 167 | 290 | 356 | 12.9 | 1000 | 4.46 |  |  |
| 168 | 288 | 355 | 13.7 | 1000 | 4.46 |  |  |
| 169 | 285 | 355 | 14.3 | 1000 | 4.46 |  |  |
| 170 | 286 | 357 | 14.4 | 1000 | 4.46 |  |  |
| 171 | 280 | 276 | 16.0 | 1000 | 8.93 |  |  |
| 172 |  |  |  |  |  |  |  |
| 173 |  |  |  |  |  |  |  |
| 174 | 273 | 350 | 17.6 | 1000 | 8.93 |  |  |
| 175 | 272 | 348 | 17.2 | 1000 | 8.93 | 2.80 | Nitrogen pumping stopped for about 5 hours |
| 176 | 265 | 426 | 58.1 | 1000 | 26.79 |  |  |
| 177 | 306 | 434 | 22.3 | 1000 | 8.93 |  |  |
| 178 | 303 | 431 | 22.1 | 1000 | 8.93 |  |  |
| 179 |  |  |  |  | --- |  |  |
| 180 |  |  |  |  | --- |  |  |
| 181 | 297 | 423 | 20.3 | 1000 | 8.93 |  |  |
| 182 | 302 | 420 | 12.0 | 560 | 8.93 | 1.50 |  |
| 183 |  |  |  |  | --- | 5.40 | Medium replenishment and re-inoculation from previous culture |
| 184 | 284 | 418 | 7.8 | 560 | 4.46 |  |  |
| 185 | 285 | 417 | 7.9 | 560 | 4.46 |  |  |
| 186 |  |  |  |  | --- |  |  |
| 187 |  |  |  |  | --- |  |  |
| 188 |  |  |  |  | --- |  |  |
| 189 | 282 | 403 | 9.7 | 560 | 8.93 |  |  |
| 190 | 282 | 406 | 9.8 | 560 | 8.93 |  |  |
| 191 | 279 | 403 | 12.5 | 560 | 8.93 |  |  |
| 192 | 275 | 400 | 13.8 | 560 | 8.93 | 2.30 |  |
| 193 |  |  |  |  | --- |  |  |
| 194 |  |  |  |  | --- |  |  |
| 195 | 272 | 394 | 16.3 | 560 | 13.39 |  |  |
| 196 | 273 | 392 | 15.0 | 560 | 13.39 |  |  |
| 197 | 291 | 396 | 3.3 | 560 | 4.46 | 0.00 | Addition of 5mL SCN^-^ stock solution |
| 198 | 267 | 388 | 22.3 | 560 | 17.86 | 2.60 |  |
| 199 | 270 | 387 | 19.2 | 560 | 13.39 |  |  |
| 200 |  |  |  |  | --- |  |  |
| 201 |  |  |  |  | --- |  |  |
| 202 | 278 | 386 | 12.8 | 560 | 8.93 |  | Addition of 5mL SCN^-^ stock solution |
| 203 | 281 | 384 | 12.1 | 560 | 8.93 |  |  |
| 204 | 280 | 383 | 13.1 | 560 | 8.93 |  |  |
| 205 | 278 | 383 | 14.7 | 560 | 13.39 |  |  |
| 206 | 278 | 381 | 15.2 | 560 | 13.39 | 4.20 |  |
| 207 |  |  |  |  | --- |  |  |
| 208 |  |  |  |  | --- |  |  |
| 209 | 273 | 378 | 17.2 | 560 | 13.39 |  |  |
| 210 | 271 | 376 | 17.0 | 560 | 13.39 |  |  |
| 211 | 274 | 376 | 16.8 | 560 | 13.39 |  |  |
| 212 | 278 | 378 | 13.6 | 560 | 8.93 |  |  |
| 213 | 276 | 375 | 14.9 | 560 | 13.39 |  |  |
| 214 |  |  |  |  | --- |  |  |
| 215 |  |  |  |  | --- |  |  |
| 216 | 277 | 373 | 12.9 | 560 | 8.93 |  |  |
| 217 | 286 | 373 | 5.4 | 560 | 4.46 | 0.10 | Addition of 5mL SCN^-^ stock solution |
| 218 | 275 | 371 | 16.2 | 560 | 13.39 |  |  |
| 219 | 277 | 371 | 13.6 | 560 | 8.93 |  |  |
| 220 | 279 | 371 | 12.8 | 560 | 8.93 |  |  |
| 221 |  |  |  |  | --- |  |  |
| 222 |  |  |  |  | --- |  |  |
| 223 | 281 | 370 | 12.4 | 560 | 8.93 |  |  |
| 224 | 278 | 369 | 14.3 | 560 | 13.39 |  |  |
| 225 | 282 | 369 | 11.4 | 560 | 8.93 | 0.20 |  |
| 226 |  |  |  |  | --- |  |  |
| 227 |  |  |  |  | --- |  |  |
| 228 |  |  |  |  | --- |  |  |
| 229 |  |  |  |  | --- |  |  |
| 230 | 305 | 404 | 13.9 | 560 | 8.93 |  | Medium replenishment, re-inoculation from previous culture, and DNA extraction |
| 231 | 324 | 419 | 1 | 560 | 0.00 |  |  |
| 232 |  |  |  |  | --- |  |  |
| 233 |  |  |  |  | --- |  |  |
| 234 | 322 | 421 | 1 | 560 | 0.00 |  |  |
| 235 |  |  |  |  | --- |  |  |
| 236 |  |  |  |  | --- |  |  |
| 237 | 319 | 412.0 | 2.0 | 560 | 0.00 |  |  |
| 238 | 317 | 409.0 | 2.0 | 560 | 0.00 |  |  |
| 239 | 314 | 406.0 | 2.4 | 560 | 0.00 | 1.40 |  |
| 240 | 313 | 403.0 | 2.6 | 560 | 0.00 |  |  |
| 241 |  |  |  |  | --- |  |  |
| 242 |  |  |  |  | --- |  |  |
| 243 |  |  |  |  | --- |  |  |
| 244 | 318 | 396.0 | 2.1 | 560 | 0.00 |  |  |
| 245 | 326 | 397.0 | 0.9 | 560 | 0.00 | 0.00 |  |
| 246 | 219 | 396.0 | 1.0 | 560 | 0.00 |  |  |
| 247 | 319 | 394.0 | 1.0 | 560 | 0.00 |  |  |
| 248 | 316 | 392.0 | 1.2 | 560 | 0.00 |  |  |
| 249 |  |  |  |  | --- |  |  |
| 250 |  |  |  |  | --- |  |  |
| 251 | 384 | 314.0 | 1.0 | 560 | 0.00 | 0.00 | Addition of 5mL SCN^-^ stock solution |
| 252 | 316 | 286.0 | 1.6 | 560 | 0.00 | 2.40 |  |
| 253 | 314 | 383.0 | 3.1 | 560 | 4.46 |  |  |
| 254 | 311 | 381.0 | 3.7 | 560 | 4.46 | 1.90 |  |
| 255 | 309 | 379.0 | 4.2 | 560 | 4.46 | 1.70 |  |
| 256 |  |  |  | 560 | 0.00 |  |  |
| 257 |  |  |  | 560 | 0.00 |  |  |
| 258 | 301 | 369.0 | 4.8 | 560 | 4.46 |  |  |
| 259 | 276 | 370.0 | 5.5 | 560 | 4.46 | 0.20 | Addition of 5mL SCN^-^ stock solution |
| 260 | 303 | 370.0 | 4.8 | 560 | 4.46 | 2.40 |  |
| 261 | 301 | 368.0 | 6.7 | 560 | 4.46 |  |  |
| 262 | 298 | 366.0 | 9.2 | 560 | 8.93 |  |  |
| 263 |  |  |  | 560 | 0.00 |  |  |
| 264 |  |  |  | 560 | 0.00 |  |  |
| 265 | 297 | 361.0 | 9.4 | 560 | 8.93 |  | Medium replenishment, re-inoculation from previous culture and RNA extraction |
| 266 | 295 | 360.0 | 7.1 | 560 | 4.46 |  | Addition of 5mL SCN^-^ stock solution |
| 267 | 297 | 359.0 | 8.0 | 560 | 4.46 |  |  |
| 268 | 301 | 356.0 | 8.6 | 560 | 8.93 | 2.00 |  |
| 269 | 299 | 356.0 | 9.6 | 560 | 8.93 |  |  |
| 270 |  |  |  |  | --- |  |  |
| 271 |  |  |  |  | --- |  |  |
| 272 | 298 | 352.0 | 8.8 | 560 | 8.93 | 0.40 | Addition of 5mL SCN^-^ stock solution |

**Supplemental File 4.** Calculation of Coulombic efficiency for MFCs A and B.

|  | MFC A | | MFC B | |
| --- | --- | --- | --- | --- |
| Days | Current (mA) | Charge (C) | Current (mA) | Charge (C) |
| 111 | 0.001 | 0.086 | 0.000 | 0.000 |
| 112 | 0.001 | 0.104 | 0.000 | 0.009 |
| 113 | 0.001 | 0.078 | 0.000 | 0.000 |
| 114 | 0.002 | 0.173 | 0.000 | 0.009 |
| 115 | 0.001 | 0.086 | 0.000 | 0.000 |
| 116 | 0.001 | 0.104 | 0.000 | 0.000 |
| 117 | 0.001 | 0.121 | 0.000 | 0.000 |
| 118 | 0.002 | 0.190 | 0.000 | 0.009 |
| 119 | 0.001 | 0.104 | 0.000 | 0.009 |
| 120 | 0.001 | 0.086 | 0.000 | 0.009 |
| 121 | 0.001 | 0.086 | 0.000 | 0.009 |
| 122 | 0.001 | 0.095 | 0.000 | 0.009 |
| 123 | 0.001 | 0.104 | 0.000 | 0.009 |
| 124 | 0.001 | 0.117 | 0.000 | 0.004 |
| 125 | 0.001 | 0.112 | 0.000 | 0.006 |
| 126 | 0.002 | 0.130 | 0.000 | 0.000 |
| 127 | 0.002 | 0.130 | 0.000 | 0.009 |
| 128 | 0.002 | 0.147 | 0.001 | 0.078 |
| 129 | 0.002 | 0.147 | 0.002 | 0.147 |
| 130 | 0.002 | 0.164 | 0.002 | 0.147 |
| 131 | 0.002 | 0.207 | 0.002 | 0.134 |
| 132 | 0.002 | 0.181 | 0.001 | 0.121 |
| 133 | 0.003 | 0.251 | 0.002 | 0.130 |
| 134 | 0.004 | 0.320 | 0.002 | 0.138 |
| 135 | 0.003 | 0.276 | 0.001 | 0.095 |
| 136 | 0.004 | 0.354 | 0.001 | 0.104 |
| 137 | 0.005 | 0.458 | 0.001 | 0.112 |
| 138 | 0.006 | 0.536 | 0.001 | 0.112 |
| 139 | 0.007 | 0.631 | 0.001 | 0.121 |
| 140 | 0.008 | 0.691 | 0.001 | 0.127 |
| 141 | 0.010 | 0.829 | 0.002 | 0.130 |
| 142 | 0.011 | 0.976 | 0.002 | 0.138 |
| 143 | 0.013 | 1.123 | 0.002 | 0.147 |
| 144 | 0.016 | 1.344 | 0.002 | 0.147 |
| 145 | 0.018 | 1.564 | 0.002 | 0.147 |
| 146 | 0.020 | 1.737 | 0.002 | 0.138 |
| 147 | 0.022 | 1.901 | 0.002 | 0.138 |
| 148 | 0.025 | 2.125 | 0.002 | 0.147 |
| 149 | 0.028 | 2.376 | 0.002 | 0.160 |
| 150 | 0.028 | 2.391 | 0.002 | 0.173 |
| 151 | 0.032 | 2.723 | 0.002 | 0.186 |
| 152 | 0.035 | 3.055 | 0.002 | 0.199 |
| 153 | 0.038 | 3.302 | 0.002 | 0.199 |
| 154 | 0.039 | 3.371 | 0.002 | 0.199 |
| 155 | 0.040 | 3.441 | 0.003 | 0.216 |
| 156 | 0.041 | 3.518 | 0.003 | 0.225 |
| 157 | 0.041 | 3.549 | 0.003 | 0.251 |
| 158 | 0.041 | 3.510 | 0.003 | 0.233 |
| 159 | 0.042 | 3.606 | 0.003 | 0.216 |
| 160 | 0.040 | 3.471 | 0.003 | 0.216 |
| 161 | 0.043 | 3.703 | 0.005 | 0.423 |
| 162 | 0.045 | 3.919 | 0.006 | 0.544 |
| 163 | 0.042 | 3.657 | 0.008 | 0.683 |
| 164 | 0.040 | 3.471 | 0.010 | 0.821 |
| 165 | 0.039 | 3.402 | 0.011 | 0.968 |
| 166 | 0.038 | 3.259 | 0.012 | 1.076 |
| 167 | 0.039 | 3.333 | 0.013 | 1.115 |
| 168 | 0.036 | 3.117 | 0.014 | 1.184 |
| 169 | 0.034 | 2.947 | 0.014 | 1.236 |
| 170 | 0.033 | 2.839 | 0.014 | 1.244 |
| 171 | 0.032 | 2.746 | 0.016 | 1.382 |
| 172 | 0.031 | 2.646 | 0.017 | 1.452 |
| 173 | 0.030 | 2.550 | 0.017 | 1.469 |
| 174 | 0.029 | 2.546 | 0.018 | 1.521 |
| 175 | 0.028 | 2.453 | 0.017 | 1.486 |
| 176 | 0.063 | 5.446 | 0.058 | 5.020 |
| 177 | 0.036 | 3.147 | 0.022 | 1.927 |
| 178 | 0.038 | 3.302 | 0.022 | 1.909 |
| 179 | 0.036 | 3.101 | 0.021 | 1.832 |
| 180 | 0.034 | 2.939 | 0.021 | 1.842 |
| 181 | 0.034 | 2.901 | 0.020 | 1.754 |
| 182 | 0.032 | 2.777 | 0.021 | 1.851 |
| 183 | 0.028 | 2.391 | 0.018 | 1.527 |
| 184 | 0.023 | 2.006 | 0.014 | 1.203 |
| 185 | 0.019 | 1.651 | 0.014 | 1.219 |
| 186 | 0.022 | 1.913 | 0.016 | 1.358 |
| 187 | 0.022 | 1.913 | 0.017 | 1.435 |
| 188 | 0.022 | 1.913 | 0.019 | 1.682 |
| 189 | 0.025 | 2.175 | 0.017 | 1.497 |
| 190 | 0.026 | 2.222 | 0.018 | 1.512 |
| 191 | 0.028 | 2.453 | 0.022 | 1.929 |
| 192 | 0.030 | 2.623 | 0.025 | 2.129 |
| 193 | 0.032 | 2.723 | 0.027 | 2.322 |
| 194 | 0.032 | 2.723 | 0.027 | 2.318 |
| 195 | 0.033 | 2.823 | 0.029 | 2.515 |
| 196 | 0.032 | 2.746 | 0.027 | 2.314 |
| 197 | 0.029 | 2.469 | 0.006 | 0.509 |
| 198 | 0.030 | 2.577 | 0.040 | 3.441 |
| 199 | 0.025 | 2.145 | 0.034 | 2.962 |
| 200 | 0.020 | 1.705 | 0.029 | 2.469 |
| 201 | 0.020 | 1.705 | 0.025 | 2.168 |
| 202 | 0.015 | 1.265 | 0.023 | 1.975 |
| 203 | 0.014 | 1.173 | 0.022 | 1.867 |
| 204 | 0.015 | 1.265 | 0.023 | 2.021 |
| 205 | 0.011 | 0.987 | 0.026 | 2.268 |
| 206 | 0.011 | 0.941 | 0.027 | 2.345 |
| 207 | 0.011 | 0.949 | 0.031 | 2.654 |
| 208 | 0.011 | 0.949 | 0.031 | 2.654 |
| 209 | 0.011 | 0.957 | 0.031 | 2.654 |
| 210 | 0.011 | 0.941 | 0.030 | 2.623 |
| 211 | 0.012 | 1.018 | 0.030 | 2.592 |
| 212 | 0.011 | 0.910 | 0.024 | 2.098 |
| 213 | 0.011 | 0.926 | 0.027 | 2.299 |
| 214 | 0.011 | 0.964 | 0.025 | 2.145 |
| 215 | 0.011 | 0.964 | 0.017 | 1.489 |
| 216 | 0.012 | 1.003 | 0.023 | 1.990 |
| 217 | 0.012 | 1.003 | 0.010 | 0.833 |
| 218 | 0.012 | 1.018 | 0.029 | 2.499 |
| 219 | 0.011 | 0.972 | 0.024 | 2.098 |
| 220 | 0.011 | 0.910 | 0.023 | 1.975 |
| 221 | 0.012 | 1.011 | 0.023 | 1.944 |
| 222 | 0.012 | 1.011 | 0.024 | 2.075 |
| 223 | 0.013 | 1.111 | 0.022 | 1.913 |
| 224 | 0.011 | 0.957 | 0.026 | 2.206 |
| 225 | 0.011 | 0.910 | 0.020 | 1.759 |
| 226 | 0.022 | 1.929 | 0.023 | 1.952 |
| 227 | 0.022 | 1.929 | 0.012 | 1.053 |
| 228 | 0.022 | 1.929 | 0.007 | 0.604 |
| 229 | 0.022 | 1.929 | 0.005 | 0.398 |
| 230 | 0.034 | 2.947 | 0.025 | 2.145 |
| 231 | 0.006 | 0.509 | 0.002 | 0.154 |
| 232 | 0.004 | 0.378 | 0.002 | 0.154 |
| 233 | 0.004 | 0.332 | 0.002 | 0.193 |
| 234 | 0.003 | 0.247 | 0.002 | 0.154 |
| 235 | 0.003 | 0.285 | 0.003 | 0.231 |
| 236 | 0.004 | 0.320 | 0.003 | 0.270 |
| 237 | 0.004 | 0.324 | 0.004 | 0.309 |
| 238 | 0.004 | 0.355 | 0.004 | 0.309 |
| 239 | 0.005 | 0.401 | 0.004 | 0.370 |
| 240 | 0.005 | 0.432 | 0.005 | 0.401 |
| 241 | 0.006 | 0.486 | 0.004 | 0.363 |
| 242 | 0.006 | 0.486 | 0.003 | 0.251 |
| 243 | 0.006 | 0.486 | 0.002 | 0.203 |
| 244 | 0.006 | 0.540 | 0.004 | 0.324 |
| 245 | 0.007 | 0.617 | 0.002 | 0.139 |
| 246 | 0.008 | 0.710 | 0.002 | 0.154 |
| 247 | 0.009 | 0.818 | 0.002 | 0.154 |
| 248 | 0.011 | 0.957 | 0.002 | 0.185 |
| 249 | 0.010 | 0.872 | 0.002 | 0.170 |
| 250 | 0.010 | 0.872 | 0.002 | 0.208 |
| 251 | 0.009 | 0.787 | 0.002 | 0.154 |
| 252 | 0.009 | 0.771 | 0.003 | 0.247 |
| 253 | 0.010 | 0.895 | 0.006 | 0.478 |
| 254 | 0.006 | 0.478 | 0.007 | 0.571 |
| 255 | 0.010 | 0.833 | 0.008 | 0.648 |
| 256 | 0.008 | 0.687 | 0.000 | 0.000 |
| 257 | 0.008 | 0.687 | 0.000 | 0.000 |
| 258 | 0.006 | 0.540 | 0.009 | 0.741 |
| 259 | 0.008 | 0.679 | 0.010 | 0.849 |
| 260 | 0.008 | 0.679 | 0.009 | 0.741 |
| 261 | 0.008 | 0.663 | 0.012 | 1.034 |
| 262 | 0.007 | 0.633 | 0.016 | 1.419 |
| 263 | 0.007 | 0.617 | 0.000 | 0.000 |
| 264 | 0.007 | 0.617 | 0.000 | 0.000 |
| 265 | 0.007 | 0.602 | 0.017 | 1.450 |
| 266 | 0.016 | 1.342 | 0.013 | 1.095 |
| 267 | 0.009 | 0.818 | 0.014 | 1.234 |
| 268 | 0.007 | 0.633 | 0.015 | 1.327 |
| 269 | 0.009 | 0.802 | 0.017 | 1.481 |
| 270 | 0.013 | 1.080 | 0.016 | 1.419 |
| 271 | 0.013 | 1.080 | 0.016 | 1.419 |
| 272 | 0.016 | 1.358 | 0.016 | 1.358 |

| Additions of SCN^-^ in MFC A | Initial (mM) | Final (mM) | Delta (mM) |
| --- | --- | --- | --- |
| day 111 - 153 | 1.0 | 0.9 | 0.1 |
| day 163 - 182 | 3.2 | 0.7 | 2.5 |
| day 183 - 197 | 5.2 | 1.2 | 4.0 |
| day 198 - 251 | 10.0 | 0.6 | 9.4 |
| day 252 - 259 | 3.0 | 2.0 | 1.0 |
| day 260 - 266 | 3.4 | 0.4 | 3.0 |
| day 268 - 272 | 2.6 | 1.7 | 0.9 |
| Additions of SCN^-^ in MFC B |  | | |
| day 112 - 182 | 6.3 | 1.5 | 4.8 |
| day 183 - 197 | 5.4 | 0.0 | 5.4 |
| day 198 - 216 | 10.0 | 0.1 | 9.9 |
| day 217 - 225 | 5.0 | 0.2 | 4.8 |
| day 230 - 251 | 5.0 | 0.0 | 5.0 |
| day 260 - 266 | 2.4 | 0.1 | 2.3 |
| day 267 - 272 | 5.0 | 0.4 | 4.6 |

|  | MFC A | MFC B |
| --- | --- | --- |
| Total decrease of SCN^-^ (mM) | 20.9 | 36.8 |
| Estimated anolyte volume (L) | 0.567 | |
| Faraday constant (C/mol) | 96485 | |
| n | 8 | |
| Charge from sulfate production from thiocyanate (C) | 9150.23 | 14938.20 |
| Estimated charge from current (C) | 231.74 | 153,99 |
| Coulombic efficiency (from day 111 to day 272) | 2.53% | 0.95% |

**Supplemental File 5.** General metatranscriptome data

| Sample ID | N. of read pairs (M) | N. of trimmed read pairs (M) | N. of 16S rRNA read pairs (M) | N. of mRNA read pairs (M) | Percentage of mRNA reads that could be assigned to a protein (%) |
| --- | --- | --- | --- | --- | --- |
| MFC A anode | 50.6 | 41.3 | 5.2 | 0.5 | 91.0 |
| MFC A anolyte | 42.8 | 35.7 | 3.0 | 0.6 | 88.9 |
| MFC B anode | 38.1 | 31.1 | 2.7 | 0.6 | 90.3 |
| MFC B anolyte | 38.1 | 24.9 | 0.6 | 2.7 | 83.2 |

**Supplemental File 6.** Number of mRNA counts in the duplicate MFCs and their taxonomic affiliations for selected genes relevant for thiocyanate degradation, sulfur metabolism, extracellular electron transport, ammonium assimilation, carbon dioxide fixation, cold adaptation, as well as the False Discovery Rate (FDR) from the differential expression analysis between the anode and anolyte community.

| **Taxa** | **MFC A**  **anode** | **MFC B**  **anode** | **MFC A**  **anolyte** | **MFC B anolyte** | **FDR** |
| --- | --- | --- | --- | --- | --- |
| **Thiocyanate and inorganic sulfur compound metabolism** |  |  |  |  |  |
|  |  |  |  |  |  |
| **Thiocyanate hydrolase *scn*** |  |  |  |  | 1 |
| Bacteria; | 67 | 4 | 0 | 47 |  |
| Bacteria;Proteobacteria; | 53 | 0 | 23 | 50 |  |
|  |  |  |  |  |  |
| **Cyanate hydratase/cyanase *cyn*** |  |  |  |  | 1 |
| Bacteria; | 0 | 47 | 6 | 44 |  |
| Bacteria;Actinobacteria;Actinobacteria; | 0 | 0 | 0 | 1 |  |
| Bacteria;Proteobacteria; | 10 | 284 | 9 | 182 |  |
| Bacteria;Proteobacteria;Alphaproteobacteria; | 3 | 0 | 23 | 0 |  |
| Bacteria;Proteobacteria;Alphaproteobacteria;Rhizobiales; | 3 | 0 | 0 | 0 |  |
| Bacteria;Proteobacteria;Betaproteobacteria; | 3 | 151 | 6 | 111 |  |
| Bacteria;Proteobacteria;Betaproteobacteria;Burkholderiales; | 0 | 0 | 0 | 2 |  |
| Bacteria;Proteobacteria;Betaproteobacteria;Burkholderiales;Oxalobacteraceae; | 0 | 4 | 0 | 3 |  |
| Bacteria;Proteobacteria;Betaproteobacteria;Burkholderiales;Oxalobacteraceae;Collimonas; | 0 | 0 | 0 | 4 |  |
| Bacteria;Proteobacteria;Betaproteobacteria;Burkholderiales;Oxalobacteraceae;Herbaspirillum | 0 | 0 | 0 | 2 |  |
| Bacteria;Proteobacteria;Betaproteobacteria;Neisseriales;Chromobacteriaceae;Chromobacterium group;Chromobacterium; | 0 | 7 | 6 | 5 |  |
| Bacteria;Proteobacteria;Betaproteobacteria;Nitrosomonadales;Thiobacillaceae;Thiobacillus;Thiobacillus sp. | 0 | 0 | 0 | 4 |  |
| Bacteria;Proteobacteria;Gammaproteobacteria; | 0 | 0 | 0 | 1 |  |
| Bacteria;Proteobacteria;Gammaproteobacteria;Pseudomonadales;Pseudomonadaceae;Pseudomonas; | 0 | 0 | 0 | 15 |  |
|  | 0 | 0 | 23 | 0 |  |
| **Sulfite reductase *dsr*** |  |  |  |  | 1 |
| Bacteria; | 5767 | 7294 | 5516 | 4387 |  |
| Bacteria;environmental samples;uncultured bacterium; | 0 | 7 | 0 | 3 |  |
| Bacteria;Proteobacteria; | 4212 | 3597 | 5914 | 2275 |  |
| Bacteria;Proteobacteria;Betaproteobacteria; | 3 | 0 | 6 | 4 |  |
| Bacteria;Proteobacteria;Betaproteobacteria;Nitrosomonadales; | 3 | 14 | 0 | 7 |  |
| Bacteria;Proteobacteria;Betaproteobacteria;Nitrosomonadales;Thiobacillaceae;Thiobacillus; | 0 | 14 | 3 | 35 |  |
| Bacteria;Proteobacteria;Gammaproteobacteria;Pseudomonadales;Pseudomonadaceae;Pseudomonas; | 0 | 7 | 0 | 1 |  |
| Bacteria;Proteobacteria;Gammaproteobacteria;unclassified Gammaproteobacteria;Candidatus Endoriftia; | 0 | 0 | 0 | 0 |  |
| Bacteria;Proteobacteria;Gammaproteobacteria;Xanthomonadales;Rhodanobacteraceae;Rhodanobacter; | 0 | 0 | 0 | 2 |  |
| Bacteria;Proteobacteria;Gammaproteobacteria;Xanthomonadales;Xanthomonadaceae; | 0 | 0 | 0 | 4 |  |
| Bacteria;Proteobacteria;Gammaproteobacteria;Xanthomonadales;Xanthomonadaceae;Pseudoxanthomonas; | 13 | 0 | 0 | 6 |  |
| Bacteria;Proteobacteria;Hydrogenophilalia;Hydrogenophilales;unclassified Hydrogenophilales; | 0 | 0 | 3 | 1 |  |
|  |  |  |  |  |  |
| **Adenylyl-sulfate reductase *sat*** |  |  |  |  | 1 |
| Bacteria; | 60 | 97 | 9 | 115 |  |
| Bacteria;Actinobacteria;Actinobacteria;Actinomycetales;Actinomycetaceae;Actinomyces; | 13 | 0 | 0 | 0 |  |
| Bacteria;Actinobacteria;Actinobacteria;Corynebacteriales;Nocardiaceae;Rhodococcus; | 13 | 29 | 0 | 0 |  |
| Bacteria;FCB group;Bacteroidetes/Chlorobi group;Bacteroidetes; | 0 | 4 | 0 | 0 |  |
| Bacteria;Proteobacteria; | 543 | 2961 | 529 | 4255 |  |
| Bacteria;Proteobacteria;Acidithiobacillia;Acidithiobacillales;unclassified Acidithiobacillales;Acidithiobacillales bacterium | 0 | 0 | 0 | 1 |  |
| Bacteria;Proteobacteria;Alphaproteobacteria; | 7 | 0 | 0 | 0 |  |
| Bacteria;Proteobacteria;Betaproteobacteria;Burkholderiales; | 0 | 0 | 0 | 3 |  |
| Bacteria;Proteobacteria;Betaproteobacteria;Burkholderiales;Burkholderiaceae; | 13 | 0 | 0 | 0 |  |
| Bacteria;Proteobacteria;Betaproteobacteria;Burkholderiales;Burkholderiaceae;Burkholderia; | 7 | 0 | 0 | 0 |  |
| Bacteria;Proteobacteria;Betaproteobacteria;Burkholderiales;Comamonadaceae; | 0 | 0 | 12 | 2 |  |
| Bacteria;Proteobacteria;Betaproteobacteria;Burkholderiales;Comamonadaceae;Acidovorax;Acidovorax sp. | 0 | 0 | 0 | 1 |  |
| Bacteria;Proteobacteria;Betaproteobacteria;Nitrosomonadales;Thiobacillaceae;Thiobacillus; | 0 | 0 | 0 | 10 |  |
| Bacteria;Proteobacteria;Gammaproteobacteria; | 0 | 0 | 0 | 3 |  |
| Bacteria;Proteobacteria;Gammaproteobacteria;Pseudomonadales;Moraxellaceae;Psychrobacter;Psychrobacter sp. | 0 | 0 | 0 | 1 |  |
| Bacteria;Proteobacteria;Gammaproteobacteria;Xanthomonadales; | 0 | 0 | 0 | 2 |  |
| Bacteria;Proteobacteria;Gammaproteobacteria;Xanthomonadales;Xanthomonadaceae; | 0 | 0 | 0 | 4 |  |
| Bacteria;Proteobacteria;Gammaproteobacteria;Xanthomonadales;Xanthomonadaceae;Pseudoxanthomonas; | 0 | 0 | 0 | 1 |  |
|  |  |  |  |  |  |
| **Adenylylsulphate reductase *apr*** |  |  |  |  | 0.65 |
| Bacteria; | 0 | 4 | 0 | 0 |  |
| Bacteria;FCB group;Bacteroidetes/Chlorobi group;Bacteroidetes; | 0 | 4 | 0 | 0 |  |
| Bacteria;FCB group;Bacteroidetes/Chlorobi group;Bacteroidetes;Sphingobacteriia;Sphingobacteriales;Sphingobacteriaceae; | 0 | 0 | 0 | 0 |  |
| Bacteria;Proteobacteria; | 0 | 0 | 0 | 2 |  |
| Bacteria;Proteobacteria;Gammaproteobacteria;Pseudomonadales;Pseudomonadaceae;Pseudomonas; | 27 | 0 | 0 | 0 |  |
| Bacteria;Proteobacteria;Gammaproteobacteria;Xanthomonadales;Xanthomonadaceae; | 0 | 0 | 0 | 1 |  |
| Bacteria;Proteobacteria;Gammaproteobacteria;Xanthomonadales;Xanthomonadaceae;Pseudoxanthomonas; | 0 | 0 | 0 | 1 |  |
|  |  |  |  |  |  |
| **Sulphurtransferase sulfur carrier accessory protein *tus*** |  |  |  |  | 0.97 |
| Bacteria; | 20 | 22 | 12 | 38 |  |
| Bacteria;Proteobacteria; | 649 | 471 | 375 | 754 |  |
| Bacteria;Proteobacteria;Betaproteobacteria; | 0 | 0 | 0 | 1 |  |
| Bacteria;Proteobacteria;Betaproteobacteria;Burkholderiales;Burkholderiaceae;Paraburkholderia;Paraburkholderia kururiensis; | 0 | 7 | 0 | 0 |  |
| Bacteria;Proteobacteria;Betaproteobacteria;Nitrosomonadales;Thiobacillaceae;Thiobacillus; | 3 | 0 | 0 | 7 |  |
| Bacteria;Proteobacteria;Gammaproteobacteria;Pseudomonadales;Pseudomonadaceae;Pseudomonas; | 13 | 0 | 0 | 0 |  |
| Bacteria;Proteobacteria;Hydrogenophilalia;Hydrogenophilales;unclassified Hydrogenophilales; | 0 | 0 | 0 | 1 |  |
|  |  |  |  |  |  |
| **Heterodisulfide reductase *hdr*** |  |  |  | N/A |  |
| Bacteria; | 3 | 0 | 0 | 0 |  |
| Bacteria;Proteobacteria;Gammaproteobacteria;Xanthomonadales;Rhodanobacteraceae; | 3 | 0 | 0 | 0 |  |
|  |  |  |  |  |  |
| **Sox complex *soxAX*** |  |  |  |  | 1 |
| Bacteria; | 17 | 36 | 0 | 0 |  |
| Bacteria;Proteobacteria; | 1325 | 1430 | 1212 | 1127 |  |
| Bacteria;Proteobacteria;Alphaproteobacteria; | 13 | 0 | 0 | 0 |  |
| Bacteria;Proteobacteria;Alphaproteobacteria;Rhizobiales; | 37 | 29 | 0 | 3 |  |
| Bacteria;Proteobacteria;Betaproteobacteria; | 63 | 0 | 47 | 3 |  |
| Bacteria;Proteobacteria;Betaproteobacteria;Burkholderiales; | 0 | 0 | 6 | 0 |  |
| Bacteria;Proteobacteria;Betaproteobacteria;Burkholderiales;Comamonadaceae;Hydrogenophaga; | 3 | 0 | 6 | 0 |  |
| Bacteria;Proteobacteria;Betaproteobacteria;Nitrosomonadales;Thiobacillaceae;Thiobacillus; | 110 | 471 | 58 | 110 |  |
| Bacteria;Proteobacteria;Hydrogenophilalia;Hydrogenophilales;unclassified Hydrogenophilales; | 7 | 0 | 6 | 1 |  |
| Bacteria;Proteobacteria;Hydrogenophilalia;Hydrogenophilales;unclassified Hydrogenophilales;Hydrogenophilales bacterium | 3 | 0 | 0 | 0 |  |
|  |  |  |  |  |  |
| **Sox complex *soxX*** |  |  |  |  | 1 |
| Bacteria;Proteobacteria; | 1428 | 223 | 1323 | 290 |  |
| Bacteria;Proteobacteria;Alphaproteobacteria; | 7 | 0 | 0 | 0 |  |
| Bacteria;Proteobacteria;Alphaproteobacteria;Rhizobiales; | 143 | 14 | 6 | 0 |  |
| Bacteria;Proteobacteria;Alphaproteobacteria;Rhizobiales;unclassified Rhizobiales;unclassified Rhizobiales;Rhizobiales bacterium | 3 | 0 | 0 | 0 |  |
| Bacteria;Proteobacteria;Alphaproteobacteria;Rhizobiales;Xanthobacteraceae; | 7 | 0 | 0 | 0 |  |
| Bacteria;Proteobacteria;Alphaproteobacteria;Rhizobiales;Xanthobacteraceae;Xanthobacter; | 60 | 0 | 0 | 0 |  |
| Bacteria;Proteobacteria;Betaproteobacteria; | 123 | 0 | 12 | 2 |  |
| Bacteria;Proteobacteria;Betaproteobacteria;Burkholderiales; | 47 | 0 | 0 | 0 |  |
| Bacteria;Proteobacteria;Betaproteobacteria;Burkholderiales;Comamonadaceae; | 13 | 0 | 0 | 0 |  |
| Bacteria;Proteobacteria;Betaproteobacteria;Burkholderiales;Comamonadaceae;Hydrogenophaga; | 7 | 0 | 0 | 0 |  |
| Bacteria;Proteobacteria;Betaproteobacteria;Burkholderiales;Comamonadaceae;Polaromonas; | 7 | 0 | 0 | 0 |  |
| Bacteria;Proteobacteria;Betaproteobacteria;Burkholderiales;Comamonadaceae;Zhizhongheella;Zhizhongheella caldifontis; | 7 | 0 | 0 | 0 |  |
| Bacteria;Proteobacteria;Betaproteobacteria;Nitrosomonadales;Thiobacillaceae;Thiobacillus; | 220 | 597 | 125 | 71 |  |
| Bacteria;Proteobacteria;Hydrogenophilalia;Hydrogenophilales;unclassified Hydrogenophilales;Hydrogenophilales bacterium | 13 | 0 | 12 | 0 |  |
|  |  |  |  |  |  |
| **Sox complex *soxB*** |  |  |  |  | 1 |
| Bacteria; | 216 | 201 | 285 | 207 |  |
| Bacteria;Proteobacteria; | 180 | 187 | 238 | 296 |  |
| Bacteria;Proteobacteria;Alphaproteobacteria;Rhizobiales; | 40 | 11 | 0 | 0 |  |
| Bacteria;Proteobacteria;Alphaproteobacteria;Rhizobiales;Bradyrhizobiaceae; | 0 | 4 | 3 | 0 |  |
| Bacteria;Proteobacteria;Alphaproteobacteria;Rhizobiales;Xanthobacteraceae;Xanthobacter; | 7 | 0 | 0 | 0 |  |
| Bacteria;Proteobacteria;Betaproteobacteria; | 7 | 0 | 58 | 4 |  |
| Bacteria;Proteobacteria;Betaproteobacteria;Burkholderiales; | 0 | 0 | 0 | 1 |  |
| Bacteria;Proteobacteria;Betaproteobacteria;Burkholderiales;Comamonadaceae;Hydrogenophaga; | 0 | 0 | 12 | 0 |  |
| Bacteria;Proteobacteria;Betaproteobacteria;;unclassified Burkholderiales;Burkholderiales bacterium | 0 | 0 | 0 | 0 |  |
| Bacteria;Proteobacteria;Betaproteobacteria;Nitrosomonadales;Thiobacillaceae;Thiobacillus; | 0 | 57 | 0 | 20 |  |
| Bacteria;Proteobacteria;Betaproteobacteria;unclassified Betaproteobacteria;unclassified Betaproteobacteria;Betaproteobacteria sp. | 0 | 0 | 0 | 2 |  |
| Bacteria;Proteobacteria;Gammaproteobacteria;Xanthomonadales;Rhodanobacteraceae;Rhodanobacter;Rhodanobacter sp. | 3 | 0 | 0 | 0 |  |
| Bacteria;Proteobacteria;Hydrogenophilalia;Hydrogenophilales;unclassified Hydrogenophilales;Hydrogenophilales bacterium | 0 | 0 | 0 | 1 |  |
|  |  |  |  |  |  |
| **Sox complex *soxC*** |  |  |  |  | 1 |
| Bacteria; | 20 | 14 | 0 | 0 |  |
| Bacteria;Proteobacteria; | 60 | 75 | 3 | 39 |  |
| Bacteria;Proteobacteria;Alphaproteobacteria; | 17 | 0 | 0 | 1 |  |
| Bacteria;Proteobacteria;Alphaproteobacteria;Rhizobiales; | 50 | 0 | 0 | 4 |  |
| Bacteria;Proteobacteria;Alphaproteobacteria;Rhizobiales;Bradyrhizobiaceae; | 7 | 0 | 0 | 0 |  |
| Bacteria;Proteobacteria;Alphaproteobacteria;Rhizobiales;Methylobacteriaceae;Methylobacterium; | 6 | 0 | 0 | 0 |  |
| Bacteria;Proteobacteria;Betaproteobacteria; | 37 | 7 | 12 | 63 |  |
| Bacteria;Proteobacteria;Betaproteobacteria;Burkholderiales; | 0 | 0 | 0 | 4 |  |
| Bacteria;Proteobacteria;Betaproteobacteria;Nitrosomonadales;Thiobacillaceae;Thiobacillus; | 0 | 154 | 9 | 157 |  |
| Bacteria;Proteobacteria;Gammaproteobacteria;Xanthomonadales;Rhodanobacteraceae;Rhodanobacter; | 7 | 0 | 0 | 0 |  |
|  |  |  |  |  |  |
| **Sox complex *soxY*** |  |  |  |  | 1 |
| Bacteria; | 3 | 0 | 0 | 2 |  |
| Bacteria;Proteobacteria; | 1941 | 356 | 1329 | 424 |  |
| Bacteria;Proteobacteria;Alphaproteobacteria; | 3 | 0 | 0 | 0 |  |
| Bacteria;Proteobacteria;Alphaproteobacteria;Rhizobiales; | 70 | 4 | 6 | 1 |  |
| Bacteria;Proteobacteria;Alphaproteobacteria;Rhizobiales;Bradyrhizobiaceae; | 0 | 0 | 3 | 0 |  |
| Bacteria;Proteobacteria;Alphaproteobacteria;Rhizobiales;Xanthobacteraceae;Xanthobacter; | 20 | 0 | 0 | 0 |  |
| Bacteria;Proteobacteria;Alphaproteobacteria;Rhizobiales;Xanthobacteraceae;Xanthobacter;Xanthobacter autotrophicus; | 3 | 0 | 0 | 0 |  |
| Bacteria;Proteobacteria;Betaproteobacteria; | 43 | 0 | 108 | 1 |  |
| Bacteria;Proteobacteria;Betaproteobacteria;Burkholderiales; | 20 | 0 | 26 | 0 |  |
| Bacteria;Proteobacteria;Betaproteobacteria;Burkholderiales;Burkholderiaceae; | 0 | 0 | 6 | 0 |  |
| Bacteria;Proteobacteria;Betaproteobacteria;Burkholderiales;Burkholderiaceae;Ralstonia; | 0 | 0 | 6 | 0 |  |
| Bacteria;Proteobacteria;Betaproteobacteria;Burkholderiales;Comamonadaceae;Hydrogenophaga; | 27 | 0 | 90 | 0 |  |
| Bacteria;Proteobacteria;Betaproteobacteria;Burkholderiales;Comamonadaceae;Hydrogenophaga;Hydrogenophaga palleronii; | 0 | 0 | 3 | 0 |  |
| Bacteria;Proteobacteria;Betaproteobacteria;Nitrosomonadales;Gallionellaceae;Sulfuriferula;Sulfuriferula sp. AH1; | 3 | 0 | 0 | 0 |  |
| Bacteria;Proteobacteria;Betaproteobacteria;Nitrosomonadales;Thiobacillaceae;Thiobacillus; | 3 | 0 | 0 | 1 |  |
| Bacteria;Proteobacteria;Gammaproteobacteria; | 0 | 0 | 3 | 0 |  |
| Bacteria;Proteobacteria;Gammaproteobacteria;Xanthomonadales;Rhodanobacteraceae;Rhodanobacter; | 0 | 25 | 0 | 0 |  |
| Bacteria;Proteobacteria;Hydrogenophilalia;Hydrogenophilales;unclassified Hydrogenophilales; | 3 | 0 | 6 | 0 |  |
| Bacteria;Proteobacteria;Hydrogenophilalia;Hydrogenophilales;unclassified Hydrogenophilales;Hydrogenophilales bacterium | 150 | 14 | 148 | 7 |  |

| **Taxa** | **MFC A anode** | **MFC B anode** | **MFC A anolyte** | **MFC B anolyte** | **FDR** |
| --- | --- | --- | --- | --- | --- |
| **Extracellular electron transfer** |  |  |  |  |  |
|  |  |  |  |  |  |
| **Type II secretion *gspDEGHM*** |  |  |  |  | 0.88- 1 |
| Bacteria; | 40 | 93 | 6 | 118 |  |
| Bacteria;Actinobacteria;Actinobacteria; | 0 | 0 | 6 | 0 |  |
| Bacteria;Actinobacteria;Actinobacteria;Corynebacteriales; | 13 | 0 | 0 | 0 |  |
| Bacteria;Actinobacteria;Actinobacteria;Corynebacteriales;Nocardiaceae; | 7 | 0 | 6 | 0 |  |
| Bacteria;Actinobacteria;Actinobacteria;Corynebacteriales;Nocardiaceae;Rhodococcus; | 20 | 0 | 0 | 0 |  |
| Bacteria;Actinobacteria;Actinobacteria;Micrococcales;Microbacteriaceae; | 0 | 0 | 0 | 1 |  |
| Bacteria;Actinobacteria;Actinobacteria;Propionibacteriales;Propionibacteriaceae;Cutibacterium; | 39 | 0 | 0 | 0 |  |
| Bacteria;Proteobacteria; | 380 | 442 | 180 | 900 |  |
| Bacteria;Proteobacteria;Alphaproteobacteria;Caulobacterales; | 13 | 0 | 3 | 0 |  |
| Bacteria;Proteobacteria;Alphaproteobacteria;Sphingomonadales;Sphingomonadaceae;Sphingomonas; | 3 | 0 | 12 | 0 |  |
| Bacteria;Proteobacteria;Alphaproteobacteria;Rhizobiales;Bradyrhizobiaceae;Bradyrhizobium; | 0 | 0 | 0 | 2 |  |
| Bacteria;Proteobacteria;Betaproteobacteria; | 53 | 22 | 12 | 124 |  |
| Bacteria;Proteobacteria;Betaproteobacteria;Burkholderiales; | 20 | 43 | 35 | 19 |  |
| Bacteria;Proteobacteria;Betaproteobacteria;Burkholderiales;Alcaligenaceae;Bordetella;Bordetella | 17 | 0 | 0 | 6 |  |
| Bacteria;Proteobacteria;Betaproteobacteria;Burkholderiales;Burkholderiaceae; | 3 | 14 | 0 | 7 |  |
| Bacteria;Proteobacteria;Betaproteobacteria;Burkholderiales;Burkholderiaceae;Burkholderia; | 3 | 0 | 0 | 4 |  |
| Bacteria;Proteobacteria;Betaproteobacteria;Burkholderiales;Comamonadaceae; | 0 | 36 | 0 | 10 |  |
| Bacteria;Proteobacteria;Betaproteobacteria;Burkholderiales;Comamonadaceae;Acidovorax; | 0 | 0 | 0 | 5 |  |
| Bacteria;Proteobacteria;Betaproteobacteria;Burkholderiales;Comamonadaceae;Hydrogenophaga; | 40 | 0 | 0 | 3 |  |
| Bacteria;Proteobacteria;Betaproteobacteria;Burkholderiales;Comamonadaceae;Polaromonas; | 0 | 0 | 0 | 1 |  |
| Bacteria;Proteobacteria;Betaproteobacteria;Nitrosomonadales; | 0 | 0 | 0 | 15 |  |
| Bacteria;Proteobacteria;Betaproteobacteria;Nitrosomonadales;Thiobacillaceae;Thiobacillus; | 17 | 151 | 3 | 359 |  |
| Bacteria;Proteobacteria;Gammaproteobacteria; | 3 | 0 | 0 | 0 |  |
| Bacteria;Proteobacteria;Gammaproteobacteria;Enterobacterales;Enterobacteriaceae;Enterobacter; | 13 | 0 | 0 | 0 |  |
| Bacteria;Proteobacteria;Gammaproteobacteria;Pseudomonadales;Moraxellaceae;Acinetobacter;Acinetobacter | 14 | 0 | 0 | 0 |  |
| Bacteria;Proteobacteria;Gammaproteobacteria;Pseudomonadales;Pseudomonadaceae;Pseudomonas; | 3 | 14 | 0 | 2 |  |
| Bacteria;Proteobacteria;Gammaproteobacteria;Xanthomonadales; | 0 | 0 | 0 | 3 |  |
| Bacteria;Proteobacteria;Gammaproteobacteria;Xanthomonadales;Rhodanobacteraceae;Rhodanobacter; | 3 | 0 | 0 | 7 |  |
| Bacteria;Proteobacteria;Gammaproteobacteria;Xanthomonadales;Xanthomonadaceae; | 3 | 0 | 0 | 1 |  |
| Bacteria;Proteobacteria;Gammaproteobacteria;Xanthomonadales;Xanthomonadaceae;Pseudoxanthomonas; | 13 | 0 | 0 | 10 |  |
|  |  |  |  |  |  |
| **Fimbrial protein pilin** |  |  |  |  | 0.90 |
| Bacteria;Proteobacteria; | 110 | 43 | 15 | 34 |  |
| Bacteria;Proteobacteria;Betaproteobacteria; | 0 | 0 | 0 | 5 |  |
| Bacteria;Proteobacteria;Betaproteobacteria;Burkholderiales; | 13 | 7 | 3 | 6 |  |
| Bacteria;Proteobacteria;Betaproteobacteria;Burkholderiales;Comamonadaceae;Acidovorax;Acidovorax sp. | 0 | 7 | 3 | 1 |  |
| Bacteria;Proteobacteria;Betaproteobacteria;Burkholderiales;Oxalobacteraceae;Herminiimonas;Herminiimonas arsenicoxydans; | 0 | 0 | 0 | 2 |  |
| Bacteria;Proteobacteria;Betaproteobacteria;Rhodocyclales;Rhodocyclaceae;unclassified Rhodocyclaceae;Rhodocyclaceae sp. | 0 | 0 | 0 | 1 |  |
| Bacteria;Proteobacteria;Gammaproteobacteria; | 10 | 4 | 0 | 1 |  |
| Bacteria;Proteobacteria;Gammaproteobacteria;Methylococcales;Methylococcaceae;Methyloglobulus;Methyloglobulus morosus | 0 | 0 | 0 | 2 |  |
| Bacteria;Proteobacteria;Gammaproteobacteria;Pseudomonadales;Pseudomonadaceae;Pseudomonas;Pseudomonas syringae | 3 | 0 | 0 | 0 |  |
| Bacteria;Proteobacteria;Gammaproteobacteria;Xanthomonadales; | 17 | 0 | 0 | 1 |  |
| Bacteria;Proteobacteria;Gammaproteobacteria;Xanthomonadales;Rhodanobacteraceae; | 53 | 0 | 0 | 0 |  |
| Bacteria;Proteobacteria;Gammaproteobacteria;Xanthomonadales;Rhodanobacteraceae;Rhodanobacter; | 154 | 43 | 15 | 17 |  |
| Bacteria;Proteobacteria;Gammaproteobacteria;Xanthomonadales;Xanthomonadaceae; | 3 | 0 | 0 | 0 |  |
| Bacteria;Proteobacteria;Gammaproteobacteria;Xanthomonadales;Xanthomonadaceae;Pseudoxanthomonas; | 3 | 0 | 0 | 5 |  |
| Bacteria;Proteobacteria;Gammaproteobacteria;Xanthomonadales;Xanthomonadaceae;Pseudoxanthomonas sp. | 0 | 0 | 0 | 1 |  |
|  |  |  |  |  |  |
| **Pili assembly chaperone** |  |  |  |  | 0.69 |
| Bacteria;Actinobacteria;Actinobacteria;Propionibacteriales;Propionibacteriaceae; | 3 | 0 | 0 | 0 |  |
| Bacteria;Actinobacteria;Actinobacteria;Propionibacteriales;Propionibacteriaceae;Cutibacterium;Cutibacterium acnes; | 3 | 0 | 0 | 0 |  |
| Bacteria;Proteobacteria; | 0 | 4 | 0 | 0 |  |
| Bacteria;Proteobacteria;Betaproteobacteria;Burkholderiales;Burkholderiaceae;Ralstonia;Ralstonia insidiosa; | 0 | 4 | 0 | 0 |  |
| Bacteria;Proteobacteria;Gammaproteobacteria;Enterobacterales;Yersiniaceae; | 7 | 0 | 0 | 0 |  |
| Bacteria;Proteobacteria;Gammaproteobacteria;Pseudomonadales;Pseudomonadaceae;Pseudomonas; | 13 | 0 | 0 | 0 |  |
|  |  |  |  |  |  |
| **Menaquinone biosynthesis *menBC*** |  |  |  |  | 1 |
| Bacteria; | 34 | 0 | 29 | 7 |  |
| Bacteria;Actinobacteria; | 7 | 0 | 24 | 25 |  |
| Bacteria;Actinobacteria;Actinobacteria; | 3 | 0 | 0 | 1 |  |
| Bacteria;Actinobacteria;Actinobacteria;Corynebacteriales; | 7 | 0 | 0 | 0 |  |
| Bacteria;Actinobacteria;Actinobacteria;Corynebacteriales;Corynebacteriaceae;Corynebacterium; | 13 | 0 | 0 | 0 |  |
| Bacteria;Actinobacteria;Actinobacteria;Corynebacteriales;Mycobacteriaceae;Mycobacterium; | 0 | 0 | 0 | 6 |  |
| Bacteria;Actinobacteria;Actinobacteria;Corynebacteriales;unclassified Corynebacteriales;Lawsonella | 3 | 0 | 0 | 0 |  |
| Bacteria;Actinobacteria;Actinobacteria;Micrococcales; | 0 | 0 | 18 | 12 |  |
| Bacteria;Actinobacteria;Actinobacteria;Micrococcales;Microbacteriaceae; | 14 | 0 | 12 | 31 |  |
| Bacteria;Actinobacteria;Actinobacteria;Micrococcales;Microbacteriaceae;Cryobacterium;Cryobacterium mesophilum; | 0 | 0 | 0 | 3 |  |
| Bacteria;Actinobacteria;Actinobacteria;Micrococcales;Microbacteriaceae;Cryobacterium;Cryobacterium roopkundense; | 0 | 0 | 0 | 1 |  |
| Bacteria;Actinobacteria;Actinobacteria;Micrococcales;Microbacteriaceae;Leifsonia; | 0 | 0 | 6 | 0 |  |
| Bacteria;Actinobacteria;Actinobacteria;Micrococcales;Microbacteriaceae;Microbacterium;Microbacterium sp. | 0 | 0 | 0 | 1 |  |
| Bacteria;Actinobacteria;Actinobacteria;Micrococcales;Micrococcaceae;Rothia; | 0 | 14 | 0 | 0 |  |
| Bacteria;Actinobacteria;Actinobacteria;Propionibacteriales;Propionibacteriaceae; | 183 | 7 | 0 | 0 |  |
| Bacteria;Actinobacteria;Actinobacteria;Propionibacteriales;Propionibacteriaceae;Cutibacterium;Cutibacterium acnes; | 17 | 7 | 0 | 0 |  |
| Bacteria;Firmicutes;Bacilli;Bacillales;Bacillales incertae sedis;Bacillales Family XII. Incertae Sedis;Exiguobacterium; | 13 | 0 | 0 | 0 |  |
| Bacteria;Firmicutes;Bacilli;Bacillales;Staphylococcaceae;Staphylococcus; | 13 | 0 | 0 | 0 |  |
| Bacteria;FCB group;Bacteroidetes/Chlorobi group;Bacteroidetes; | 0 | 0 | 0 | 1 |  |
| Bacteria;FCB group;Bacteroidetes/Chlorobi group;Bacteroidetes;Sphingobacteriia;Sphingobacteriales;Sphingobacteriaceae | 13 | 0 | 0 | 7 |  |
| Bacteria;Proteobacteria; | 17 | 0 | 6 | 4 |  |
| Bacteria;Proteobacteria;Alphaproteobacteria;Sphingomonadales;Sphingomonadaceae;Sphingopyxis; | 0 | 0 | 18 | 0 |  |
| Bacteria;Proteobacteria;Gammaproteobacteria; | 0 | 8 | 0 | 0 |  |
| Bacteria;Proteobacteria;Gammaproteobacteria;Enterobacterales;Enterobacteriaceae; | 0 | 14 | 0 | 0 |  |
| Bacteria;Proteobacteria;Gammaproteobacteria;Pasteurellales;Pasteurellaceae;Haemophilus; | 0 | 8 | 0 | 0 |  |
| Bacteria;Proteobacteria;Gammaproteobacteria;Pseudomonadales;Pseudomonadaceae;Pseudomonas; | 13 | 0 | 0 | 0 |  |
|  |  |  |  |  |  |
| **Riboflavin biosynthesis protein *ribBAD*** |  |  |  |  | 0.97- 1 |
| Bacteria; | 20 | 11 | 12 | 32 |  |
| Bacteria;Actinobacteria; | 0 | 0 | 3 | 0 |  |
| Bacteria;Actinobacteria;Actinobacteria; | 0 | 0 | 6 | 5 |  |
| Bacteria;Actinobacteria;Actinobacteria;Corynebacteriales;Corynebacteriaceae;Corynebacterium; | 27 | 0 | 0 | 0 |  |
| Bacteria;Actinobacteria;Actinobacteria;Corynebacteriales;unclassified Corynebacteriales;Lawsonella | 13 | 0 | 0 | 0 |  |
| Bacteria;Actinobacteria;Actinobacteria;Micrococcales; | 0 | 0 | 0 | 3 |  |
| Bacteria;Actinobacteria;Actinobacteria;Micrococcales;Microbacteriaceae; | 0 | 0 | 0 | 8 |  |
| Bacteria;Actinobacteria;Actinobacteria;Propionibacteriales;Propionibacteriaceae; | 117 | 0 | 0 | 2 |  |
| Bacteria;Actinobacteria;Actinobacteria;Propionibacteriales;Propionibacteriaceae;Cutibacterium; | 3 | 0 | 0 | 0 |  |
| Bacteria;Bacteroidetes/Chlorobi group;Bacteroidetes; | 7 | 0 | 6 | 69 |  |
| Bacteria;Bacteroidetes/Chlorobi group;Bacteroidetes;Sphingobacteriia; | 0 | 0 | 0 | 1 |  |
| Bacteria;Bacteroidetes/Chlorobi group;Bacteroidetes;Sphingobacteriia;Sphingobacteriales; | 0 | 0 | 0 | 1 |  |
| Bacteria;Bacteroidetes/Chlorobi group;Bacteroidetes;Sphingobacteriia;Sphingobacteriales;Sphingobacteriaceae | 7 | 47 | 6 | 230 |  |
| Bacteria;Bacteroidetes/Chlorobi group;Bacteroidetes;Sphingobacteriia;Sphingobacteriales;Sphingobacteriaceae;Pedobacter; | 0 | 0 | 0 | 4 |  |
| Bacteria;Firmicutes;Tissierellia;Tissierellales;Peptoniphilaceae;Anaerococcus;Anaerococcus octavius; | 13 | 0 | 0 | 0 |  |
| Bacteria;Proteobacteria; | 210 | 111 | 291 | 273 |  |
| Bacteria;Proteobacteria;Alphaproteobacteria; | 0 | 0 | 0 | 1 |  |
| Bacteria;Proteobacteria;Alphaproteobacteria;Rhizobiales;Methylobacteriaceae;Methylobacterium; | 0 | 4 | 0 | 0 |  |
| Bacteria;Proteobacteria;Alphaproteobacteria;Rhizobiales;Rhizobiaceae;Rhizobium/Agrobacterium group; | 0 | 0 | 0 | 2 |  |
| Bacteria;Proteobacteria;Betaproteobacteria; | 0 | 0 | 0 | 2 |  |
| Bacteria;Proteobacteria;Betaproteobacteria;Burkholderiales; | 13 | 0 | 0 | 2 |  |
| Bacteria;Proteobacteria;Betaproteobacteria;Burkholderiales;Burkholderiaceae; | 7 | 0 | 6 | 0 |  |
| Bacteria;Proteobacteria;Betaproteobacteria;Burkholderiales;Burkholderiaceae;Ralstonia; | 0 | 0 | 6 | 0 |  |
| Bacteria;Proteobacteria;Betaproteobacteria;Nitrosomonadales;Thiobacillaceae;Thiobacillus; | 3 | 0 | 0 | 3 |  |
| Bacteria;Proteobacteria;Betaproteobacteria;Nitrosomonadales;Thiobacillaceae;Thiobacillus;Thiobacillus denitrificans; | 0 | 4 | 0 | 0 |  |
| Bacteria;Proteobacteria;Gammaproteobacteria;Aeromonadales;Aeromonadaceae;Aeromonas; | 0 | 0 | 6 | 0 |  |
| Bacteria;Proteobacteria;Gammaproteobacteria;Pseudomonadales; | 0 | 0 | 3 | 0 |  |
| Bacteria;Proteobacteria;Gammaproteobacteria;Xanthomonadales; | 0 | 0 | 3 | 0 |  |
| Bacteria;Proteobacteria;Gammaproteobacteria;Xanthomonadales;Rhodanobacteraceae; | 0 | 0 | 0 | 2 |  |
| Bacteria;Proteobacteria;Gammaproteobacteria;Xanthomonadales;Xanthomonadaceae;Pseudoxanthomonas; | 0 | 0 | 0 | 1 |  |
| Bacteria;Proteobacteria;Hydrogenophilalia;Hydrogenophilales;unclassified Hydrogenophilales;Hydrogenophilales sp. | 0 | 0 | 0 | 0 |  |

| **Taxa** | **MFC A anode** | **MFC B anode** | **MFC A anolyte** | **MFC B anolyte** | **FDR** |
| --- | --- | --- | --- | --- | --- |
| **Ammonium assimilation** |  |  |  |  |  |
|  |  |  |  |  |  |
| **Glutamine synthetase Type I *glnA*** |  |  |  |  | 1 |
| Bacteria; | 104 | 43 | 41 | 15 |  |
| Bacteria;Actinobacteria;Actinobacteria; | 0 | 72 | 108 | 30 |  |
| Bacteria;Actinobacteria;Actinobacteria;Corynebacteriales; | 20 | 0 | 0 | 0 |  |
| Bacteria;Actinobacteria;Actinobacteria;Corynebacteriales;Corynebacteriaceae;Corynebacterium; | 13 | 0 | 0 | 1 |  |
| Bacteria;Actinobacteria;Actinobacteria;Corynebacteriales;Nocardiaceae;Rhodococcus; | 10 | 0 | 3 | 0 |  |
| Bacteria;Actinobacteria;Actinobacteria;Corynebacteriales;Nocardiaceae;Rhodococcus;Rhodococcus erythropolis | 3 | 0 | 0 | 0 |  |
| Bacteria;Actinobacteria;Actinobacteria;Corynebacteriales;unclassified Corynebacteriales;Lawsonella;Lawsonella clevelandensis | 7 | 0 | 0 | 0 |  |
| Bacteria;Actinobacteria;Actinobacteria;Micrococcales; | 13 | 22 | 12 | 35 |  |
| Bacteria;Actinobacteria;Actinobacteria;Micrococcales;Microbacteriaceae; | 53 | 169 | 119 | 80 |  |
| Bacteria;Actinobacteria;Actinobacteria;Micrococcales;Microbacteriaceae;Clavibacter; | 0 | 7 | 0 | 0 |  |
| Bacteria;Actinobacteria;Actinobacteria;Micrococcales;Microbacteriaceae;Cryobacterium;Cryobacterium mesophilum; | 0 | 0 | 15 | 3 |  |
| Bacteria;Actinobacteria;Actinobacteria;Micrococcales;Microbacteriaceae;Leifsonia; | 0 | 0 | 6 | 0 |  |
| Bacteria;Actinobacteria;Actinobacteria;Micrococcales;Microbacteriaceae;Rathayibacter;Rathayibacter toxicus; | 0 | 0 | 0 | 2 |  |
| Bacteria;Actinobacteria;Actinobacteria;Propionibacteriales;Nocardioidaceae; | 13 | 0 | 0 | 0 |  |
| Bacteria;Actinobacteria;Actinobacteria;Propionibacteriales;Propionibacteriaceae; | 140 | 7 | 0 | 0 |  |
| Bacteria;Actinobacteria;Actinobacteria;Propionibacteriales;Propionibacteriaceae;Cutibacterium; | 20 | 7 | 0 | 0 |  |
| Bacteria;Firmicutes;Bacilli;Bacillales; | 0 | 0 | 6 | 0 |  |
| Bacteria;Proteobacteria; | 539 | 949 | 741 | 985 |  |
| Bacteria;Proteobacteria;Alphaproteobacteria; | 63 | 32 | 23 | 3 |  |
| Bacteria;Proteobacteria;Alphaproteobacteria;Rhizobiales; | 50 | 50 | 23 | 3 |  |
| Bacteria;Proteobacteria;Alphaproteobacteria;Rhizobiales;Methylobacteriaceae;Methylobacterium; | 0 | 7 | 0 | 1 |  |
| Bacteria;Proteobacteria;Alphaproteobacteria;Sphingomonadales; | 20 | 0 | 6 | 0 |  |
| Bacteria;Proteobacteria;Betaproteobacteria; | 37 | 0 | 15 | 5 |  |
| Bacteria;Proteobacteria;Betaproteobacteria;Burkholderiales; | 0 | 0 | 12 | 2 |  |
| Bacteria;Proteobacteria;Betaproteobacteria;Nitrosomonadales; | 0 | 0 | 0 | 2 |  |
| Bacteria;Proteobacteria;Betaproteobacteria;Nitrosomonadales;Gallionellaceae;Sulfuricella;Sulfuricella denitrificans; | 3 | 0 | 0 | 0 |  |
| Bacteria;Proteobacteria;Betaproteobacteria;Nitrosomonadales;Thiobacillaceae;Thiobacillus; | 0 | 0 | 6 | 3 |  |
| Bacteria;Proteobacteria;Gammaproteobacteria; | 0 | 0 | 0 | 1 |  |
| Bacteria;Proteobacteria;Gammaproteobacteria;Enterobacterales; | 0 | 7 | 0 | 0 |  |
| Bacteria;Proteobacteria;Gammaproteobacteria;Pseudomonadales;Moraxellaceae; | 13 | 0 | 0 | 0 |  |
| Bacteria;Proteobacteria;Gammaproteobacteria;Pseudomonadales;Pseudomonadaceae;Pseudomonas; | 13 | 0 | 12 | 0 |  |
| Bacteria;Proteobacteria;Gammaproteobacteria;Xanthomonadales; | 0 | 0 | 6 | 0 |  |
| Bacteria;Proteobacteria;Gammaproteobacteria;Xanthomonadales;Rhodanobacteraceae; | 0 | 0 | 0 | 1 |  |
| Bacteria;Proteobacteria;Gammaproteobacteria;Xanthomonadales;Xanthomonadaceae; | 0 | 0 | 6 | 3 |  |
|  |  |  |  |  |  |
| **Glutamine synthetase Type III *glnN*** |  |  |  |  | 0.97 |
| Bacteria; | 7 | 0 | 6 | 9 |  |
| Bacteria;Proteobacteria; | 0 | 4 | 9 | 276 |  |
| Bacteria;Proteobacteria;Alphaproteobacteria; | 17 | 0 | 0 | 0 |  |
| Bacteria;Proteobacteria;Alphaproteobacteria;Rhizobiales; | 20 | 0 | 0 | 0 |  |
| Bacteria;Proteobacteria;Alphaproteobacteria;Rhizobiales;Methylobacteriaceae;Methylobacterium; | 0 | 14 | 6 | 2 |  |
| Bacteria;Proteobacteria;Alphaproteobacteria;Rhizobiales;Phyllobacteriaceae;Pseudaminobacter; | 3 | 0 | 0 | 0 |  |
| Bacteria;Proteobacteria;Betaproteobacteria;Nitrosomonadales;Thiobacillaceae;Thiobacillus; | 0 | 0 | 0 | 6 |  |
| Bacteria;Proteobacteria;Gammaproteobacteria;Pseudomonadales;Pseudomonadaceae;Pseudomonas; | 13 | 0 | 0 | 0 |  |

| **Taxa** | **MFC A anode** | **MFC B anode** | **MFC A anolyte** | **MFC B anolyte** | **FDR** |
| --- | --- | --- | --- | --- | --- |
| **Carbon dioxide fixation** |  |  |  |  |  |
|  |  |  |  |  |  |
| **CBB cycle - Phosphoglycerate kinase *pgk*** |  |  |  |  | 1 |
| Bacteria; | 13 | 11 | 29 | 3 |  |
| Bacteria;Actinobacteria;Actinobacteria; | 3 | 18 | 6 | 16 |  |
| Bacteria;Actinobacteria;Actinobacteria;Micrococcales; | 0 | 0 | 6 | 5 |  |
| Bacteria;Actinobacteria;Actinobacteria;Micrococcales;Microbacteriaceae; | 0 | 0 | 9 | 8 |  |
| Bacteria;Actinobacteria;Actinobacteria;Propionibacteriales;Propionibacteriaceae; | 53 | 0 | 0 | 0 |  |
| Bacteria;Firmicutes;Bacilli;Bacillales;Bacillales incertae sedis;Bacillales Family XI. Incertae Sedis;Gemella; | 0 | 7 | 0 | 0 |  |
| Bacteria;Firmicutes;Negativicutes;Veillonellales;Veillonellaceae;Veillonella; | 0 | 4 | 0 | 0 |  |
| Bacteria;Firmicutes;Tissierellia;Tissierellales;Peptoniphilaceae; | 27 | 0 | 0 | 0 |  |
| Bacteria;Proteobacteria; | 127 | 190 | 323 | 410 |  |
| Bacteria;Proteobacteria;Alphaproteobacteria;Caulobacterales; | 0 | 0 | 15 | 0 |  |
| Bacteria;Proteobacteria;Alphaproteobacteria;Sphingomonadales;Sphingomonadaceae;Sphingomonas; | 13 | 0 | 0 | 0 |  |
| Bacteria;Proteobacteria;Betaproteobacteria; | 0 | 0 | 6 | 5 |  |
| Bacteria;Proteobacteria;Betaproteobacteria;Burkholderiales; | 0 | 14 | 6 | 3 |  |
| Bacteria;Proteobacteria;Betaproteobacteria;Burkholderiales;Burkholderiaceae; | 0 | 14 | 0 | 0 |  |
| Bacteria;Proteobacteria;Betaproteobacteria;Burkholderiales;Comamonadaceae; | 0 | 0 | 0 | 2 |  |
| Bacteria;Proteobacteria;Betaproteobacteria;Nitrosomonadales;Thiobacillaceae;Thiobacillus; | 0 | 0 | 0 | 4 |  |
| Bacteria;Proteobacteria;Gammaproteobacteria;Xanthomonadales; | 0 | 0 | 0 | 1 |  |
| Bacteria;Proteobacteria;Gammaproteobacteria;Xanthomonadales;Xanthomonadaceae; | 0 | 0 | 0 | 1 |  |
|  |  |  |  |  |  |
| **CBB cycle - Glyceraldehyde-3-phosphate dehydrogenase *gap2*** |  |  |  |  | 1 |
| Bacteria; | 317 | 241 | 533 | 117 |  |
| Bacteria;Actinobacteria; | 0 | 7 | 3 | 1 |  |
| Bacteria;Actinobacteria;Actinobacteria; | 170 | 97 | 433 | 106 |  |
| Bacteria;Actinobacteria;Actinobacteria;Corynebacteriales;Corynebacteriaceae;Corynebacterium; | 7 | 0 | 0 | 0 |  |
| Bacteria;Actinobacteria;Actinobacteria;Corynebacteriales;Nocardiaceae;Rhodococcus; | 0 | 0 | 0 | 1 |  |
| Bacteria;Actinobacteria;Actinobacteria;Micrococcales; | 90 | 57 | 90 | 13 |  |
| Bacteria;Actinobacteria;Actinobacteria;Micrococcales;Microbacteriaceae; | 300 | 147 | 352 | 36 |  |
| Bacteria;Actinobacteria;Actinobacteria;Micrococcales;Microbacteriaceae;Cryobacterium;Cryobacterium mesophilum; | 30 | 7 | 49 | 12 |  |
| Bacteria;Actinobacteria;Actinobacteria;Micrococcales;Microbacteriaceae;Microbacterium; | 40 | 0 | 0 | 0 |  |
| Bacteria;Actinobacteria;Actinobacteria;Micrococcales;Micrococcaceae;Arthrobacter;Arthrobacter sp. | 0 | 0 | 0 | 1 |  |
| Bacteria;Actinobacteria;Actinobacteria;Micrococcales;Micrococcaceae;Glutamicibacter;Glutamicibacter sp. | 3 | 4 | 12 | 1 |  |
| Bacteria;Actinobacteria;Actinobacteria;Micrococcales;unclassified Micrococcales;unclassified Micrococcales | 0 | 4 | 0 | 1 |  |
| Bacteria;Actinobacteria;Actinobacteria;Propionibacteriales;Nocardioidaceae; | 13 | 0 | 0 | 0 |  |
| Bacteria;Actinobacteria;Actinobacteria;Propionibacteriales;Propionibacteriaceae; | 53 | 0 | 0 | 0 |  |
| Bacteria;Actinobacteria;unclassified Actinobacteria;Actinobacteria bacterium | 7 | 0 | 0 | 0 |  |
| Bacteria;Firmicutes;Bacilli;Bacillales; | 7 | 0 | 0 | 0 |  |
| Bacteria;Firmicutes;Bacilli;Lactobacillales;Streptococcaceae;Streptococcus; | 0 | 7 | 0 | 0 |  |
| Bacteria;Firmicutes;Clostridia;Clostridiales;Clostridiales incertae sedis;Clostridiales Family XIII. Incertae Sedis | 0 | 4 | 0 | 0 |  |
| Bacteria;Firmicutes;Negativicutes;Veillonellales;Veillonellaceae;Veillonella; | 0 | 4 | 0 | 0 |  |
| Bacteria;FCB group;Bacteroidetes/Chlorobi group;Bacteroidetes;Sphingobacteriia;Sphingobacteriales;Sphingobacteriaceae; | 0 | 14 | 0 | 3 |  |
| Bacteria;Proteobacteria; | 230 | 320 | 334 | 572 |  |
| Bacteria;Proteobacteria;Alphaproteobacteria; | 77 | 0 | 0 | 2 |  |
| Bacteria;Proteobacteria;Alphaproteobacteria;Caulobacterales; | 0 | 0 | 3 | 0 |  |
| Bacteria;Proteobacteria;Alphaproteobacteria;Rhizobiales; | 7 | 0 | 0 | 2 |  |
| Bacteria;Proteobacteria;Alphaproteobacteria;Rhizobiales;Bradyrhizobiaceae;Tardiphaga;Tardiphaga robiniae; | 0 | 0 | 0 | 1 |  |
| Bacteria;Proteobacteria;Alphaproteobacteria;Rhizobiales;Phyllobacteriaceae; | 0 | 4 | 0 | 0 |  |
| Bacteria;Proteobacteria;Alphaproteobacteria;Rhizobiales;Phyllobacteriaceae;Mesorhizobium; | 0 | 4 | 0 | 0 |  |
| Bacteria;Proteobacteria;Alphaproteobacteria;Rhizobiales;unclassified Rhizobiales;unclassified Rhizobiales; | 30 | 0 | 0 | 0 |  |
| Bacteria;Proteobacteria;Alphaproteobacteria;Sphingomonadales;Sphingomonadaceae; | 7 | 4 | 0 | 0 |  |
| Bacteria;Proteobacteria;Alphaproteobacteria;Sphingomonadales;Sphingomonadaceae;Sphingomonas; | 7 | 0 | 0 | 0 |  |
| Bacteria;Proteobacteria;Betaproteobacteria; | 7 | 0 | 73 | 6 |  |
| Bacteria;Proteobacteria;Betaproteobacteria;Burkholderiales; | 0 | 0 | 15 | 0 |  |
| Bacteria;Proteobacteria;Betaproteobacteria;Burkholderiales;Comamonadaceae; | 0 | 0 | 6 | 0 |  |
| Bacteria;Proteobacteria;Betaproteobacteria;Nitrosomonadales;Thiobacillaceae;Thiobacillus; | 3 | 0 | 3 | 1 |  |
| Bacteria;Proteobacteria;Gammaproteobacteria; | 3 | 0 | 15 | 0 |  |
| Bacteria;Proteobacteria;Gammaproteobacteria;Pseudomonadales;Pseudomonadaceae;Pseudomonas; | 70 | 0 | 0 | 0 |  |
| Bacteria;Proteobacteria;Gammaproteobacteria;unclassified Gammaproteobacteria;Candidatus Thioglobus;;uncultured | 0 | 7 | 0 | 0 |  |
| Bacteria;Proteobacteria;Gammaproteobacteria;Xanthomonadales; | 0 | 0 | 6 | 0 |  |
|  |  |  |  |  |  |
| **CBB cycle - Triosephosphate isomerase *tpiA*** |  |  |  |  | 1 |
| Bacteria; | 10 | 7 | 0 | 14 |  |
| Bacteria;Actinobacteria;Actinobacteria; | 33 | 0 | 35 | 31 |  |
| Bacteria;Actinobacteria;Actinobacteria;Micrococcales; | 0 | 0 | 12 | 5 |  |
| Bacteria;Actinobacteria;Actinobacteria;Micrococcales;Microbacteriaceae; | 0 | 0 | 67 | 11 |  |
| Bacteria;Actinobacteria;Actinobacteria;Micrococcales;Microbacteriaceae;Cryobacterium;Cryobacterium mesophilum; | 3 | 0 | 6 | 3 |  |
| Bacteria;Actinobacteria;Actinobacteria;Micrococcales;Microbacteriaceae;Microbacterium; | 0 | 0 | 6 | 0 |  |
| Bacteria;Actinobacteria;Actinobacteria;Propionibacteriales;Propionibacteriaceae; | 27 | 0 | 0 | 0 |  |
| Bacteria;Actinobacteria;Actinobacteria;Propionibacteriales;Propionibacteriaceae;Cutibacterium; | 27 | 0 | 0 | 0 |  |
| Bacteria;FCB group;Bacteroidetes/Chlorobi group;Bacteroidetes;Sphingobacteriia;Sphingobacteriales;Sphingobacteriaceae; | 0 | 14 | 0 | 0 |  |
| Bacteria;Proteobacteria; | 80 | 190 | 52 | 319 |  |
| Bacteria;Proteobacteria;Alphaproteobacteria;Caulobacterales;Caulobacteraceae;Caulobacter; | 0 | 14 | 6 | 1 |  |
| Bacteria;Proteobacteria;Alphaproteobacteria;Rhizobiales;Phyllobacteriaceae; | 10 | 0 | 0 | 0 |  |
| Bacteria;Proteobacteria;Alphaproteobacteria;Rhizobiales;Phyllobacteriaceae;Mesorhizobium; | 7 | 0 | 0 | 0 |  |
| Bacteria;Proteobacteria;Betaproteobacteria;Burkholderiales; | 7 | 0 | 3 | 5 |  |
| Bacteria;Proteobacteria;Betaproteobacteria;Burkholderiales;Comamonadaceae; | 0 | 0 | 3 | 0 |  |
| Bacteria;Proteobacteria;Betaproteobacteria;Burkholderiales;unclassified Burkholderiales;unclassified Burkholderiales | 7 | 0 | 0 | 0 |  |
| Bacteria;Proteobacteria;Betaproteobacteria;Nitrosomonadales;Thiobacillaceae;Thiobacillus; | 0 | 0 | 3 | 4 |  |
| Bacteria;Proteobacteria;Gammaproteobacteria; | 3 | 0 | 0 | 0 |  |
| Bacteria;Proteobacteria;Gammaproteobacteria;Pseudomonadales;Pseudomonadaceae;Pseudomonas; | 3 | 4 | 0 | 0 |  |
| Bacteria;Proteobacteria;Gammaproteobacteria;Xanthomonadales;Rhodanobacteraceae;Rhodanobacter; | 0 | 4 | 0 | 0 |  |
| Bacteria;Proteobacteria;Gammaproteobacteria;Xanthomonadales;Xanthomonadaceae; | 0 | 0 | 6 | 0 |  |
| Bacteria;Proteobacteria;Gammaproteobacteria;Xanthomonadales;Xanthomonadaceae;Pseudoxanthomonas; | 0 | 0 | 6 | 2 |  |
| Bacteria;Proteobacteria;Gammaproteobacteria;Xanthomonadales;Xanthomonadaceae;Pseudoxanthomonas sp. | 0 | 0 | 0 | 1 |  |
|  |  |  |  |  |  |
| **CBB cycle - Fructose-bisphosphate aldolase *fbaAB*** |  |  |  |  | 1 |
| Bacteria; | 266 | 474 | 285 | 313 |  |
| Bacteria;Actinobacteria;Actinobacteria; | 87 | 0 | 113 | 40 |  |
| Bacteria;Actinobacteria;Actinobacteria;Corynebacteriales; | 7 | 0 | 0 | 0 |  |
| Bacteria;Actinobacteria;Actinobacteria;Corynebacteriales;Nocardiaceae;Rhodococcus; | 3 | 0 | 0 | 0 |  |
| Bacteria;Actinobacteria;Actinobacteria;Micrococcales; | 40 | 0 | 70 | 14 |  |
| Bacteria;Actinobacteria;Actinobacteria;Micrococcales;Microbacteriaceae; | 127 | 29 | 148 | 34 |  |
| Bacteria;Actinobacteria;Actinobacteria;Micrococcales;Microbacteriaceae;Cryobacterium;Cryobacterium mesophilum; | 0 | 0 | 0 | 1 |  |
| Bacteria;Actinobacteria;Actinobacteria;Micrococcales;Microbacteriaceae;Microbacterium; | 3 | 0 | 0 | 0 |  |
| Bacteria;Actinobacteria;Actinobacteria;Micrococcales;Microbacteriaceae;Salinibacterium; | 0 | 0 | 6 | 3 |  |
| Bacteria;Actinobacteria;Actinobacteria;Propionibacteriales;Propionibacteriaceae; | 67 | 0 | 0 | 0 |  |
| Bacteria;Actinobacteria;Actinobacteria;Propionibacteriales;Propionibacteriaceae;Cutibacterium;Cutibacterium acnes; | 7 | 0 | 0 | 0 |  |
| Bacteria;Actinobacteria;Actinobacteria;Propionibacteriales;Propionibacteriaceae;Cutibacterium;Cutibacterium acnes;Propionibacterium acnes | 20 | 0 | 0 | 0 |  |
| Bacteria;FCB group;Bacteroidetes/Chlorobi group;Bacteroidetes; | 0 | 0 | 0 | 3 |  |
| Bacteria;FCB group;Bacteroidetes/Chlorobi group;Bacteroidetes;Chitinophagia;Chitinophagales;Chitinophagaceae;unclassified Chitinophagaceae;Chitinophagaceae sp. | 0 | 0 | 0 | 1 |  |
| Bacteria;FCB group;Bacteroidetes/Chlorobi group;Bacteroidetes;Flavobacteriia;Flavobacteriales;Flavobacteriaceae;Chryseobacterium;Chryseobacterium jeonii; | 0 | 14 | 0 | 5 |  |
| Bacteria;Proteobacteria; | 1095 | 650 | 489 | 572 |  |
| Bacteria;Proteobacteria;Alphaproteobacteria; | 7 | 0 | 3 | 1 |  |
| Bacteria;Proteobacteria;Alphaproteobacteria;Caulobacterales; | 0 | 0 | 6 | 0 |  |
| Bacteria;Proteobacteria;Alphaproteobacteria;Caulobacterales;Caulobacteraceae;Phenylobacterium; | 0 | 0 | 17 | 0 |  |
| Bacteria;Proteobacteria;Alphaproteobacteria;Rhizobiales;Bradyrhizobiaceae; | 0 | 4 | 0 | 0 |  |
| Bacteria;Proteobacteria;Alphaproteobacteria;Rhizobiales;Phyllobacteriaceae;Mesorhizobium; | 0 | 0 | 0 | 2 |  |
| Bacteria;Proteobacteria;Betaproteobacteria; | 0 | 0 | 3 | 3 |  |
| Bacteria;Proteobacteria;Betaproteobacteria;Burkholderiales; | 27 | 0 | 0 | 3 |  |
| Bacteria;Proteobacteria;Betaproteobacteria;Burkholderiales;Alcaligenaceae; | 0 | 0 | 0 | 1 |  |
| Bacteria;Proteobacteria;Betaproteobacteria;Nitrosomonadales; | 0 | 0 | 0 | 9 |  |
| Bacteria;Proteobacteria;Betaproteobacteria;Nitrosomonadales;Thiobacillaceae;Thiobacillus; | 0 | 32 | 0 | 18 |  |
| Bacteria;Proteobacteria;Gammaproteobacteria; | 7 | 0 | 0 | 0 |  |
| Bacteria;Proteobacteria;Gammaproteobacteria;Enterobacterales; | 7 | 0 | 0 | 0 |  |
| Bacteria;Proteobacteria;Gammaproteobacteria;Xanthomonadales; | 0 | 0 | 0 | 2 |  |
| Bacteria;Proteobacteria;Gammaproteobacteria;Xanthomonadales;Xanthomonadaceae; | 0 | 0 | 0 | 2 |  |
| Bacteria;Proteobacteria;Hydrogenophilalia;Hydrogenophilales; | 23 | 29 | 6 | 7 |  |
| Bacteria;Proteobacteria;Hydrogenophilalia;Hydrogenophilales;unclassified Hydrogenophilales; | 0 | 4 | 0 | 4 |  |
|  |  |  |  |  |  |
| **CBB cycle - fructose 1,6-bisphosphatase *fpb*** |  |  |  |  | 1 |
| Bacteria; | 20 | 0 | 15 | 6 |  |
| Bacteria;Actinobacteria;Actinobacteria; | 17 | 7 | 9 | 3 |  |
| Bacteria;Actinobacteria;Actinobacteria;Corynebacteriales; | 17 | 0 | 0 | 0 |  |
| Bacteria;Actinobacteria;Actinobacteria;Corynebacteriales;Nocardiaceae; | 13 | 0 | 0 | 0 |  |
| Bacteria;Actinobacteria;Actinobacteria;Corynebacteriales;Nocardiaceae;Rhodococcus; | 27 | 0 | 0 | 0 |  |
| Bacteria;Actinobacteria;Actinobacteria;Micrococcales; | 0 | 14 | 0 | 1 |  |
| Bacteria;Actinobacteria;Actinobacteria;Micrococcales;Microbacteriaceae; | 7 | 75 | 323 | 26 |  |
| Bacteria;Actinobacteria;Actinobacteria;Micrococcales;Microbacteriaceae;Cryobacterium; | 0 | 0 | 3 | 0 |  |
| Bacteria;Actinobacteria;Actinobacteria;Micrococcales;Microbacteriaceae;Cryobacterium;Cryobacterium mesophilum; | 7 | 22 | 134 | 16 |  |
| Bacteria;Firmicutes;Bacilli;Bacillales;Bacillales incertae sedis;Bacillales Family XI. Incertae Sedis;Gemella; | 0 | 7 | 0 | 0 |  |
| Bacteria;Firmicutes;Bacilli;Bacillales;Bacillales incertae sedis;Bacillales Family XI. Incertae Sedis;Gemella;Gemella haemolysans | 0 | 7 | 0 | 0 |  |
| Bacteria;Proteobacteria; | 496 | 913 | 352 | 480 |  |
| Bacteria;Proteobacteria;Alphaproteobacteria;Sphingomonadales; | 7 | 0 | 0 | 0 |  |
| Bacteria;Proteobacteria;Alphaproteobacteria;Sphingomonadales;Sphingomonadaceae;Sphingomonas;Sphingomonas sanguinis | 7 | 0 | 0 | 0 |  |
| Bacteria;Proteobacteria;Alphaproteobacteria;Sphingomonadales;Sphingomonadaceae;Sphingopyxis; | 0 | 0 | 6 | 0 |  |
| Bacteria;Proteobacteria;Betaproteobacteria; | 0 | 0 | 3 | 1 |  |
| Bacteria;Proteobacteria;Betaproteobacteria;Burkholderiales; | 0 | 0 | 12 | 0 |  |
| Bacteria;Proteobacteria;Betaproteobacteria;Burkholderiales;Comamonadaceae;Hydrogenophaga; | 3 | 0 | 15 | 0 |  |
| Bacteria;Proteobacteria;Gammaproteobacteria; | 3 | 0 | 0 | 0 |  |
| Bacteria;Proteobacteria;Gammaproteobacteria;Pseudomonadales;Pseudomonadaceae;Pseudomonas; | 13 | 0 | 0 | 0 |  |
| Bacteria;Proteobacteria;Gammaproteobacteria;Xanthomonadales; | 0 | 0 | 0 | 2 |  |
| Bacteria;Proteobacteria;Gammaproteobacteria;Xanthomonadales;Xanthomonadaceae; | 3 | 0 | 0 | 1 |  |
|  |  |  |  |  |  |
| **CBB cycle - phosphoribulokinase *prk*** |  |  |  |  | 1 |
| Bacteria; | 70 | 54 | 79 | 97 |  |
| Bacteria;Proteobacteria; | 683 | 1053 | 425 | 1325 |  |
| Bacteria;Proteobacteria;Betaproteobacteria; | 7 | 0 | 55 | 3 |  |
| Bacteria;Proteobacteria;Betaproteobacteria;Burkholderiales; | 7 | 0 | 35 | 0 |  |
| Bacteria;Proteobacteria;Betaproteobacteria;Burkholderiales;Comamonadaceae;Hydrogenophaga;Hydrogenophaga palleronii | 0 | 0 | 26 | 2 |  |
| Bacteria;Proteobacteria;Betaproteobacteria;Nitrosomonadales;Thiobacillaceae;Thiobacillus; | 0 | 0 | 0 | 2 |  |
| Bacteria;Proteobacteria;Gammaproteobacteria;Chromatiales;Chromatiaceae;Allochromatium;Allochromatium warmingii; | 0 | 0 | 0 | 0 |  |
| Bacteria;Proteobacteria;Gammaproteobacteria;Chromatiales;Ectothiorhodospiraceae;Thioalkalivibrio; | 0 | 0 | 0 | 0 |  |
| Bacteria;Proteobacteria;Hydrogenophilalia;Hydrogenophilales;unclassified Hydrogenophilales;Hydrogenophilales sp. | 0 | 4 | 0 | 2 |  |
|  |  |  |  |  |  |
| **CBB cycle - ribose 5-phosphate isomerase *rpiA*** |  |  |  |  | 1 |
| Bacteria; | 6 | 0 | 4 | 1 |  |
| Bacteria;Actinobacteria;Actinobacteria; | 0 | 7 | 15 | 0 |  |
| Bacteria;Actinobacteria;Actinobacteria;Micrococcales; | 0 | 0 | 3 | 0 |  |
| Bacteria;Actinobacteria;Actinobacteria;Micrococcales;Intrasporangiaceae; | 0 | 0 | 3 | 0 |  |
| Bacteria;Actinobacteria;Actinobacteria; | 13 | 11 | 0 | 5 |  |
| Bacteria;Actinobacteria;Actinobacteria;Micrococcales; | 0 | 36 | 0 | 8 |  |
| Bacteria;Actinobacteria;Actinobacteria;Micrococcales;Microbacteriaceae; | 0 | 0 | 3 | 4 |  |
| Bacteria;Actinobacteria;Actinobacteria;Micrococcales;Microbacteriaceae;Cryobacterium;Cryobacterium mesophilum; | 0 | 0 | 0 | 1 |  |
| Bacteria;Actinobacteria;Actinobacteria;Micrococcales;Micrococcaceae; | 13 | 0 | 0 | 0 |  |
| Bacteria;FCB group;Bacteroidetes/Chlorobi group;Bacteroidetes;Sphingobacteriia;Sphingobacteriales; | 0 | 0 | 0 | 1 |  |
| Bacteria;FCB group;Bacteroidetes/Chlorobi group;Bacteroidetes;Sphingobacteriia;Sphingobacteriales;Sphingobacteriaceae; | 3 | 0 | 0 | 1 |  |
| Bacteria;Proteobacteria; | 107 | 93 | 81 | 269 |  |
| Bacteria;Proteobacteria;Alphaproteobacteria;Rhodobacterales; | 13 | 0 | 0 | 0 |  |
| Bacteria;Proteobacteria;Betaproteobacteria;Burkholderiales; | 0 | 0 | 3 | 3 |  |
| Bacteria;Proteobacteria;Betaproteobacteria;Nitrosomonadales;Thiobacillaceae;Thiobacillus;Thiobacillus sp. | 0 | 0 | 0 | 1 |  |
| Bacteria;Proteobacteria;Gammaproteobacteria;Xanthomonadales;Rhodanobacteraceae;Rhodanobacter; | 3 | 0 | 0 | 0 |  |
| Bacteria;Proteobacteria;Gammaproteobacteria;Pseudomonadales;Pseudomonadaceae; | 3 | 0 | 0 | 0 |  |
| Bacteria;Proteobacteria;Gammaproteobacteria;Pseudomonadales;Pseudomonadaceae;Pseudomonas; | 3 | 0 | 0 | 0 |  |
|  |  |  |  |  |  |
| **Reductive TCA I - ATP-citrate lyase *AclAB*** |  |  |  |  | 1 |
| Bacteria; | 0 | 0 | 0 | 3 |  |
| Bacteria;Actinobacteria;Actinobacteria; | 13 | 0 | 0 | 4 |  |
| Bacteria;Actinobacteria;Actinobacteria;Micrococcales; | 0 | 0 | 0 | 13 |  |
| Bacteria;Actinobacteria;Actinobacteria;Micrococcales;Microbacteriaceae; | 0 | 0 | 0 | 2 |  |
| Bacteria;Actinobacteria;Actinobacteria;Micrococcales;Microbacteriaceae;Cryobacterium;Cryobacterium mesophilum | 0 | 0 | 0 | 6 |  |
| Bacteria;Actinobacteria;Actinobacteria;Propionibacteriales;Propionibacteriaceae; | 37 | 0 | 0 | 0 |  |
| Bacteria;Actinobacteria;Actinobacteria;Propionibacteriales;Propionibacteriaceae;Cutibacterium; | 43 | 0 | 0 | 0 |  |
| Bacteria;Proteobacteria; | 40 | 4 | 20 | 3 |  |
| Bacteria;Proteobacteria;Alphaproteobacteria; | 3 | 0 | 0 | 0 |  |
| Bacteria;Proteobacteria;Alphaproteobacteria;Caulobacterales;Caulobacteraceae;Phenylobacterium sp. | 0 | 0 | 6 | 0 |  |
| Bacteria;Proteobacteria;Alphaproteobacteria;Rhodobacterales; | 7 | 0 | 0 | 0 |  |
| Bacteria;Proteobacteria;Alphaproteobacteria;Rhodobacterales;Rhodobacteraceae; | 7 | 0 | 0 | 0 |  |
| Bacteria;Proteobacteria;Alphaproteobacteria;Sphingomonadales;Sphingomonadaceae;Sphingomonas; | 0 | 0 | 6 | 2 |  |
| Bacteria;Proteobacteria;Alphaproteobacteria;Sphingomonadales;Sphingomonadaceae;Sphingomonas paucimobilis | 13 | 0 | 0 | 0 |  |
| Bacteria;Proteobacteria;Betaproteobacteria; | 0 | 0 | 6 | 0 |  |
| Bacteria;Proteobacteria;Betaproteobacteria;Burkholderiales; | 13 | 0 | 64 | 12 |  |
| Bacteria;Proteobacteria;Betaproteobacteria;Burkholderiales;Burkholderiaceae; | 13 | 0 | 0 | 0 |  |
| Bacteria;Proteobacteria;Betaproteobacteria;Burkholderiales;Burkholderiaceae;Cupriavidus; | 0 | 0 | 6 | 2 |  |
| Bacteria;Proteobacteria;Gammaproteobacteria;Xanthomonadales;Rhodanobacteraceae; | 0 | 0 | 0 | 1 |  |
| Bacteria;Proteobacteria;Gammaproteobacteria;Xanthomonadales;Xanthomonadaceae;Pseudoxanthomonas; | 0 | 0 | 0 | 6 |  |
|  |  |  |  |  |  |
| **Wood Ljungdahl - carbon monoxide dehydrogenase/acetyl-CoA synthase *coxLMS*** |  |  |  |  | 0.28 |
| Bacteria; | 7 | 248 | 0 | 0 |  |
| Bacteria;Proteobacteria; | 0 | 4 | 0 | 0 |  |
| Bacteria;Proteobacteria;Alphaproteobacteria; | 0 | 18 | 0 | 0 |  |
| Bacteria;Proteobacteria;Alphaproteobacteria;Rhizobiales; | 7 | 32 | 0 | 0 |  |
| Bacteria;Proteobacteria;Alphaproteobacteria;Rhizobiales;unclassified Rhizobiales;unclassified Rhizobiales; | 0 | 18 | 0 | 0 |  |
| Bacteria;Proteobacteria; | 0 | 0 | 0 | 0 |  |
| Bacteria;Proteobacteria;Betaproteobacteria;Nitrosomonadales;Thiobacillaceae;Thiobacillus;Thiobacillus sp. SCN 62-729; | 0 | 0 | 3 | 2 |  |

| **Taxa** | **MFC A anode** | **MFC B anode** | **MFC A anolyte** | **MFC B anolyte** | **FDR** |
| --- | --- | --- | --- | --- | --- |
| **Cold adaption** |  |  |  |  |  |
|  |  |  |  |  |  |
| **Cryoprotectants - betaine aldehyde dehydrogenase *betB*** |  |  |  |  | 0.96 |
| Bacteria; | 7 | 0 | 3 | 0 |  |
| Bacteria;Actinobacteria;Actinobacteria;Micrococcales;Microbacteriaceae;Cryobacterium;Cryobacterium mesophilum; | 0 | 0 | 6 | 1 |  |
| Bacteria;Proteobacteria; | 153 | 988 | 142 | 236 |  |
| Bacteria;Proteobacteria;Alphaproteobacteria;Rhizobiales;Phyllobacteriaceae;Mesorhizobium; | 20 | 0 | 0 | 0 |  |
| Bacteria;Proteobacteria;Gammaproteobacteria;Pseudomonadales;Pseudomonadaceae;Pseudomonas; | 0 | 29 | 0 | 0 |  |
|  |  |  |  |  |  |
| **Cryoprotectants - betaine-choline-carnitine family transporter** |  |  |  |  | 1 |
| Bacteria; | 7 | 0 | 0 | 4 |  |
| Bacteria;Actinobacteria;Actinobacteria;Corynebacteriales; | 7 | 0 | 0 | 0 |  |
| Bacteria;Actinobacteria;Actinobacteria;Corynebacteriales;Corynebacteriaceae;Corynebacterium;Corynebacterium matruchotii | 7 | 0 | 0 | 0 |  |
| Bacteria;Firmicutes;Bacilli;Bacillales; | 13 | 0 | 0 | 0 |  |
| Bacteria;Firmicutes;Bacilli;Bacillales;Staphylococcaceae;Staphylococcus; | 13 | 0 | 0 | 0 |  |
| Bacteria;Proteobacteria; | 13 | 14 | 17 | 9 |  |
| Bacteria;Proteobacteria;Alphaproteobacteria;Sphingomonadales; | 3 | 0 | 0 | 0 |  |
| Bacteria;Proteobacteria;Alphaproteobacteria;Sphingomonadales;Sphingomonadaceae; | 13 | 0 | 6 | 1 |  |
| Bacteria;Proteobacteria;Alphaproteobacteria;Sphingomonadales;Sphingomonadaceae;Sphingomonas; | 0 | 14 | 21 | 1 |  |
| Bacteria;Proteobacteria;Alphaproteobacteria;Sphingomonadales;Sphingomonadaceae;Sphingopyxis; | 0 | 0 | 17 | 0 |  |
| Bacteria;Proteobacteria;Betaproteobacteria; | 0 | 0 | 0 | 1 |  |
| Bacteria;Proteobacteria;Betaproteobacteria;Burkholderiales; | 0 | 0 | 0 | 3 |  |
| Bacteria;Proteobacteria;Betaproteobacteria;Burkholderiales;Alcaligenaceae; | 0 | 0 | 0 | 1 |  |
| Bacteria;Proteobacteria;Betaproteobacteria;Burkholderiales;Alcaligenaceae;Bordetella; | 0 | 0 | 0 | 1 |  |
| Bacteria;Proteobacteria;Betaproteobacteria;Nitrosomonadales;Thiobacillaceae;Thiobacillus | 0 | 0 | 0 | 1 |  |
| Bacteria;Proteobacteria;Gammaproteobacteria; | 7 | 0 | 0 | 0 |  |
| Bacteria;Proteobacteria;Gammaproteobacteria;Pseudomonadales;Pseudomonadaceae;Pseudomonas; | 7 | 7 | 17 | 2 |  |
|  |  |  |  |  |  |
| **Cryoprotectants - glycine betaine ABC transporter *proVWX*** |  |  |  |  | 1 |
| Bacteria; | 0 | 0 | 0 | 4 |  |
| Bacteria;Actinobacteria; | 0 | 0 | 0 | 2 |  |
| Bacteria;Actinobacteria;Actinobacteria; | 53 | 0 | 3 | 11 |  |
| Bacteria;Actinobacteria;Actinobacteria;Micrococcales; | 0 | 0 | 3 | 9 |  |
| Bacteria;Actinobacteria;Actinobacteria;Micrococcales;Microbacteriaceae; | 13 | 0 | 3 | 14 |  |
| Bacteria;Actinobacteria;Actinobacteria;Micrococcales;Microbacteriaceae;Leifsonia;Leifsonia sp. | 0 | 0 | 0 | 1 |  |
| Bacteria;Actinobacteria;Actinobacteria;Propionibacteriales;Propionibacteriaceae; | 113 | 0 | 0 | 0 |  |
| Bacteria;Firmicutes; | 7 | 0 | 0 | 0 |  |
| Bacteria;Firmicutes;Bacilli;Bacillales;Bacillales incertae sedis;Bacillales Family XII. Incertae Sedis;Exiguobacterium; | 27 | 0 | 0 | 0 |  |
| Bacteria;Firmicutes;Tissierellia;Tissierellales;Peptoniphilaceae;Peptoniphilus; | 7 | 0 | 0 | 0 |  |
| Bacteria;Proteobacteria; | 0 | 0 | 0 | 12 |  |
| Bacteria;Proteobacteria;Betaproteobacteria;Burkholderiales;Comamonadaceae; | 0 | 0 | 0 | 0 |  |
| Bacteria;Proteobacteria;Betaproteobacteria;Burkholderiales;Comamonadaceae;Acidovorax; | 0 | 0 | 0 | 1 |  |
| Bacteria;Proteobacteria;Betaproteobacteria;Burkholderiales;Comamonadaceae;Variovorax; | 0 | 0 | 0 | 2 |  |
| Bacteria;Proteobacteria;Gammaproteobacteria;Enterobacterales;Enterobacteriaceae; | 13 | 0 | 0 | 0 |  |
| Bacteria;Proteobacteria;Gammaproteobacteria;Pseudomonadales;Pseudomonadaceae;Pseudomonas; | 0 | 0 | 0 | 2 |  |
|  |  |  |  |  |  |
| **Cold shock proteins *cspA*** |  |  |  |  | 1 |
| Bacteria; | 206 | 176 | 189 | 40 |  |
| Bacteria;Proteobacteria; | 479 | 798 | 1079 | 1113 |  |
| Bacteria;Proteobacteria;Alphaproteobacteria; | 100 | 18 | 47 | 0 |  |
| Bacteria;Proteobacteria;Alphaproteobacteria;Caulobacterales;Caulobacteraceae; | 0 | 0 | 6 | 0 |  |
| Bacteria;Proteobacteria;Alphaproteobacteria;Caulobacterales;Caulobacteraceae;Brevundimonas sp. | 7 | 0 | 0 | 0 |  |
| Bacteria;Proteobacteria;Alphaproteobacteria;Caulobacterales;Caulobacteraceae;Phenylobacterium; | 0 | 0 | 3 | 0 |  |
| Bacteria;Proteobacteria;Alphaproteobacteria;Caulobacterales;Caulobacteraceae;Phenylobacterium;Phenylobacterium sp. | 0 | 0 | 6 | 0 |  |
| Bacteria;Proteobacteria;Alphaproteobacteria;Rhizobiales; | 20 | 4 | 3 | 0 |  |
| Bacteria;Proteobacteria;Alphaproteobacteria;Rhizobiales;Bradyrhizobiaceae; | 27 | 0 | 0 | 0 |  |
| Bacteria;Proteobacteria;Alphaproteobacteria;Rhizobiales;Rhizobiaceae; | 0 | 0 | 3 | 0 |  |
| Bacteria;Proteobacteria;Betaproteobacteria; | 7 | 7 | 0 | 8 |  |
| Bacteria;Proteobacteria;Betaproteobacteria;Burkholderiales;Alcaligenaceae; | 0 | 0 | 0 | 1 |  |
| Bacteria;Proteobacteria;Betaproteobacteria;Burkholderiales;Burkholderiaceae; | 0 | 0 | 0 | 1 |  |
| Bacteria;Proteobacteria;Betaproteobacteria;Burkholderiales;Burkholderiaceae;Burkholderia;Burkholderia cepacia | 0 | 0 | 0 | 0 |  |
| Bacteria;Proteobacteria;Betaproteobacteria;Nitrosomonadales; | 3 | 0 | 0 | 2 |  |
| Bacteria;Proteobacteria;Betaproteobacteria;Nitrosomonadales;Thiobacillaceae;Thiobacillus; | 0 | 0 | 0 | 8 |  |
| Bacteria;Proteobacteria;Betaproteobacteria;Rhodocyclales;Zoogloeaceae; | 0 | 0 | 0 | 1 |  |
| Bacteria;Proteobacteria;Gammaproteobacteria; | 0 | 0 | 0 | 1 |  |
| Bacteria;Proteobacteria;Gammaproteobacteria;Pseudomonadales;Pseudomonadaceae;Pseudomonas; | 13 | 0 | 0 | 0 |  |
| Bacteria;Proteobacteria;Gammaproteobacteria;Pseudomonadales;Pseudomonadaceae;Pseudomonas;Pseudomonas veronii; | 3 | 0 | 12 | 0 |  |
| Bacteria;Proteobacteria;Gammaproteobacteria;Pseudomonadales;Pseudomonadaceae;Pseudomonas;Pseudomonas syringae group; | 3 | 0 | 3 | 0 |  |
| Bacteria;Proteobacteria;Gammaproteobacteria;Xanthomonadales; | 10 | 0 | 0 | 2 |  |
| Bacteria;Proteobacteria;Hydrogenophilalia;Hydrogenophilales; | 0 | 7 | 0 | 4 |  |
| Bacteria;Proteobacteria;Hydrogenophilalia;Hydrogenophilales;unclassified Hydrogenophilales;Hydrogenophilales sp. | 0 | 0 | 3 | 1 |  |
| Bacteria;Actinobacteria;Actinobacteria; | 166 | 90 | 151 | 9 |  |
| Bacteria;Actinobacteria;Actinobacteria;Corynebacteriales;Nocardiaceae;Rhodococcus; | 13 | 0 | 0 | 0 |  |
| Bacteria;Actinobacteria;Actinobacteria;Micrococcales; | 57 | 104 | 355 | 16 |  |
| Bacteria;Actinobacteria;Actinobacteria;Micrococcales;Microbacteriaceae; | 10 | 0 | 29 | 3 |  |
| Bacteria;Actinobacteria;Actinobacteria;Micrococcales;Microbacteriaceae;Cryobacterium;Cryobacterium mesophilum; | 0 | 7 | 6 | 6 |  |
| Bacteria;Actinobacteria;Actinobacteria;Micrococcales;Microbacteriaceae;Frigoribacterium;Frigoribacterium sp. | 0 | 0 | 3 | 0 |  |
| Bacteria;Actinobacteria;Actinobacteria;Propionibacteriales;Propionibacteriaceae; | 37 | 0 | 0 | 0 |  |
| Bacteria;Actinobacteria;Actinobacteria;Propionibacteriales;Propionibacteriaceae;Cutibacterium;Cutibacterium acnes; | 3 | 0 | 0 | 0 |  |
| Bacteria;Firmicutes;Bacilli;Lactobacillales;Leuconostocaceae;Leuconostoc; | 7 | 0 | 0 | 0 |  |
| Bacteria;Firmicutes;Bacilli;Lactobacillales;Leuconostocaceae;Leuconostoc;Leuconostoc pseudomesenteroides; | 7 | 0 | 0 | 0 |  |
|  |  |  |  |  |  |
| **Cold acclimation proteins - *clp*** |  |  |  |  | 1 |
| Bacteria; | 213 | 158 | 12 | 119 |  |
| Bacteria;Actinobacteria;Actinobacteria; | 20 | 18 | 6 | 18 |  |
| Bacteria;Actinobacteria;Actinobacteria;Corynebacteriales; | 10 | 68 | 0 | 0 |  |
| Bacteria;Actinobacteria;Actinobacteria;Corynebacteriales;Nocardiaceae; | 13 | 14 | 0 | 0 |  |
| Bacteria;Actinobacteria;Actinobacteria;Corynebacteriales;Nocardiaceae;Rhodococcus; | 57 | 18 | 0 | 0 |  |
| Bacteria;Actinobacteria;Actinobacteria;Corynebacteriales;unclassified Corynebacteriales;Lawsonella clevelandensis | 17 | 0 | 0 | 0 |  |
| Bacteria;Actinobacteria;Actinobacteria;Micrococcales; | 0 | 0 | 23 | 6 |  |
| Bacteria;Actinobacteria;Actinobacteria;Micrococcales;Microbacteriaceae; | 0 | 0 | 6 | 3 |  |
| Bacteria;Actinobacteria;Actinobacteria;Micrococcales;Microbacteriaceae;Cryobacterium;Cryobacterium mesophilum; | 0 | 0 | 0 | 2 |  |
| Bacteria;Actinobacteria;Actinobacteria;Micrococcales;Microbacteriaceae;Leifsonia; | 0 | 4 | 0 | 0 |  |
| Bacteria;Actinobacteria;Actinobacteria;Propionibacteriales;Propionibacteriaceae; | 137 | 0 | 0 | 0 |  |
| Bacteria;FCB group;Bacteroidetes/Chlorobi group;Bacteroidetes; | 93 | 237 | 0 | 36 |  |
| Bacteria;FCB group;Bacteroidetes/Chlorobi group;Bacteroidetes;Sphingobacteriia;Sphingobacteriales; | 7 | 43 | 12 | 10 |  |
| Bacteria;FCB group;Bacteroidetes/Chlorobi group;Bacteroidetes;Sphingobacteriia;Sphingobacteriales;Sphingobacteriaceae; | 80 | 345 | 0 | 121 |  |
| Bacteria;FCB group;Bacteroidetes/Chlorobi group;Bacteroidetes;Sphingobacteriia;Sphingobacteriales;Sphingobacteriaceae;Pedobacter; | 0 | 7 | 12 | 7 |  |
| Bacteria;Firmicutes; | 0 | 4 | 0 | 0 |  |
| Bacteria;Fusobacteria;Fusobacteriia;Fusobacteriales;Leptotrichiaceae;Leptotrichia; | 27 | 0 | 0 | 0 |  |
| Bacteria;Proteobacteria; | 1978 | 3435 | 1410 | 2314 |  |
| Bacteria;Proteobacteria;Alphaproteobacteria; | 133 | 115 | 17 | 4 |  |
| Bacteria;Proteobacteria;Alphaproteobacteria;Caulobacterales; | 13 | 4 | 0 | 0 |  |
| Bacteria;Proteobacteria;Alphaproteobacteria;Caulobacterales;Caulobacteraceae; | 0 | 11 | 3 | 0 |  |
| Bacteria;Proteobacteria;Alphaproteobacteria;Caulobacterales;Caulobacteraceae;Caulobacter; | 0 | 4 | 0 | 0 |  |
| Bacteria;Proteobacteria;Alphaproteobacteria;Caulobacterales;Caulobacteraceae;Phenylobacterium; | 0 | 14 | 6 | 0 |  |
| Bacteria;Proteobacteria;Alphaproteobacteria;Rhizobiales; | 110 | 107 | 9 | 5 |  |
| Bacteria;Proteobacteria;Alphaproteobacteria;Rhizobiales;Bradyrhizobiaceae; | 0 | 29 | 12 | 2 |  |
| Bacteria;Proteobacteria;Alphaproteobacteria;Rhizobiales;Bradyrhizobiaceae;Bradyrhizobium; | 0 | 0 | 0 | 3 |  |
| Bacteria;Proteobacteria;Alphaproteobacteria;Rhizobiales;Phyllobacteriaceae; | 13 | 29 | 0 | 0 |  |
| Bacteria;Proteobacteria;Alphaproteobacteria;Rhizobiales;Phyllobacteriaceae;Mesorhizobium; | 3 | 0 | 0 | 0 |  |
| Bacteria;Proteobacteria;Alphaproteobacteria;Rhizobiales;unclassified Rhizobiales;unclassified Rhizobiales;Rhizobiales sp. | 0 | 0 | 6 | 0 |  |
| Bacteria;Proteobacteria;Alphaproteobacteria;Sphingomonadales; | 13 | 0 | 0 | 0 |  |
| Bacteria;Proteobacteria;Alphaproteobacteria;Sphingomonadales;Sphingomonadaceae; | 7 | 0 | 0 | 0 |  |
| Bacteria;Proteobacteria;Alphaproteobacteria;Sphingomonadales;Sphingomonadaceae;Sphingopyxis; | 3 | 0 | 0 | 0 |  |
| Bacteria;Proteobacteria;Alphaproteobacteria;unclassified Alphaproteobacteria;unclassified Alphaproteobacteria;Alphaproteobacteria sp. | 3 | 0 | 0 | 0 |  |
| Bacteria;Proteobacteria;Betaproteobacteria; | 133 | 104 | 94 | 68 |  |
| Bacteria;Proteobacteria;Betaproteobacteria;Burkholderiales; | 47 | 43 | 35 | 17 |  |
| Bacteria;Proteobacteria;Betaproteobacteria;Burkholderiales;Alcaligenaceae; | 0 | 0 | 0 | 1 |  |
| Bacteria;Proteobacteria;Betaproteobacteria;Burkholderiales;Alcaligenaceae;Achromobacter; | 0 | 0 | 0 | 2 |  |
| Bacteria;Proteobacteria;Betaproteobacteria;Burkholderiales;Burkholderiaceae; | 0 | 0 | 0 | 5 |  |
| Bacteria;Proteobacteria;Betaproteobacteria;Burkholderiales;Burkholderiaceae;Burkholderia; | 13 | 0 | 12 | 0 |  |
| Bacteria;Proteobacteria;Betaproteobacteria;Burkholderiales;Comamonadaceae; | 20 | 7 | 0 | 4 |  |
| Bacteria;Proteobacteria;Betaproteobacteria;Burkholderiales;Comamonadaceae;Hydrogenophaga; | 0 | 0 | 3 | 0 |  |
| Bacteria;Proteobacteria;Betaproteobacteria;Burkholderiales;Comamonadaceae;Hydrogenophaga;Hydrogenophaga palleronii; | 0 | 0 | 3 | 0 |  |
| Bacteria;Proteobacteria;Betaproteobacteria;Burkholderiales;Oxalobacteraceae; | 0 | 0 | 0 | 5 |  |
| Bacteria;Proteobacteria;Betaproteobacteria;Burkholderiales;Oxalobacteraceae;Herbaspirillum; | 0 | 0 | 0 | 1 |  |
| Bacteria;Proteobacteria;Betaproteobacteria;Burkholderiales;Oxalobacteraceae;Noviherbaspirillum;Noviherbaspirillum autotrophicum; | 0 | 0 | 0 | 2 |  |
| Bacteria;Proteobacteria;Betaproteobacteria;Neisseriales;Chromobacteriaceae;Aquaspirillum;Aquaspirillum sp. LM1; | 0 | 0 | 0 | 1 |  |
| Bacteria;Proteobacteria;Betaproteobacteria;Neisseriales;Chromobacteriaceae;Microvirgula;Microvirgula aerodenitrificans; | 3 | 0 | 0 | 0 |  |
| Bacteria;Proteobacteria;Betaproteobacteria;Neisseriales;Neisseriaceae; | 0 | 0 | 0 | 1 |  |
| Bacteria;Proteobacteria;Betaproteobacteria;Nitrosomonadales; | 0 | 0 | 3 | 0 |  |
| Bacteria;Proteobacteria;Betaproteobacteria;Nitrosomonadales;Thiobacillaceae;Thiobacillus; | 3 | 54 | 0 | 46 |  |
| Bacteria;Proteobacteria;Betaproteobacteria;Nitrosomonadales;Thiobacillaceae;Thiobacillus;Thiobacillus thioparus; | 0 | 8 | 0 | 2 |  |
| Bacteria;Proteobacteria;delta/epsilon subdivisions;Epsilonproteobacteria;Campylobacterales;Campylobacteraceae;Campylobacter; | 3 | 0 | 0 | 0 |  |
| Bacteria;Proteobacteria;Gammaproteobacteria; | 10 | 0 | 12 | 7 |  |
| Bacteria;Proteobacteria;Gammaproteobacteria;Alteromonadales; | 0 | 7 | 0 | 0 |  |
| Bacteria;Proteobacteria;Gammaproteobacteria;Enterobacterales;Enterobacteriaceae;Enterobacter;Enterobacter cloacae | 0 | 0 | 0 | 1 |  |
| Bacteria;Proteobacteria;Gammaproteobacteria;Oceanospirillales;Halomonadaceae;unclassified Halomonadaceae;Halomonadaceae sp. | 7 | 0 | 0 | 0 |  |
| Bacteria;Proteobacteria;Gammaproteobacteria;Pseudomonadales; | 0 | 0 | 6 | 0 |  |
| Bacteria;Proteobacteria;Gammaproteobacteria;Pseudomonadales;Pseudomonadaceae; | 7 | 14 | 0 | 0 |  |
| Bacteria;Proteobacteria;Gammaproteobacteria;Pseudomonadales;Pseudomonadaceae;Pseudomonas; | 100 | 21 | 50 | 6 |  |
| Bacteria;Proteobacteria;Gammaproteobacteria;Xanthomonadales; | 7 | 51 | 0 | 4 |  |
| Bacteria;Proteobacteria;Gammaproteobacteria;Xanthomonadales;Rhodanobacteraceae; | 7 | 0 | 0 | 3 |  |
| Bacteria;Proteobacteria;Gammaproteobacteria;Xanthomonadales;Rhodanobacteraceae;Rhodanobacter; | 10 | 15 | 0 | 3 |  |
| Bacteria;Proteobacteria;Gammaproteobacteria;Xanthomonadales;Xanthomonadaceae; | 13 | 0 | 0 | 4 |  |
| Bacteria;Proteobacteria;Gammaproteobacteria;Xanthomonadales;Xanthomonadaceae;Lysobacter; | 0 | 0 | 0 | 1 |  |
| Bacteria;Proteobacteria;Gammaproteobacteria;Xanthomonadales;Xanthomonadaceae;Pseudoxanthomonas sp. | 0 | 0 | 0 | 1 |  |
| Bacteria;Proteobacteria;Hydrogenophilalia;Hydrogenophilales;unclassified Hydrogenophilales;Hydrogenophilales bacterium | 57 | 14 | 20 | 14 |  |
|  |  |  |  |  |  |
| **Chaperones - Trigger factor *tig*** |  |  |  |  | 0.94 |
| Bacteria; | 6 | 4 | 32 | 8 |  |
| Bacteria;Actinobacteria;Actinobacteria; | 7 | 18 | 23 | 20 |  |
| Bacteria;Actinobacteria;Actinobacteria;Corynebacteriales;Nocardiaceae;Rhodococcus; | 0 | 0 | 0 | 2 |  |
| Bacteria;Actinobacteria;Actinobacteria;Micrococcales; | 0 | 0 | 15 | 2 |  |
| Bacteria;Actinobacteria;Actinobacteria;Micrococcales;Microbacteriaceae; | 53 | 32 | 79 | 26 |  |
| Bacteria;Actinobacteria;Actinobacteria;Micrococcales;Microbacteriaceae;Cryobacterium; | 0 | 0 | 0 | 1 |  |
| Bacteria;Actinobacteria;Actinobacteria;Micrococcales;Microbacteriaceae;Cryobacterium;Cryobacterium mesophilum; | 0 | 18 | 38 | 32 |  |
| Bacteria;Actinobacteria;Actinobacteria;Micrococcales;Microbacteriaceae;Leifsonia; | 0 | 0 | 3 | 0 |  |
| Bacteria;Actinobacteria;Actinobacteria;Micrococcales;Micrococcaceae; | 13 | 0 | 0 | 0 |  |
| Bacteria;Actinobacteria;Actinobacteria;Propionibacteriales;Propionibacteriaceae; | 117 | 0 | 0 | 0 |  |
| Bacteria;Firmicutes;Bacilli;Bacillales;Staphylococcaceae;Staphylococcus; | 3 | 0 | 0 | 0 |  |
| Bacteria;Firmicutes;Bacilli;Lactobacillales;Streptococcaceae;Streptococcus; | 3 | 0 | 0 | 0 |  |
| Bacteria;Proteobacteria; | 1478 | 5124 | 797 | 1177 |  |
| Bacteria;Proteobacteria;Alphaproteobacteria; | 13 | 0 | 0 | 0 |  |
| Bacteria;Proteobacteria;Alphaproteobacteria;Rhizobiales; | 20 | 29 | 0 | 0 |  |
| Bacteria;Proteobacteria;Alphaproteobacteria;Rhizobiales;Methylobacteriaceae;Methylobacterium; | 13 | 0 | 0 | 0 |  |
| Bacteria;Proteobacteria;Alphaproteobacteria;Rhizobiales;Phyllobacteriaceae; | 7 | 0 | 0 | 0 |  |
| Bacteria;Proteobacteria;Alphaproteobacteria;Rhizobiales;Phyllobacteriaceae;Mesorhizobium; | 7 | 0 | 0 | 0 |  |
| Bacteria;Proteobacteria;Alphaproteobacteria;Sphingomonadales;Sphingomonadaceae;Sphingomonas; | 13 | 0 | 0 | 0 |  |
| Bacteria;Proteobacteria;Betaproteobacteria; | 7 | 0 | 0 | 2 |  |
| Bacteria;Proteobacteria;Betaproteobacteria;Burkholderiales; | 13 | 0 | 0 | 3 |  |
| Bacteria;Proteobacteria;Betaproteobacteria;Nitrosomonadales;Thiobacillaceae;Thiobacillus; | 0 | 25 | 0 | 10 |  |
| Bacteria;Proteobacteria;Betaproteobacteria;Nitrosomonadales;Thiobacillaceae;Thiobacillus;Thiobacillus denitrificans; | 0 | 7 | 0 | 1 |  |
| Bacteria;Proteobacteria;Gammaproteobacteria;Xanthomonadales; | 0 | 0 | 0 | 2 |  |
| Bacteria;Proteobacteria;Gammaproteobacteria;Xanthomonadales;Rhodanobacteraceae;Rhodanobacter; | 10 | 0 | 0 | 0 |  |
| Bacteria;Proteobacteria;Gammaproteobacteria;Xanthomonadales;Xanthomonadaceae; | 0 | 0 | 0 | 2 |  |
| Bacteria;Proteobacteria;Gammaproteobacteria;Xanthomonadales;Xanthomonadaceae;Pseudoxanthomonas; | 0 | 0 | 0 | 1 |  |
| Bacteria;Proteobacteria;Gammaproteobacteria;Xanthomonadales;Xanthomonadaceae;Xanthomonas; | 0 | 7 | 0 | 2 |  |
| Bacteria;Proteobacteria;Hydrogenophilalia;Hydrogenophilales;unclassified Hydrogenophilales;Hydrogenophilales bacterium | 3 | 4 | 0 | 0 |  |
|  |  |  |  |  |  |
| **Chaperones - protein export chaperone *secB*** |  |  |  |  | 1 |
| Bacteria; | 7 | 0 | 3 | 1 |  |
| Bacteria;Proteobacteria; | 100 | 158 | 47 | 130 |  |
| Bacteria;Proteobacteria;Alphaproteobacteria;Rhizobiales;unclassified Rhizobiales;unclassified Rhizobiales; | 3 | 0 | 0 | 0 |  |
| Bacteria;Proteobacteria;Alphaproteobacteria;Sphingomonadales;Sphingomonadaceae;Sphingomonas;Sphingomonas sanguinis; | 3 | 0 | 0 | 0 |  |
| Bacteria;Proteobacteria;Betaproteobacteria; | 0 | 0 | 0 | 1 |  |
| Bacteria;Proteobacteria;Betaproteobacteria;Burkholderiales;Comamonadaceae; | 40 | 0 | 0 | 0 |  |
| Bacteria;Proteobacteria;Betaproteobacteria;Nitrosomonadales;Thiobacillaceae;Thiobacillus; | 0 | 11 | 0 | 7 |  |
| Bacteria;Proteobacteria;Betaproteobacteria;Nitrosomonadales;Thiobacillaceae;Thiobacillus;Thiobacillus denitrificans; | 0 | 7 | 0 | 9 |  |
| Bacteria;Proteobacteria;Gammaproteobacteria;Pseudomonadales;Pseudomonadaceae;Pseudomonas; | 7 | 0 | 0 | 0 |  |
| Bacteria;Proteobacteria;Gammaproteobacteria;Xanthomonadales;Xanthomonadaceae; | 0 | 0 | 0 | 1 |  |
| Bacteria;Proteobacteria;Gammaproteobacteria;Xanthomonadales;Xanthomonadaceae;Pseudoxanthomonas; | 3 | 0 | 0 | 1 |  |
| Bacteria;Actinobacteria;Actinobacteria;Propionibacteriales;Propionibacteriaceae; | 3 | 0 | 0 | 0 |  |
|  |  |  |  |  |  |
| **Transcription/translation - transcription termination/antitermination L factor *nusA*** |  |  |  |  | 1 |
| Bacteria; | 37 | 11 | 26 | 9 |  |
| Bacteria;Actinobacteria; | 27 | 0 | 0 | 0 |  |
| Bacteria;Actinobacteria;Actinobacteria; | 7 | 0 | 47 | 12 |  |
| Bacteria;Actinobacteria;Actinobacteria;Micrococcales; | 0 | 0 | 0 | 3 |  |
| Bacteria;Actinobacteria;Actinobacteria;Micrococcales;Microbacteriaceae; | 3 | 0 | 90 | 12 |  |
| Bacteria;Actinobacteria;Actinobacteria;Micrococcales;Microbacteriaceae;Cryobacterium;Cryobacterium mesophilum; | 0 | 0 | 70 | 5 |  |
| Bacteria;Actinobacteria;Actinobacteria;Micrococcales;Micrococcaceae; | 7 | 0 | 0 | 0 |  |
| Bacteria;Actinobacteria;Actinobacteria;Micrococcales;Micrococcaceae;Kocuria; | 7 | 0 | 0 | 0 |  |
| Bacteria;Actinobacteria;Actinobacteria;Micrococcales;Micrococcaceae;Rothia; | 0 | 7 | 0 | 0 |  |
| Bacteria;Actinobacteria;Actinobacteria;Propionibacteriales; | 40 | 0 | 0 | 0 |  |
| Bacteria;Actinobacteria;Actinobacteria;Propionibacteriales;Propionibacteriaceae; | 20 | 0 | 0 | 0 |  |
| Bacteria;Actinobacteria;Actinobacteria;Propionibacteriales;Propionibacteriaceae;Cutibacterium; | 23 | 0 | 0 | 0 |  |
| Bacteria;Bacteroidetes/Chlorobi group;Bacteroidetes;Sphingobacteriia;Sphingobacteriales;Sphingobacteriaceae;Pedobacter; | 0 | 0 | 0 | 2 |  |
| Bacteria;Firmicutes;Bacilli;Bacillales;Staphylococcaceae;Staphylococcus; | 13 | 0 | 0 | 0 |  |
| Bacteria;Firmicutes;Bacilli;Lactobacillales;Streptococcaceae;Streptococcus; | 0 | 4 | 0 | 0 |  |
| Bacteria;Proteobacteria; | 293 | 1365 | 302 | 520 |  |
| Bacteria;Proteobacteria;Alphaproteobacteria; | 7 | 14 | 12 | 0 |  |
| Bacteria;Proteobacteria;Alphaproteobacteria;Rhizobiales; | 7 | 0 | 0 | 0 |  |
| Bacteria;Proteobacteria;Alphaproteobacteria;Rhizobiales;Phyllobacteriaceae; | 0 | 7 | 0 | 0 |  |
| Bacteria;Proteobacteria;Alphaproteobacteria;Rhizobiales;Phyllobacteriaceae;Mesorhizobium; | 13 | 7 | 0 | 0 |  |
| Bacteria;Proteobacteria;Alphaproteobacteria;Rhizobiales;Rhizobiaceae;Rhizobium/Agrobacterium group; | 0 | 0 | 0 | 1 |  |
| Bacteria;Proteobacteria;Alphaproteobacteria;Sphingomonadales;Sphingomonadaceae; | 0 | 7 | 0 | 0 |  |
| Bacteria;Proteobacteria;Betaproteobacteria; | 10 | 0 | 3 | 5 |  |
| Bacteria;Proteobacteria;Betaproteobacteria;Burkholderiales; | 10 | 4 | 0 | 7 |  |
| Bacteria;Proteobacteria;Betaproteobacteria;Burkholderiales;Burkholderiaceae; | 10 | 0 | 17 | 3 |  |
| Bacteria;Proteobacteria;Betaproteobacteria;Burkholderiales;Comamonadaceae; | 0 | 0 | 0 | 1 |  |
| Bacteria;Proteobacteria;Betaproteobacteria;Neisseriales;Neisseriaceae; | 13 | 0 | 0 | 0 |  |
| Bacteria;Proteobacteria;Betaproteobacteria;Nitrosomonadales;Thiobacillaceae;Thiobacillus; | 0 | 7 | 0 | 4 |  |
| Bacteria;Proteobacteria;Gammaproteobacteria; | 0 | 0 | 6 | 0 |  |
| Bacteria;Proteobacteria;Gammaproteobacteria;Enterobacterales;Enterobacteriaceae;Klebsiella;Klebsiella oxytoca; | 0 | 4 | 0 | 6 |  |
| Bacteria;Proteobacteria;Gammaproteobacteria;Pseudomonadales;Moraxellaceae; | 27 | 0 | 0 | 0 |  |
| Bacteria;Proteobacteria;Gammaproteobacteria;Pseudomonadales;Pseudomonadaceae;Pseudomonas; | 0 | 0 | 17 | 0 |  |
| Bacteria;Proteobacteria;Gammaproteobacteria;Xanthomonadales; | 7 | 7 | 0 | 0 |  |
| Bacteria;Proteobacteria;Gammaproteobacteria;Xanthomonadales;Rhodanobacteraceae; | 3 | 7 | 0 | 0 |  |
| Bacteria;Proteobacteria;Gammaproteobacteria;Xanthomonadales;Xanthomonadaceae; | 0 | 0 | 3 | 0 |  |
| Bacteria;Proteobacteria;Gammaproteobacteria;Xanthomonadales;Xanthomonadaceae;Pseudoxanthomonas; | 7 | 0 | 0 | 0 |  |
| Bacteria;Proteobacteria;Hydrogenophilalia;Hydrogenophilales;unclassified Hydrogenophilales;Hydrogenophilales bacterium | 3 | 0 | 0 | 0 |  |
|  |  |  |  |  |  |
| **Transcription/translation - polyribonucleotide nucleotidyltransferase** |  |  |  |  | 1 |
| Bacteria; | 73 | 43 | 84 | 5 |  |
| Bacteria;Proteobacteria;Betaproteobacteria;Nitrosomonadales;Thiobacillaceae;Thiobacillus; | 0 | 0 | 0 | 1 |  |
| Bacteria; | 0 | 0 | 0 | 0 |  |
| Bacteria;Actinobacteria; | 0 | 11 | 6 | 0 |  |
| Bacteria;Actinobacteria;Actinobacteria; | 97 | 101 | 209 | 41 |  |
| Bacteria;Actinobacteria;Actinobacteria;Corynebacteriales; | 0 | 7 | 0 | 0 |  |
| Bacteria;Actinobacteria;Actinobacteria;Corynebacteriales;Nocardiaceae;Rhodococcus; | 0 | 7 | 0 | 0 |  |
| Bacteria;Actinobacteria;Actinobacteria;Micrococcales; | 27 | 75 | 29 | 17 |  |
| Bacteria;Actinobacteria;Actinobacteria;Micrococcales;Microbacteriaceae; | 120 | 72 | 241 | 48 |  |
| Bacteria;Actinobacteria;Actinobacteria;Micrococcales;Microbacteriaceae;Cryobacterium; | 0 | 0 | 0 | 0 |  |
| Bacteria;Actinobacteria;Actinobacteria;Micrococcales;Microbacteriaceae;Cryobacterium;Cryobacterium mesophilum; | 13 | 0 | 17 | 3 |  |
| Bacteria;Actinobacteria;Actinobacteria;Micrococcales;Microbacteriaceae;Curtobacterium; | 0 | 0 | 6 | 0 |  |
| Bacteria;Actinobacteria;Actinobacteria;Micrococcales;Microbacteriaceae;Leucobacter;Leucobacter sp. Ag1; | 0 | 0 | 6 | 0 |  |
| Bacteria;Actinobacteria;Actinobacteria;Propionibacteriales;Propionibacteriaceae; | 73 | 0 | 0 | 0 |  |
| Bacteria;Actinobacteria;Actinobacteria;Propionibacteriales;Propionibacteriaceae;Cutibacterium; | 13 | 0 | 0 | 0 |  |
| Bacteria;Actinobacteria;Actinobacteria;Propionibacteriales;Propionibacteriaceae;Cutibacterium;Propionibacterium acnes | 3 | 0 | 0 | 0 |  |
| Bacteria;Firmicutes;Bacilli;Bacillales;Staphylococcaceae;Staphylococcus;Staphylococcus aureus; | 3 | 0 | 0 | 0 |  |
|  |  |  |  |  |  |
| **Transcription/translation - polynucleotide phosphorylase *pnp*** |  |  |  |  | 0.94 |
| Bacteria; | 0 | 0 | 0 | 1 |  |
| Bacteria;Proteobacteria;Alphaproteobacteria;Rhizobiales; | 0 | 0 | 3 | 0 |  |
| Bacteria;Proteobacteria;Alphaproteobacteria;Rhizobiales;Rhizobiaceae;Rhizobium/Agrobacterium group; | 0 | 0 | 3 | 0 |  |
| Bacteria;Actinobacteria; | 0 | 0 | 12 | 2 |  |
| Bacteria;Actinobacteria;Actinobacteria; | 0 | 4 | 12 | 6 |  |
| Bacteria;Actinobacteria;Actinobacteria;Micrococcales; | 0 | 0 | 0 | 2 |  |
| Bacteria;Actinobacteria;Actinobacteria;Micrococcales;Microbacteriaceae; | 0 | 14 | 64 | 20 |  |
| Bacteria;Actinobacteria;Actinobacteria;Propionibacteriales;Nocardioidaceae;Nocardioides;Nocardioides exalbidus; | 0 | 4 | 0 | 0 |  |
|  |  |  |  |  |  |
| **Transcription/translation - DNA gyrase *gyrAB*** |  |  |  |  | 1 |
| Bacteria; | 267 | 129 | 250 | 128 |  |
| Bacteria;Actinobacteria; | 7 | 14 | 0 | 3 |  |
| Bacteria;Actinobacteria;Actinobacteria; | 10 | 29 | 149 | 49 |  |
| Bacteria;Actinobacteria;Actinobacteria;Corynebacteriales; | 27 | 0 | 0 | 0 |  |
| Bacteria;Actinobacteria;Actinobacteria;Corynebacteriales;Corynebacteriaceae;Corynebacterium; | 13 | 0 | 0 | 0 |  |
| Bacteria;Actinobacteria;Actinobacteria;Micrococcales; | 37 | 36 | 105 | 31 |  |
| Bacteria;Actinobacteria;Actinobacteria;Micrococcales;Microbacteriaceae; | 43 | 119 | 268 | 109 |  |
| Bacteria;Actinobacteria;Actinobacteria;Micrococcales;Microbacteriaceae;Cryobacterium;Cryobacterium mesophilum; | 33 | 50 | 130 | 26 |  |
| Bacteria;Actinobacteria;Actinobacteria;Micrococcales;Micrococcaceae; | 7 | 0 | 0 | 0 |  |
| Bacteria;Actinobacteria;Actinobacteria;Propionibacteriales; | 13 | 0 | 0 | 0 |  |
| Bacteria;Actinobacteria;Actinobacteria;Propionibacteriales;Propionibacteriaceae; | 87 | 0 | 0 | 0 |  |
| Bacteria;Actinobacteria;Actinobacteria;Propionibacteriales;Propionibacteriaceae;Cutibacterium; | 160 | 0 | 0 | 0 |  |
| Bacteria;Actinobacteria;Actinobacteria;Propionibacteriales;Propionibacteriaceae;Cutibacterium;Cutibacterium acnes; | 30 | 0 | 0 | 0 |  |
| Bacteria;Actinobacteria;Actinobacteria;Propionibacteriales;Propionibacteriaceae;Cutibacterium;Propionibacterium acnes | 7 | 0 | 0 | 0 |  |
| Bacteria;Actinobacteria;Actinobacteria;Pseudonocardiales;Pseudonocardiaceae;Saccharopolyspora; | 13 | 0 | 0 | 0 |  |
| Bacteria;Actinobacteria;Actinobacteria;unclassified Actinobacteria (class);unclassified Actinobacteria (class);actinobacterium sp. | 0 | 0 | 0 | 1 |  |
| Bacteria;FCB group;Bacteroidetes/Chlorobi group;Bacteroidetes; | 0 | 0 | 0 | 5 |  |
| Bacteria;FCB group;Bacteroidetes/Chlorobi group;Bacteroidetes;Bacteroidia;Bacteroidales;Prevotellaceae; | 27 | 0 | 0 | 0 |  |
| Bacteria;FCB group;Bacteroidetes/Chlorobi group;Bacteroidetes;Flavobacteriia;Flavobacteriales;Flavobacteriaceae; | 0 | 0 | 6 | 0 |  |
| Bacteria;FCB group;Bacteroidetes/Chlorobi group;Bacteroidetes;Sphingobacteriia;Sphingobacteriales;Sphingobacteriaceae; | 13 | 50 | 0 | 22 |  |
| Bacteria;FCB group;Bacteroidetes/Chlorobi group;Bacteroidetes;Sphingobacteriia;Sphingobacteriales;Sphingobacteriaceae;Pedobacter; | 0 | 7 | 6 | 1 |  |
| Bacteria;Firmicutes;Bacilli;Bacillales; | 0 | 0 | 6 | 0 |  |
| Bacteria;Firmicutes;Bacilli;Bacillales;Bacillales incertae sedis;Bacillales Family XI. Incertae Sedis;Gemella; | 0 | 7 | 0 | 0 |  |
| Bacteria;Firmicutes;Bacilli;Bacillales;Staphylococcaceae;Staphylococcus; | 3 | 0 | 0 | 0 |  |
| Bacteria;Firmicutes;Bacilli;Lactobacillales;Aerococcaceae;Abiotrophia; | 0 | 14 | 0 | 0 |  |
| Bacteria;Firmicutes;Bacilli;Lactobacillales;Streptococcaceae; | 3 | 0 | 0 | 0 |  |
| Bacteria;Firmicutes;Bacilli;Lactobacillales;Streptococcaceae;Streptococcus; | 13 | 0 | 0 | 0 |  |
| Bacteria;Proteobacteria; | 516 | 1858 | 596 | 2006 |  |
| Bacteria;Proteobacteria;Alphaproteobacteria; | 3 | 0 | 17 | 1 |  |
| Bacteria;Proteobacteria;Alphaproteobacteria;Rhizobiales; | 70 | 4 | 0 | 0 |  |
| Bacteria;Proteobacteria;Alphaproteobacteria;Rhizobiales;Phyllobacteriaceae; | 0 | 43 | 0 | 0 |  |
| Bacteria;Proteobacteria;Alphaproteobacteria;Rhizobiales;Phyllobacteriaceae;Mesorhizobium; | 54 | 0 | 0 | 0 |  |
| Bacteria;Proteobacteria;Alphaproteobacteria;Rhizobiales;Rhizobiaceae;Rhizobium/Agrobacterium group; | 13 | 0 | 0 | 0 |  |
| Bacteria;Proteobacteria;Alphaproteobacteria;Rhodobacterales;Rhodobacteraceae; | 13 | 0 | 0 | 1 |  |
| Bacteria;Proteobacteria;Alphaproteobacteria;Sphingomonadales;Sphingomonadaceae; | 7 | 0 | 0 | 0 |  |
| Bacteria;Proteobacteria;Alphaproteobacteria;Sphingomonadales;Sphingomonadaceae;Sphingomonas; | 13 | 0 | 6 | 2 |  |
| Bacteria;Proteobacteria;Betaproteobacteria; | 10 | 18 | 26 | 18 |  |
| Bacteria;Proteobacteria;Betaproteobacteria;Burkholderiales; | 20 | 0 | 0 | 7 |  |
| Bacteria;Proteobacteria;Betaproteobacteria;Burkholderiales;Burkholderiaceae; | 20 | 14 | 12 | 0 |  |
| Bacteria;Proteobacteria;Betaproteobacteria;Burkholderiales;Burkholderiaceae;Burkholderia;Burkholderia cepacia complex; | 0 | 0 | 0 | 1 |  |
| Bacteria;Proteobacteria;Betaproteobacteria;Burkholderiales;Comamonadaceae;Comamonas;Comamonas sp. | 0 | 0 | 0 | 1 |  |
| Bacteria;Proteobacteria;Betaproteobacteria;Burkholderiales;Comamonadaceae;Pelomonas; | 3 | 0 | 0 | 0 |  |
| Bacteria;Proteobacteria;Betaproteobacteria;Burkholderiales;unclassified Burkholderiales;Aquabacterium sp. | 0 | 0 | 3 | 0 |  |
| Bacteria;Proteobacteria;Betaproteobacteria;Neisseriales; | 0 | 0 | 0 | 3 |  |
| Bacteria;Proteobacteria;Betaproteobacteria;Neisseriales;Neisseriaceae; | 0 | 0 | 0 | 5 |  |
| Bacteria;Proteobacteria;Betaproteobacteria;Nitrosomonadales;Methylophilaceae;Methylobacillus;Methylobacillus sp. | 3 | 0 | 0 | 0 |  |
| Bacteria;Proteobacteria;Betaproteobacteria;Nitrosomonadales;Thiobacillaceae;Thiobacillus; | 3 | 4 | 0 | 21 |  |
| Bacteria;Proteobacteria;Betaproteobacteria;Nitrosomonadales;Thiobacillaceae;Thiobacillus;Thiobacillus thioparus; | 0 | 0 | 0 | 3 |  |
| Bacteria;Proteobacteria;Gammaproteobacteria; | 0 | 0 | 0 | 6 |  |
| Bacteria;Proteobacteria;Gammaproteobacteria;Enterobacterales;Enterobacteriaceae; | 0 | 14 | 0 | 0 |  |
| Bacteria;Proteobacteria;Gammaproteobacteria;Pseudomonadales;Moraxellaceae;Acinetobacter; | 7 | 7 | 0 | 0 |  |
| Bacteria;Proteobacteria;Gammaproteobacteria;Pseudomonadales;Pseudomonadaceae;Pseudomonas; | 0 | 0 | 0 | 3 |  |
| Bacteria;Proteobacteria;Gammaproteobacteria;Xanthomonadales; | 0 | 14 | 0 | 8 |  |
| Bacteria;Proteobacteria;Gammaproteobacteria;Xanthomonadales;Rhodanobacteraceae; | 0 | 22 | 0 | 3 |  |
| Bacteria;Proteobacteria;Gammaproteobacteria;Xanthomonadales;Rhodanobacteraceae;Rhodanobacter; | 0 | 7 | 0 | 2 |  |
| Bacteria;Proteobacteria;Gammaproteobacteria;Xanthomonadales;Xanthomonadaceae; | 0 | 0 | 0 | 4 |  |
| Bacteria;Proteobacteria;Gammaproteobacteria;Xanthomonadales;Xanthomonadaceae;Pseudoxanthomonas; | 0 | 0 | 0 | 2 |  |
| Bacteria;Proteobacteria;Hydrogenophilalia;Hydrogenophilales;Hydrogenophilaceae;Tepidiphilus; | 13 | 0 | 0 | 0 |  |
|  |  |  |  |  |  |
| **Transcription/translation - DEAD-box RNA helicase *deaD*** |  |  |  |  | 1.00 |
| Bacteria; | 53 | 14 | 9 | 0 |  |
| Bacteria;Actinobacteria;Actinobacteria; | 7 | 0 | 0 | 0 |  |
| Bacteria;Actinobacteria;Actinobacteria; | 57 | 0 | 0 | 0 |  |
| Bacteria;Actinobacteria;Actinobacteria;Corynebacteriales; | 7 | 0 | 0 | 0 |  |
| Bacteria;Actinobacteria;Actinobacteria;Corynebacteriales;Nocardiaceae; | 13 | 0 | 0 | 0 |  |
| Bacteria;Actinobacteria;Actinobacteria;Corynebacteriales;Nocardiaceae;Rhodococcus; | 34 | 0 | 0 | 0 |  |
| Bacteria;Actinobacteria;Actinobacteria;Corynebacteriales;unclassified Corynebacteriales;Lawsonella;Lawsonella clevelandensis; | 20 | 0 | 0 | 0 |  |
| Bacteria;Actinobacteria;Actinobacteria;Micrococcales;Micrococcaceae;Rothia; | 0 | 7 | 0 | 0 |  |
| Bacteria;Actinobacteria;Actinobacteria;Micrococcales;Micrococcaceae;Rothia;Rothia aeria;Rothia aeria | 0 | 4 | 0 | 0 |  |
| Bacteria;Actinobacteria;Actinobacteria;Micromonosporales;Micromonosporaceae;Asanoa;Asanoa ferruginea; | 3 | 0 | 0 | 0 |  |
| Bacteria;Actinobacteria;Actinobacteria;Propionibacteriales;Propionibacteriaceae; | 206 | 0 | 0 | 0 |  |
| Bacteria;Actinobacteria;Actinobacteria;Propionibacteriales;Propionibacteriaceae;Cutibacterium;Cutibacterium acnes; | 7 | 0 | 0 | 0 |  |
| Bacteria;Firmicutes;Bacilli;Bacillales;Bacillales incertae sedis;Bacillales Family XI. Incertae Sedis;Gemella; | 0 | 14 | 0 | 0 |  |
| Bacteria;Firmicutes;Bacilli;Lactobacillales;Streptococcaceae;Streptococcus; | 40 | 14 | 0 | 0 |  |
| Bacteria;Proteobacteria; | 0 | 0 | 0 | 2 |  |
| Bacteria;Proteobacteria;Alphaproteobacteria;Sphingomonadales;Sphingomonadaceae;Sphingomonas; | 3 | 0 | 0 | 0 |  |
| Bacteria;Proteobacteria;Gammaproteobacteria;Chromatiales;Chromatiaceae; | 0 | 0 | 3 | 0 |  |
| Bacteria;Proteobacteria;Gammaproteobacteria;Pseudomonadales;Pseudomonadaceae;Pseudomonas; | 33 | 29 | 0 | 0 |  |
| Bacteria;Proteobacteria;Gammaproteobacteria;Pseudomonadales;Pseudomonadaceae;Pseudomonas;Pseudomonas syringae | 7 | 0 | 0 | 0 |  |
| Bacteria;Proteobacteria;Gammaproteobacteria;Xanthomonadales; | 0 | 0 | 0 | 2 |  |
| Bacteria;Proteobacteria;Gammaproteobacteria;Xanthomonadales;Rhodanobacteraceae;Rhodanobacter;Rhodanobacter sp. | 0 | 0 | 0 | 1 |  |
| Bacteria;Proteobacteria;Gammaproteobacteria;Xanthomonadales;Xanthomonadaceae; | 13 | 0 | 0 | 2 |  |
| Bacteria;Proteobacteria;Gammaproteobacteria;Xanthomonadales;Xanthomonadaceae;Pseudoxanthomonas; | 7 | 0 | 0 | 0 |  |
| Bacteria;Proteobacteria;Hydrogenophilalia;Hydrogenophilales;unclassified Hydrogenophilales;Hydrogenophilales sp. | 0 | 0 | 0 | 0 |  |
|  |  |  |  |  |  |
| **Membrane adaptations - N-acetylglucosaminyl transferase *icaA*** |  |  |  |  | 1 |
| Bacteria; | 56 | 29 | 32 | 23 |  |
| Bacteria;Actinobacteria; | 0 | 0 | 0 | 2 |  |
| Bacteria;Actinobacteria;Actinobacteria; | 0 | 0 | 0 | 11 |  |
| Bacteria;Actinobacteria;Actinobacteria;Corynebacteriales;Corynebacteriaceae;Corynebacterium; | 13 | 0 | 0 | 0 |  |
| Bacteria;Actinobacteria;Actinobacteria;Micrococcales; | 0 | 0 | 3 | 6 |  |
| Bacteria;Actinobacteria;Actinobacteria;Micrococcales;Microbacteriaceae; | 0 | 0 | 9 | 129 |  |
| Bacteria;Actinobacteria;Actinobacteria;Micrococcales;Microbacteriaceae;Cryobacterium; | 0 | 0 | 0 | 2 |  |
| Bacteria;Actinobacteria;Actinobacteria;Micrococcales;Microbacteriaceae;Cryobacterium;Cryobacterium mesophilum; | 0 | 0 | 0 | 23 |  |
| Bacteria;Actinobacteria;Actinobacteria;Micrococcales;Microbacteriaceae;Frigoribacterium; | 0 | 0 | 0 | 5 |  |
| Bacteria;Actinobacteria;Actinobacteria;Micrococcales;Microbacteriaceae;Frigoribacterium;Frigoribacterium sp. | 0 | 0 | 0 | 2 |  |
| Bacteria;Actinobacteria;Actinobacteria;Micrococcales;Microbacteriaceae;Leifsonia;Leifsonia sp. | 0 | 0 | 0 | 11 |  |
| Bacteria;Actinobacteria;Actinobacteria;Micrococcales;Microbacteriaceae;Salinibacterium;Salinibacterium xinjiangense; | 0 | 0 | 0 | 1 |  |
| Bacteria;Actinobacteria;Actinobacteria;Propionibacteriales;Propionibacteriaceae; | 17 | 7 | 0 | 0 |  |
| Bacteria;FCB group;Bacteroidetes/Chlorobi group;Bacteroidetes;Sphingobacteriia;Sphingobacteriales;Sphingobacteriaceae; | 0 | 0 | 0 | 3 |  |
| Bacteria;Firmicutes;Bacilli;Lactobacillales;Streptococcaceae;Streptococcus; | 0 | 4 | 0 | 0 |  |
| Bacteria;Proteobacteria; | 423 | 841 | 366 | 810 |  |
| Bacteria;Proteobacteria;Betaproteobacteria; | 3 | 4 | 0 | 2 |  |
| Bacteria;Proteobacteria;Betaproteobacteria;Burkholderiales; | 0 | 0 | 0 | 2 |  |
| Bacteria;Proteobacteria;Betaproteobacteria;Burkholderiales;Burkholderiaceae; | 0 | 14 | 0 | 0 |  |
| Bacteria;Proteobacteria;Betaproteobacteria;Burkholderiales;Burkholderiaceae;Ralstonia; | 0 | 0 | 6 | 0 |  |
| Bacteria;Proteobacteria;Betaproteobacteria;Burkholderiales;Comamonadaceae; | 0 | 0 | 0 | 1 |  |
| Bacteria;Proteobacteria;Betaproteobacteria;Burkholderiales;Comamonadaceae;Hydrogenophaga; | 0 | 0 | 0 | 1 |  |
| Bacteria;Proteobacteria;Betaproteobacteria;Nitrosomonadales;Gallionellaceae;Sulfuriferula;Sulfuriferula sp. | 3 | 0 | 0 | 0 |  |
| Bacteria;Proteobacteria;Betaproteobacteria;Nitrosomonadales;Thiobacillaceae;Thiobacillus; | 23 | 90 | 3 | 101 |  |
| Bacteria;Proteobacteria;Betaproteobacteria;Nitrosomonadales;Thiobacillaceae;Thiobacillus;Thiobacillus denitrificans; | 7 | 4 | 3 | 1 |  |
| Bacteria;Proteobacteria;Betaproteobacteria;Nitrosomonadales;Thiobacillaceae;Thiobacillus;Thiobacillus thioparus; | 3 | 18 | 0 | 6 |  |
| Bacteria;Proteobacteria;Betaproteobacteria;unclassified Betaproteobacteria;unclassified Betaproteobacteria; | 3 | 0 | 0 | 0 |  |
| Bacteria;Proteobacteria;Gammaproteobacteria;Pseudomonadales;Moraxellaceae;Acinetobacter; | 13 | 0 | 0 | 0 |  |
| Bacteria;Proteobacteria;Gammaproteobacteria;Xanthomonadales;Rhodanobacteraceae; | 0 | 0 | 0 | 1 |  |
| Bacteria;Proteobacteria;Gammaproteobacteria;Xanthomonadales;Rhodanobacteraceae;Rhodanobacter; | 0 | 0 | 0 | 1 |  |
| Bacteria;Proteobacteria;Hydrogenophilalia;Hydrogenophilales;unclassified Hydrogenophilales; | 3 | 0 | 0 | 0 |  |
| Bacteria;Proteobacteria;Hydrogenophilalia;Hydrogenophilales;unclassified Hydrogenophilales;Hydrogenophilales sp. | 13 | 0 | 0 | 1 |  |
|  |  |  |  |  |  |
| **Membrane adaptations - outer membrane porin *pgaABC*** |  |  |  |  | 1 |
| Bacteria;Proteobacteria; | 0 | 0 | 0 | 2 |  |
| Bacteria;Proteobacteria;Betaproteobacteria;Burkholderiales;Comamonadaceae; | 0 | 0 | 0 | 1 |  |
| Bacteria;Proteobacteria;Betaproteobacteria;Burkholderiales;Comamonadaceae;Variovorax; | 0 | 0 | 0 | 1 |  |
| Bacteria;Proteobacteria;Gammaproteobacteria;Enterobacterales;Enterobacteriaceae; | 0 | 0 | 3 | 0 |  |
| Bacteria;Proteobacteria;Gammaproteobacteria;Enterobacterales;Enterobacteriaceae;Enterobacter; | 0 | 0 | 3 | 0 |  |
| Bacteria;Proteobacteria;Gammaproteobacteria;Pseudomonadales;Pseudomonadaceae;Pseudomonas; | 3 | 0 | 0 | 0 |  |
| Bacteria;Proteobacteria;Gammaproteobacteria;Xanthomonadales;Xanthomonadaceae; | 0 | 0 | 0 | 2 |  |
| Bacteria;Proteobacteria;Gammaproteobacteria;Xanthomonadales;Xanthomonadaceae;Pseudoxanthomonas; | 0 | 0 | 0 | 8 |  |
|  |  |  |  |  |  |
| **Membrane adaptations - UDP-glucose-4-epimerase *exoB*** |  |  |  |  | 1 |
| Bacteria; | 53 | 0 | 6 | 12 |  |
| Bacteria;Actinobacteria; | 0 | 0 | 3 | 0 |  |
| Bacteria;Actinobacteria;Actinobacteria; | 0 | 7 | 15 | 33 |  |
| Bacteria;Actinobacteria;Actinobacteria;Corynebacteriales;Mycobacteriaceae;Mycobacterium; | 0 | 0 | 0 | 2 |  |
| Bacteria;Actinobacteria;Actinobacteria;Corynebacteriales;Nocardiaceae;Rhodococcus; | 20 | 0 | 0 | 0 |  |
| Bacteria;Actinobacteria;Actinobacteria;Micrococcales; | 3 | 0 | 0 | 2 |  |
| Bacteria;Actinobacteria;Actinobacteria;Micrococcales;Microbacteriaceae; | 3 | 7 | 64 | 3 |  |
| Bacteria;Actinobacteria;Actinobacteria;Micrococcales;Microbacteriaceae;Leucobacter; | 0 | 0 | 3 | 0 |  |
| Bacteria;Actinobacteria;Actinobacteria;Propionibacteriales;Propionibacteriaceae; | 37 | 0 | 0 | 0 |  |
| Bacteria;Actinobacteria;Actinobacteria;Propionibacteriales;Propionibacteriaceae;Cutibacterium; | 17 | 0 | 0 | 0 |  |
| Bacteria;Actinobacteria;Actinobacteria;Propionibacteriales;Propionibacteriaceae;Cutibacterium;Cutibacterium acnes; | 3 | 0 | 0 | 0 |  |
| Bacteria;Proteobacteria; | 13 | 0 | 6 | 19 |  |
| Bacteria;Proteobacteria;Alphaproteobacteria; | 3 | 0 | 0 | 0 |  |
| Bacteria;Proteobacteria;Alphaproteobacteria;Rhizobiales; | 7 | 4 | 0 | 0 |  |
| Bacteria;Proteobacteria;Alphaproteobacteria;Rhizobiales;unclassified Rhizobiales;unclassified Rhizobiales;Rhizobiales sp. | 7 | 0 | 0 | 0 |  |
| Bacteria;Proteobacteria;Alphaproteobacteria;Sphingomonadales;Sphingomonadaceae;Sphingomonas; | 3 | 0 | 0 | 0 |  |
| Bacteria;Proteobacteria;Alphaproteobacteria;Sphingomonadales;Sphingomonadaceae;Sphingopyxis; | 0 | 0 | 3 | 0 |  |
| Bacteria;Proteobacteria;Betaproteobacteria;Nitrosomonadales;Thiobacillaceae;Thiobacillus; | 0 | 0 | 0 | 4 |  |
| Bacteria;Proteobacteria;Gammaproteobacteria;Xanthomonadales; | 0 | 0 | 0 | 1 |  |
| Bacteria;Proteobacteria;Gammaproteobacteria;Xanthomonadales;Xanthomonadaceae;Pseudoxanthomonas; | 0 | 0 | 0 | 4 |  |
|  |  |  |  |  |  |
| **Membrane adaptations - membrane-bound lytic murein transglycosylase D** |  |  |  |  | 0.91 |
| Bacteria; | 3 | 0 | 0 | 7 |  |
| Bacteria;Proteobacteria; | 93 | 61 | 90 | 449 |  |
| Bacteria;Proteobacteria;Betaproteobacteria;Nitrosomonadales;Thiobacillaceae;Thiobacillus; | 0 | 0 | 0 | 4 |  |
